# Reconciling Electrostatic and n→π* Orbital Contributions in Carbonyl Interactions

**DOI:** 10.1002/anie.202005739

**Published:** 2020-07-01

**Authors:** Kamila B. Muchowska, Dominic J. Pascoe, Stefan Borsley, Ivan V. Smolyar, Ioulia K. Mati, Catherine Adam, Gary S. Nichol, Kenneth B. Ling, Scott L. Cockroft

**Affiliations:** ^1^ EaStCHEM School of Chemistry The University of Edinburgh Joseph Black Building David Brewster Road Edinburgh EH9 3FJ UK; ^2^ Syngenta Jealott's Hill International Research Centre Bracknell Berkshire RG42 6EY UK

**Keywords:** computational chemistry, conformation analysis, delocalization, electrostatic interactions, noncovalent interactions

## Abstract

Interactions between carbonyl groups are prevalent in protein structures. Earlier investigations identified dominant electrostatic dipolar interactions, while others implicated lone pair n→π* orbital delocalisation. Here these observations are reconciled. A combined experimental and computational approach confirmed the dominance of electrostatic interactions in a new series of synthetic molecular balances, while also highlighting the distance‐dependent observation of inductive polarisation manifested by n→π* orbital delocalisation. Computational fiSAPT energy decomposition and natural bonding orbital analyses correlated with experimental data to reveal the contexts in which short‐range inductive polarisation augment electrostatic dipolar interactions. Thus, we provide a framework for reconciling the context dependency of the dominance of electrostatic interactions and the occurrence of n→π* orbital delocalisation in C=O⋅⋅⋅C=O interactions.

## Graphical abstract

**Carbonyls reconciled**: Electrostatics and orbital interactions have both been implicated in governing carbonyl interactions. A combined experimental and computational approach reconciles these conflicting explanations of the physiochemical origin of the interaction, demonstrating that orbital delocalisation augments electrostatic control, but for very close carbonyl contacts.

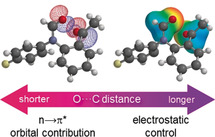

## Introduction

Carbonyl groups are prevalent throughout chemistry and biology. Interactions involving carbonyl groups are crucial in molecular recognition processes^1^ and play a key role in determining the conformation of small molecules,^2^ proteins,^3^ and peptides.^4^ Despite the apparent importance of C=O⋅⋅⋅C=O interactions, their physicochemical origin remains the subject of significant debate. Attractive interactions between an oxygen atom of a carbonyl group and the carbon atom of another were first evidenced in the crystal structures of small, carbonyl‐rich molecules in the 1950s.^5^ In all these cases the length of the C=O⋅⋅⋅C=O contact was less than the sum of the van der Waals radii, and in some cases, C=O⋅⋅⋅C=O interactions would even form in preference to C=O⋅⋅⋅HN hydrogen bonds.5d Indeed, the structures of α‐helices and β‐sheets are determined not only by C=O⋅⋅⋅HN hydrogen bonds, but also by competitive C=O⋅⋅⋅C=O attractive forces, which account for the characteristic sheared displacement of interacting peptide chains.3b, 3c

Carbonyl interactions were initially considered to be driven by electrostatics. Orthogonal C=O⋅⋅⋅C=O dipolar interactions can occur between the electron‐rich oxygen atom of one carbonyl group and the partial positive charge of the carbon atom of another.1a, 6 Conversely, it has also been suggested that favourable C=O⋅⋅⋅C=O interactions may involve the delocalisation of electron density (also known as induction, polarisation, orbital interactions, or stereoelectronic effects)^7^ from the lone pair (n) of a carbonyl donor into the antibonding (π*) orbital of an acceptor carbonyl, and denoted as an n→π* interaction.2d, 2e, 3a, 3f, 3g, 8 Crystallographic and conformational analyses have examined the distance and angle preferences of close carbonyl contacts to posit that C=O⋅⋅⋅C=O interactions may occur through n→π* interactions without requiring dipolar interactions.^9^ Furthermore, n→π* orbital delocalisation in carbonyl interactions has recently been further demonstrated to stabilise the transition state of molecular rotors.^7^ Indeed, this n→π* delocalisation has been increasingly exploited for kinetic reaction selectivity8a, 10 and influencing dynamic covalent equilibria.^11^

Despite the recent strong evidence for n→π* delocalisation from a range of experimental and theoretical studies, these results remain unreconciled with earlier studies that indicated an electrostatically driven interaction.1a, 6 Indeed, both the electrostatic and orbital delocalisation models qualitatively account for the directionality of orthogonal C=O⋅⋅⋅C=O interactions resembling the Bürgi–Dunitz nucleophile–carbonyl trajectory.^12^ Moreover, both the competing dipolar and orbital interaction models of C=O⋅⋅⋅C=O interactions are supported by analyses of quantitative experimental data obtained using different families of molecular torsion balances.2d, 2e, 3f, 6, 10b, 13

**Figure 1:**
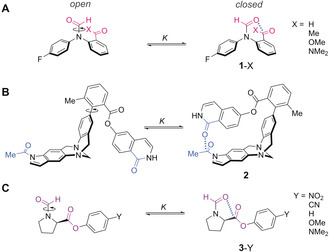
Molecular balances examined in the present investigation of C=O⋅⋅⋅C=O interactions. A) Newly designed balance series **1**‐X. B) The balance **2** previously reported by Diederich.^6^, ^13^ C) Balance series **3**‐Y previously reported by Raines.2e

**Figure 2:**
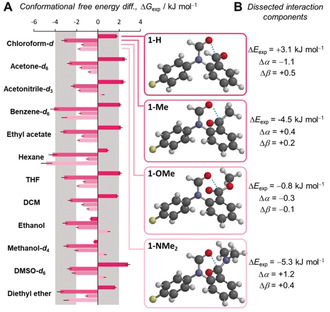
A) Experimental conformational free energies (Δ*G*_exp_) measured in 12 different solvents by ^19^F{^1^H} NMR spectroscopy (376.5 MHz, 298 K). Negative Δ*G*_exp_ values are defined as a preference for the closed conformation. Corresponding minimised structures (B3LYP/6‐31G*) of each molecular balance calculated in the gas phase are shown. Structures minimised using ωB97X‐D/6‐31G* showed minimal change from B3LYP/6‐31G* structures (see Figure S46). X‐ray structures are given in Section S2.4. All data and errors are tabulated in Table S16. B) Dissected interaction components using Hunter's α/β hydrogen‐bond model^21^ (see Section S3.3).

**Figure 3:**
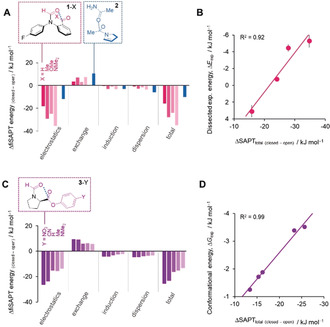
Total and dissected interaction energies between the coloured functional groups calculated using fiSAPT and SAPT for A) the **1**‐X series (fiSAPT) and **2** (SAPT) and C) the **3**‐Y series (fiSAPT).^24^ SAPT calculations for **2** used the isolated fragment of the known X‐ray structure shown.^6^ B) Calculated differences in the total fiSAPT energies of the interactions between the coloured structural fragments in the open and closed conformers (Δ*S*APT_total_) correlate with both the empirically dissected intramolecular interaction energy difference between the open and closed conformers of series **1**‐X (Δ*E*_exp_ from Figure 2 B), and also D) experimental conformational energy differences for series **3**‐Y measured in CDCl_3_ (Δ*G*_exp_).2e Calculations performed using PSI4^18^ at SAPT0/6‐311G* on B3LYP/6‐31G* minimised geometries. Alternative calculations and minimisations performed using additional diffuse and polarisation functions provided similar results (ωB97X‐D/6‐31G* and B3LYP/6‐311+G**, jun‐cc‐pVDZ, aug‐cc‐pVQZ, see Section S4.3, Supporting Information).

**Figure 4:**
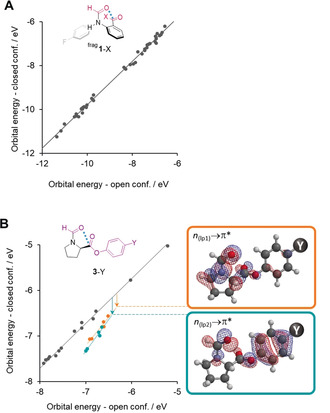
Correlation of calculated orbitals energies in the open vs. closed conformers of A) balance series **1**‐X and B) Balance series **3**‐Y. The second aromatic ring in balance series **1**‐X was replaced with a proton to give ^frag^ **1**‐X to avoid orbital splitting arising from the canonical resonance forms of the aromatic electrons (see Section S4.5). Data points that fall below the trend formed by grey points are stabilised in the closed conformer due to the n→π* electron delocalisation from both lone pairs of the carbonyl donor. For balance series ^frag^ **1**‐X, no special stabilisation of orbitals between the open and closed conformations was observed, while for balance series **3**‐Y two sets of molecular orbitals (orange and teal) were observed corresponding to stabilisation of the carbonyl oxygen lone pairs into the adjacent carbonyl group via n→π* interactions.

**Figure 5:**
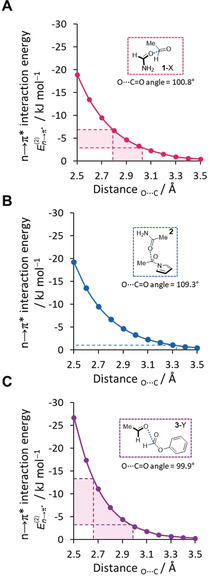
Total second‐order perturbation energies corresponding to n→π* electron delocalisation determined for both lone pairs in simplified models of the C=O⋅⋅⋅C=O interactions hosted within balance series A) **1**‐X, B) **2**, and C) **3**‐Y. Energies were calculated using NBO6.0, see Section S4.4, Supporting Information for details. Deflection angles from the plane of the acceptor carbonyl are indicated (e.g. Bürgi–Dunitz angle=107°).^12^ Shaded areas correspond to O⋅⋅⋅C distances observed in the respective balance series (see Table S20).

Here we set out to determine whether it is possible to reconcile the competing electrostatic and orbital‐based models of C=O⋅⋅⋅C=O interactions. The nature of the interaction was examined in different contexts in which interaction geometries and solvents were varied. We synthesised a new series of molecular torsion balances to quantify a range of C=O⋅⋅⋅C=O interactions (Figure 1). Carbonyl interactions were screened in 12 different solvents to enable an empirical dissection of the intramolecular carbonyl interaction from the modulating influence of the solvent effects (Figure 2). Theoretical fiSAPT energy partitioning was used to compare the electrostatic, exchange, dispersion, and induction (orbital) components in different contexts (Figure 3). Finally, the geometry‐dependent extent to which orbital delocalisation augments electrostatic C=O⋅⋅⋅C=O interactions was examined (Figure 4 and Figure 5).

## Results and Discussion

### Experimental Evaluation of Carbonyl Interactions

Molecular balances are useful tools for the quantitative study of interactions and their associated solvent effects, since the conformational equilibrium position is determined by differences in the intramolecular interactions and relative solvation energies of each conformer.^14^ For example, the molecular balance structures shown in Figure 1 accommodate C=O⋅⋅⋅C=O interactions in the closed conformer (right) that are absent in the open conformer (left). The groups of Diederich and Raines previously employed the molecular balances **2** 6, 13 and **3**‐Y2e (Figure 1 B and C), respectively, to study C=O⋅⋅⋅C=O interactions, but came to different conclusions with regard to the major energetic contributions.2d, 2e, 3f, 6, 13 Diederich determined the carbonyl interaction in **2** to be driven by electrostatics, while Raines found n→π* orbital delocalisation between carbonyl groups to play an important role in the **3**‐Y series of balances. To investigate the apparent incongruity regarding the nature of carbonyl interactions we devised a new series of molecular balances, **1**‐X, to supplement the existing datasets (Figure 1 A). The formamide balance series **1** is derived from molecular balances previously used to study solvent effects,14a, 15 H‐bonding,^16^ and chalcogen bonding interactions.^17^ The minimal design of series **1**‐X simplifies the interpretation of experimental data and computational analysis of the experimentally determined conformational preferences. Since rotation around the formamide bond is slow on the NMR timescale, discrete peaks corresponding to the open and closed conformers can be observed. Thus, integration of the conformer peaks provides direct access to the conformational equilibrium constant, *K*, which can be used to determine the conformational free‐energy difference, Δ*G*_exp_=−*RT* ln *K*.

The molecular balances in series **1**‐X were synthesised as described in Section S2.2 of the Supporting Information (Figure 1 A). The occurrence of C=O⋅⋅⋅C=O contacts (between the formamide oxygen and each X‐substituted carbon, Figure 2) in the closed conformer of each balance was confirmed computationally (B3LYP/6‐31G* and ωB97X‐D/6‐31G*, Section S4.1), and by X‐ray crystallography for **1**‐H and **1**‐Me (see Section S2.4, Deposition Numbers 1871050, and 1871051  contain the supplementary crystallographic data for this paper. These data are provided free of charge by the joint Cambridge Crystallographic Data Centre and Fachinformationszentrum Karlsruhe Access Structures service www.ccdc.cam.ac.uk/structures.). Solution‐phase conformers were assigned by HMBC and NOESY NMR spectroscopy (see Section S2.3), and conformational free‐energy differences were measured by ^19^F{^1^H} NMR spectroscopy in 12 different solvents (Figure 2; see Section S3.1).

**1**‐H generally favoured the open conformer where no C=O⋅⋅⋅C=O contact could be formed (+1 to +2 kJ mol^−1^). However, **1**‐Me, **1**‐OMe, and **1**‐NMe_2_ favoured the closed conformer, in which a C=O⋅⋅⋅C=O contact was formed in most solvents (−4 to −1 kJ mol^−1^). In contrast, a series of structurally similar balances, but bearing non‐carbonyl *ortho*‐substituents, generally favoured the open conformer (see Sections S2.1 and S3.1). Contrasting with prior examinations of substituent effects in formamide molecular balances,14a the experimentally determined Δ*G*_exp_ values for series **1**‐X correlated poorly with calculated electrostatic potentials over the X‐substituted aromatic ring (see Figures S30 and S31). The above observations are consistent with intramolecular interactions between the carbonyl groups playing a major role in governing the equilibrium position of the balance series **1**‐X. Further supporting this assertion, noncovalent interaction (NCI) plots confirmed attractive interactions between the carbonyl groups (see Figure S63).

### Evaluation of Electrostatic Solvent Effects

Solvents exert important influences on the conformational preferences of molecular balances.14b, 16, 19 However, the conformational free‐energy differences of the **1**‐X series showed only moderate solvent dependence, with similar conformational free‐energy differences in polar and apolar solvents (Figure 2). This level of solvent independence is surprising given that the conformational free energies of closely related formamide molecular balances were more strongly dependent on the hydrogen‐bond donor and acceptor ability of the solvent.14a Moreover, energetic solvent independence may be indicative of the presence of significant electron delocalisation in the interaction, and could indicate the small significance of electrostatic forces in C=O⋅⋅⋅C=O interactions.^17^, ^20^

The experimentally determined Δ*G*_exp_ values correlated moderately with some solvent parameters (see Section S3.2), but the best insights were gained using Hunter's α/β hydrogen‐bond model.^21^ The same approach has previously been shown to account for solvent competition in the conformational equilibria of molecular balances,14b, 19a, 22 and involves iterative least‐squares fitting of the experimentally obtained Δ*G*_exp_ values against those predicted by the model as the hydrogen‐bond donor and acceptor properties of the solvent are varied (see Section S3.2). The resulting dissected differences in the intramolecular interaction energy (Δ*E*_exp_) and corresponding changes in the hydrogen‐bond donor and acceptor constants (Δα and Δβ) between the open and closed conformers are listed Figure 2 B. The Δα and Δβ values indicate the extent to which competitive hydrogen‐bonding interactions with the solvent attenuate the intramolecular electrostatic interactions between the carbonyl groups. Most significantly, the empirically dissected solvent‐independent intermolecular interaction energies Δ*E*_exp_ correlated well (R^2^=0.92) with the change in the interaction energies occurring between the carbonyl groups (structural fragments highlighted in pink in Figure 3 A) upon flipping from the open to the closed conformation, as calculated using fiSAPT (Δ*S*APT_total_ in Figure 3 B; see Section S4.3 and discussion below).24b

### Dissecting the Origin of C=O⋅⋅⋅C=O Interactions

Symmetry adapted perturbation theory (SAPT) is a powerful computational tool for examining molecular interactions.^24^ Encouraged by the aforementioned correlation between experiment and theory for series **1**‐X (Figure 3 B), we expanded our use of functional‐group intramolecular SAPT (fiSAPT)24b to examine the **3**‐Y series (Figure 3 C,D; see Section S4.3). Once again, an excellent correlation (R^2^=0.99) was found between the experimental conformational energies (Δ*G*_exp_)2a and the change in the total fiSAPT interaction energy between the carbonyl groups (highlighted in purple in Figure 3 C) upon flipping from the open to the closed conformation (Δ*S*APT_total_, Figure 3 D). The Δ*S*APT_total_ energies calculated in the gas phase were much more favourable than the corresponding experimentally determined conformational energies (Δ*E*_exp_ and Δ*G*_exp_). This difference likely provides an indication of the magnitude of the attenuating influence of the solvent, both in terms of competitive dispersion^23^ and electrostatic interactions.^12^, ^15^

Moreover, the SAPT approach facilitated the energetic dissection of electrostatics, induction (which includes n→π* electron delocalisation), dispersion, and exchange repulsion to the carbonyl interactions of interest (Figures 3 A and C).^24^ Only small variations in the exchange, induction, and dispersion components across series **1**‐X and **3**‐Y were observed. The largest variation was found in the electrostatic term, which accordingly makes a dominant contribution to the total SAPT energy of the carbonyl interactions in both series.

Interestingly, both the experimental and calculated substituent effect trends are reversed in series **1**‐X compared to series **3**‐Y. The trend makes most intuitive sense in series **3**‐Y where the C=O⋅⋅⋅C(=O)Y interactions are most stabilised by electron‐withdrawing Y substituents. The different trends can be rationalised by secondary interactions occurring alongside the C=O⋅⋅⋅C=O interactions in series **1**‐X. Firstly, the electrostatic interaction between the carbonyl groups is less favourable in **1**‐H, which has a conformational minimum in which the dipoles of the carbonyl groups repel one another (top right, Figure 2 A; Figure S49 and Section S4.3). In contrast, the carbonyl groups attached to the phenyl ring in the other **1**‐X balances are flipped relative to **1**‐H and instead form favourable dipolar interactions. Secondly, the carbonyl–carbonyl contacts in **1**‐Me and **1**‐NMe_2_ appeared to be more stabilised than in **1**‐OMe. Noncovalent interaction (NCI) plots (see Figure S63)^27^ indicated that additional secondary interactions between the formyl oxygen atom and the methyl groups in both **1**‐Me and **1**‐NMe_2_ account for the additional electrostatic (and to a lesser extent, inductive and dispersion) stabilisation observed in the fiSAPT dissection (Figures 3 A and C). Hence, the secondary C=O⋅⋅⋅H_3_C interactions in **1**‐Me and **1**‐NMe_2_, combined with the flipped orientation of the carbonyl group in **1**‐H, account for the apparently inverted electronic trend observed both experimentally and theoretically in series **1**‐X compared to series **3**‐Y.

While the fiSAPT analysis and correlations against experimental data presented in Figure 3 appear reasonable, we caution that such energetic dissections are nonphysical, especially when performed intramolecularly across covalent bonds in the fiSAPT variant.^24^ Contrasting with the tightly constrained intramolecular geometries of the balance series **1**‐X and **3**‐Y, the larger folding structure of **2** facilitated intermolecular SAPT analysis on a fragment of the known X‐ray structure^6^ of **2** (Figure 3 A, blue). Reassuringly, the energetic composition of the carbonyl interactions in **2** calculated using SAPT was remarkably similar to those calculated for the **1**‐X and **3**‐Y series using fiSAPT (Figure 3 A and C, blue vs. pink and purple). Consistent with the SAPT analysis above, Diederich concluded that the carbonyl interactions in **2** were best described as orthogonal dipolar interactions.^6^, ^13^ Given Raines’ extensive evidence supporting the importance of n→π* delocalisation in carbonyl interactions, we were surprised to find that the induction contribution to the conformational preferences of series **3**‐Y was only slightly greater than that seen for series **1**‐X.

### Reconciling the Observation of n→π* Orbital Contributions

Having found surprisingly similar behaviour in the carbonyl interactions of all three series of balances, we next sought to reconcile the observation of n→π* orbital contributions. The simple designs of series **1**‐X and **3**‐Y makes it relatively easy to calculate and identify any molecular orbitals that are stabilised on changing from the open to the closed conformer. Thus, orbital energies were calculated for the open and closed conformers and plotted against each other for the balance series **1**‐X and **3**‐Y (Figures 4 A and B; see Section S4.5). No points deviate from the correlation in Figure 4 A, indicating no specific stabilisation of any orbitals in the closed conformers of the **1**‐X series. In contrast, two classes of molecular orbitals were found to be stabilised in the closed conformers of the **3**‐Y series (orange and teal points Figure 4 B). Visualisation of these molecular orbitals revealed that they corresponded to delocalisation of both oxygen lone pairs on the formyl carbonyl into the adjacent carbonyl in the closed conformer (i.e. n→π* interactions, Figure 4 B, right). The occurrence of n→π* delocalisation was confirmed using natural bonding orbital (NBO) calculations (see Section S4.4).^17^ Second‐order perturbation energies corresponding to these NBOs were calculated to contribute up to 9.4 kJ mol^−1^. In contrast, corresponding stabilising NBOs were not found in either **2** or the **1**‐X series (except for **1**‐Me in certain contexts; see Section S4.4). Similarly, pyramidalisation of the acceptor carbon atom (akin to the formation of a partial covalent bond) provides irrefutable evidence of n→π* delocalisation in the **3**‐Y series of balances.2e However, no such pyramidalisation was observed in any of the calculated or X‐ray structures of either **1**‐H or **1**‐Me (see Sections S2.4 and S4.1).

Overall, evidence from SAPT calculations (Figure 3), orbital‐energy stabilisation (Figures 4 A and B), NBOs (see Section S4.4), and carbonyl pyramidalisation2e all point to the occurrence n→π* electron delocalisation in series **3**‐Y, but not for series **1**‐X or **2**. These differences suggest that such orbital delocalisation is geometry dependent. Indeed, the C=O⋅⋅⋅C=O interactions in series **1**‐X have a 1,6‐relationship, while those in series **3**‐Y (and peptides) have a 1,5‐relationship (and have orbital interactions). Similarly, C=O⋅⋅⋅chalcogen bonds with a 1,5‐relationship are known to have important energetic orbital contributions (n→σ*), while those with 1,6‐relationships may not.^17^, ^25^

### Geometric Influences on n→π* Orbital Contributions

We next set out to examine the geometric dependency on the nature of C=O⋅⋅⋅C=O interactions. Molecular balances from the **3**‐Y series contained much closer C=O⋅⋅⋅C=O contacts than those in **2**, while those of series **1**‐X lay between the two extremes (see Table S20). Thus, we reasoned that closer carbonyl contacts could facilitate better orbital overlap, and therefore the occurrence of orbital delocalisation. Indeed, Shimizu and co‐workers recently found that the intramolecular stabilisation of transition states through n→π* interactions is strongly distance dependent.^7^

Sahariah and Sarma previously performed a computational examination on the geometry dependence of carbonyl interactions,^26^ however, this prior work focused on angular dependence rather than separation distance. Hence, for our calculations, the relative geometry of the C=O⋅⋅⋅C=O interactions in **1**‐Me, **2** and **3**‐H were arbitrarily locked in place (based on minimised B3LYP/6‐31G* geometries), and the O⋅⋅⋅C distance systematically varied.

NBO calculations were performed at each separation and the resulting sum of the second‐order perturbation energies corresponding to n→π* delocalisation of both carbonyl lone pairs was plotted (Figure 5; see Section S4.4). The second‐order perturbation energies of these n→π* NBOs become increasingly favourable as the O⋅⋅⋅C distance decreases for all three balance models. The distance dependencies of the energies for the **1**‐X and **2** models were very similar, but notably several kJ mol^−1^ less stable than the **3**‐Y model at shorter separations. The dotted lines in Figure 5 correspond to the maximum and minimum O⋅⋅⋅C distances observed in each balance series **1**‐X, **2**, and **3**‐Y (see Table S20). The balance **2** had the longest O⋅⋅⋅C distance, and correspondingly, the weakest orbital contribution. Conversely, series **3**‐Y showed the shortest range of O⋅⋅⋅C contacts, and featured the strongest n→π* contribution. Meanwhile, the **1**‐X series hosted O⋅⋅⋅C interactions with intermediate distances lying between those found in **2** and the **3**‐Y series. Indeed, on examining the full balance structures of the **1**‐X series, weak stabilising n→π* NBOs were only observed in the X‐ray and ωB97X‐D/6‐31G* minimised structures of **1**‐Me, which were by far the shortest O⋅⋅⋅C contacts found in the **1**‐X series (see Table S20 and Figure S57). In addition, the energies of the n→π* delocalisation energies across all three balance series were also surprisingly insensitive to the angle of deflection between the plane of the acceptor carbonyl (e.g. Bürgi–Dunitz angle=107°),^12^ which was further confirmed by a systematic scan of interaction angles and additional SAPT analysis (see Figures S59, S60 and S56).^26^ This minimal angle dependency coupled with the strong distance‐dependency observed across three different balance models suggests that O⋅⋅⋅C separation is key to determining whether orbital delocalisation occurs alongside the ever‐present electrostatic stabilisation in carbonyl–carbonyl interactions.

## Conclusion

In summary, we have performed a combined experimental and theoretical investigation of carbonyl interactions in a range of contexts and solvents. Previous investigations into the nature of carbonyl interactions identified conflicting physiochemical origins for the interaction, implicating the dominance of either electrostatics^6^, ^13^ or orbital delocalisation.2d, 2e, 3f, 7 We supplemented the existing data sets based on **2** and series **3**‐X by synthesising a new molecular balance series, **1**‐X. Experimentally determined conformational free energies confirmed the presence of carbonyl contacts in balance series **1**‐X. The significance of electrostatics in determining the conformational preference was confirmed by applying Hunter's α/β hydrogen‐bond model^21^ across 12 solvents. Computational SAPT and fiSAPT analysis indicated that the carbonyl interactions in all three of the balance series were largely governed by electrostatics in the gas phase (Figure 3). A pairwise analysis of orbital energies indicated that carbonyl lone pairs were stabilised by n→π* delocalisation in series **3**‐Y, but not in either series **1**‐X or **2** in the geometries examined (Figure 4). The disparate occurrence of orbital interactions was reconciled by examining the influence of O⋅⋅⋅C separation distance using NBO calculations. NBOs indicated the occurrence of n→π* delocalisation for the short contacts within series **3**‐Y, but not for the longer‐range interactions occurring in **2**. The carbonyl–carbonyl distances in balance series **1**‐X were intermediate between those found in series **3**‐Y and balance **2**, but only the structures of **1**‐Me containing the shortest O⋅⋅⋅C distances were found to facilitate weak n→π* delocalisation. The distance dependency of the orbital delocalisation component has important consequences for molecular recognition in solution. The equilibrium separations of intermolecular solvent–solute contacts allow attenuation by electrostatic^14^, ^15^, ^21^ and dispersion interactions,^23^ but such intermolecular equilibrium separations may not be short enough to permit the solvent to compete with short‐range intramolecular orbital delocalisation. Such a situation may account for the ability of intramolecular stereoelectronic effects (i.e. orbital delocalisation) to exert conformational control even in the presence of solvent competition.^28^ However, it should be noted that the conformational preferences of derivatives of Raines’ balances have been found to be solvent dependent.^29^ Our results have implications in the design of molecular systems seeking to exploit such carbonyl interactions, particularly in protein design, where the physiochemical origins of specific carbonyl interactions may have far‐reaching consequences on structure and behaviour. Furthermore, similarly discordant physicochemical rationalisations have been reported for a range of other interactions, notably chalcogen bonding.^17^, ^25^ It seems plausible that similar distance‐dependent orbital contributions may contribute to other classes of interactions. Consequently, we hope that similar investigations will help to reconcile conflicting results and deepen the understanding of a broader range or molecular interactions.

## Conflict of interest

The authors declare no conflict of interest.

## Supporting information

As a service to our authors and readers, this journal provides supporting information supplied by the authors. Such materials are peer reviewed and may be re‐organized for online delivery, but are not copy‐edited or typeset. Technical support issues arising from supporting information (other than missing files) should be addressed to the authors.

Supplementary
