# Supplementary material for: Reconciling Electrostatic and n→π* Orbital Contributions in Carbonyl Interactions
Source: Angew Chem Int Ed Engl. 2020 Jul 1;59(34):14602–8. doi: 10.1002/anie.202005739 (PMC7496118; doi:10.1002/anie.202005739)
Supplement: Supplementary file 1 — Supplementary [file ANIE-59-14602-s001.pdf]

Angewandte Chemie
Eine Zeitschrift der Gesellschaft Deutscher Chemiker

Supporting Information

Reconciling Electrostatic and n→π* Orbital Contributions in Carbonyl Interactions

*Kamila B. Muchowska, Dominic J. Pascoe, Stefan Borsley, Ivan V. Smolyar, Ioulia K. Mati, Catherine Adam, Gary S. Nichol, Kenneth B. Ling, and Scott L. Cockroft**

anie_202005739_sm_miscellaneous_information.pdf

Contents

S1. General experimental procedures 2
S2. Molecular torsion balances investigated 3
  S2.1 Full structures of all balances considered 3
  S2.2 Synthetic procedures and standard characterisation data 4
  S2.3 Conformer assignment using NMR spectroscopy 23
  S2.4 Crystal Structures of 1-H and 1-Me 24
S3. Experimental conformational free energies, ΔG_{exp} 45
  S3.1 Experimental determination conformational free energies, ΔG_{exp} 45
  S3.2 Correlations of solvent parameters with ΔG_{exp} 49
  S3.3 Application of the Hunter solvation model 53
S4. Computational methods and data 58
  S4.1 Geometry minimisation and calculated conformational free energies 58
  S4.2 Electrostatic Surface Potentials 64
  S4.3 fiSAPT analysis 65
  S4.4 Natural Bond Orbital analysis 78
  S4.5 Molecular orbital analysis 86
  S4.6 Non-Covalent Interaction Plots 90
S5. References 91

S1. General experimental procedures

Unless stated otherwise, all chemicals were purchased from commercial sources (Sigma Aldrich UK, Acros UK, VWR UK or Fluorochem UK) and used without further purification. Dry solvents were obtained by means of a “Glass Contour” brand solvent purification system, where solvents were passed through filter columns and dispensed under an argon atmosphere. Flash column chromatography was performed using Geduran® Si60 (40-63 mm, Merck, Germany) as the stationary phase, and thin-layer chromatography (TLC) was performed on pre-coated silica gel plates (0.25 mm thick, 60F254, Merck, Germany) and observed under UV light (l_{max} 254 nm). Mass spectrometry was performed by the University of Edinburgh technician-supported mass spectrometry service, using a Bruker micrOTOF II or ThermoElectron MAT XP spectrometer for EI or ESI-HRMS. Melting points were measured in a Gallenkamp melting point apparatus, and are reported uncorrected. ^{1}H and ^{13}C NMR spectra were recorded on Bruker Ultrashield 400 MHz, Bruker Ascend 500 MHz equipped with a DCH cryoprobe and Bruker Ascend 500 MHz with prodigy cryoprobe and Bruker Ultrashield 600 MHz with TCI cryoprobe, at a constant temperature of 25 °C, unless otherwise stated. ^{1}H, ^{13}C, ^{19}F and ^{195}Pt chemical shifts are reported in parts per million (ppm) from low to high field. ^{1}H and ^{13}C values are referenced to the literature values for chemical shifts of residual non-deuterated solvent, with respect to tetramethylsilane. ^{19}F is referenced externally to $CFCl_3$ at 0 ppm and ^{195}Pt is referenced externally to K_2PtCl_6 at 0 ppm. Standard abbreviations indicating multiplicity are used as follows: bs (broad singlet), d (doublet), dd (doublet of doublets), m (multiplet), q (quartet), s (singlet), t (triplet), tt (triplet of triplets), *J* (coupling constant). All spectra were analysed using MestReNova (Version 11.0.0). NMR tubes, precision glassware and glass syringes were dried under vacuum before use. Deuterated solvents were stood over oven-dried activated 4 Å molecular sieves for a minimum of 24 h prior to use. Non-deuterated anhydrous solvents were used directly as commercially obtained anhydrous solvents, or were redistilled under reduced pressure from analytical-grade solvents.

S2. Molecular torsion balances investigated

S2.1 Full structures of all balances considered

A **1**-X series - C=O...C=O interactions:

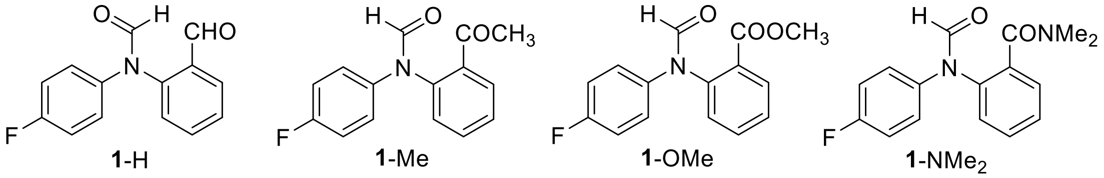

B Control series:

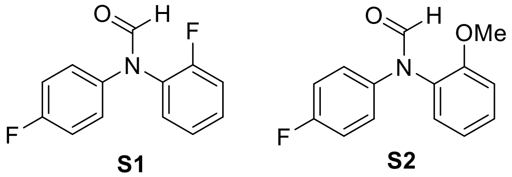

Figure S1 Structures of newly synthesised balance series **1**-X. An *ortho*-substituted control balance series, bearing non-carbonyl containing substituents was also synthesised. Balances **S2**-OMe and **S3**-Me were synthesised as previously reported.[S1] Characterisation data was in agreement with literature reported data.

A Deiderich's balance **2**

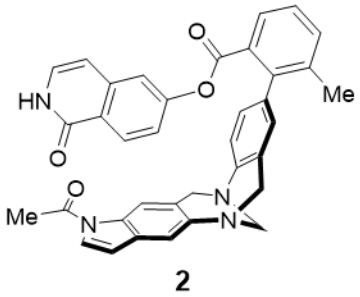

B Raines' balance series **3**-Y

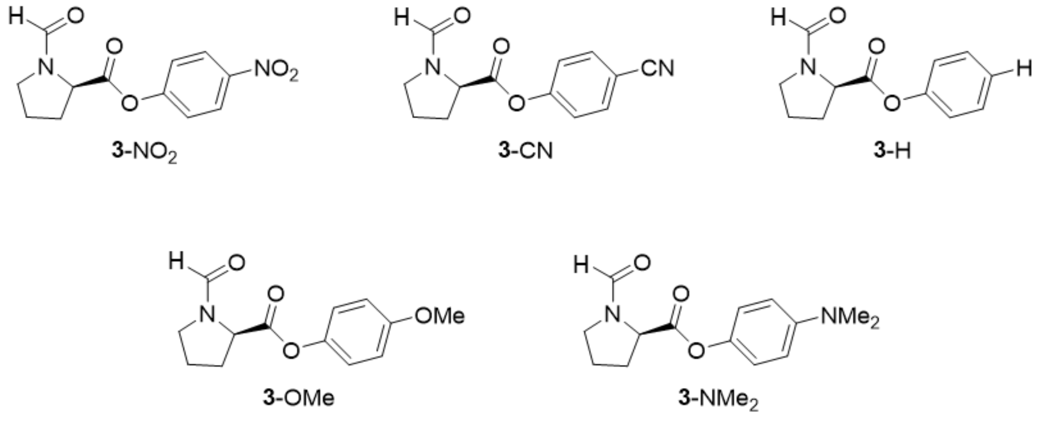

Figure S2 Structures of previously reported balances for studying carbonyl–carbonyl interactions. Balance **2** was previously reported by Diederich[S2] and balance series **3**-Y was previously reported by Raines.[S3]

S2.2 Synthetic procedures and standard characterisation data

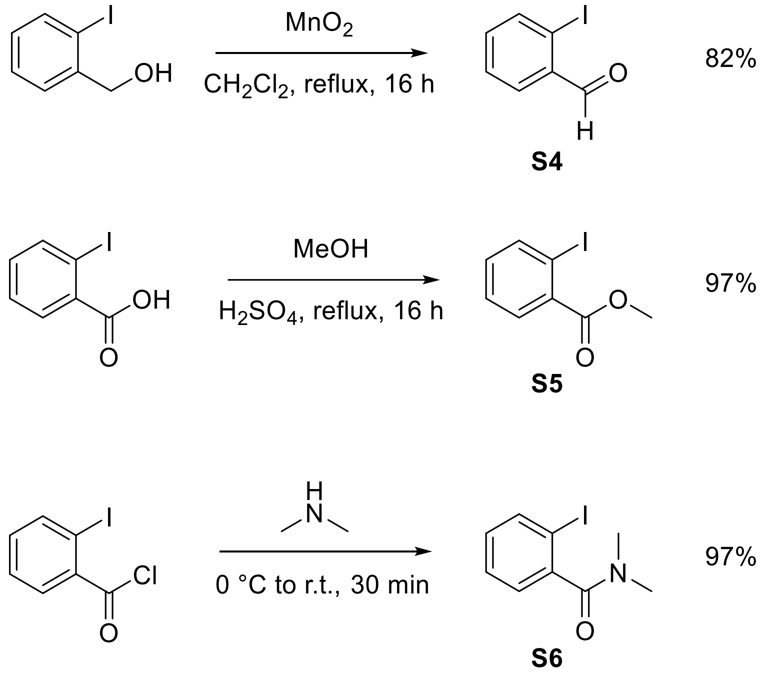

Figure S3 Synthesis of compounds **S4**–**S6**.

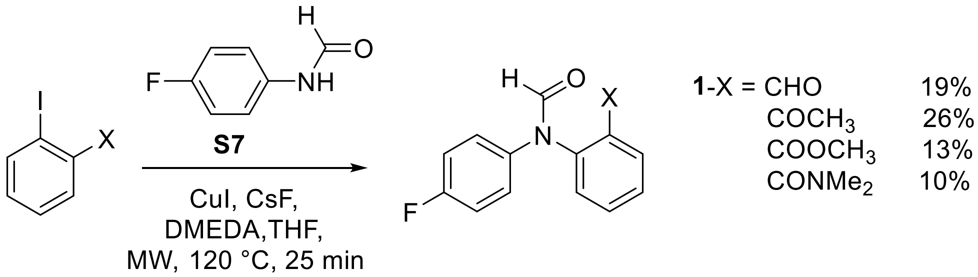

Figure S4 Microwave-assisted synthesis of balances **1**-H, **1**-Me, **1**-OMe and **1**-NMe_2. The synthesis of the starting iodoaryl compounds is outlined in **Figure S3**. 2-iodoacetophenone used for synthesising balance **1**-Me was commercially available. Amide **S7** was synthesised as previously reported.[S4] Characterisation data was in agreement with literature reported data.

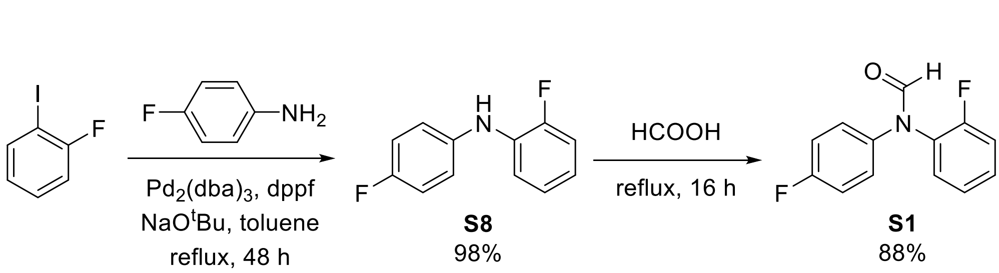

Figure S5 Synthesis of control balance **S1**.

2-Iodobenzaldehyde **S4**

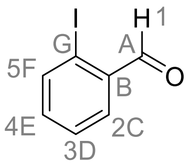

Manganese(IV) oxide (2.62 g, 30.1 mmol) was added to a solution of 2-iodobenzyl alcohol (1.00 g, 4.27 mmol) in CH_2Cl_2 (50 mL). The reaction mixture was heated to reflux overnight, filtered through celite and concentrated *in vacuo* to yield the product as a light yellow solid (0.812 g, 82%).

M. p. = 38–40 °C.

^{1}H NMR (500 MHz, $CDCl_3$) δ = 10.07 (H_1, d, J = 0.7 Hz, 1H), 7.95 (H_5, dd, J = 7.9, 1.0 Hz, 1H), 7.88 (H_2, dd, J = 7.7, 1.8 Hz, 1H), 7.46 (H_3, t, J = 7.5 Hz, 1H), 7.28 (H_4, td, J = 7.6, 1.7 Hz, 1H).

^{13}C NMR (126 MHz, $CDCl_3$) δ = 195.77 (C_A, s), 140.67 (C_F, s), 135.48 (C_E, s), 135.17 (C_B, s), 130.29 (C_C, s), 128.74 (C_D, s), 100.68 (C_G, s).

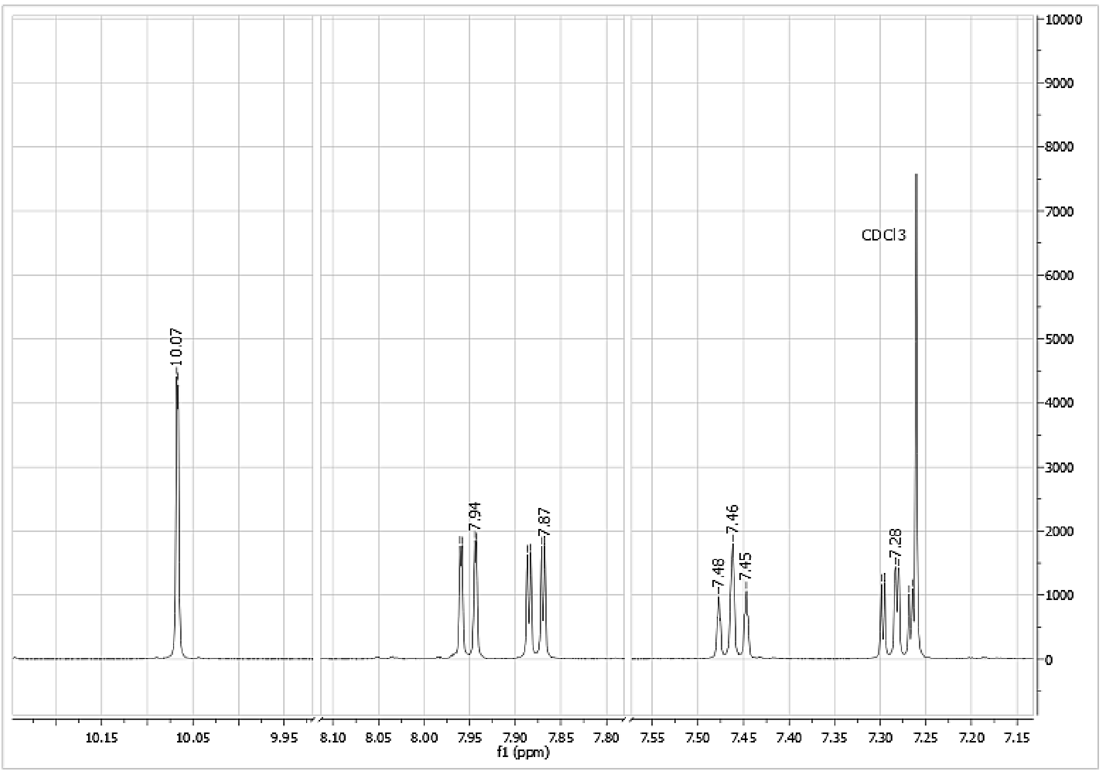

Figure S6 Partial ^{1}H NMR (500 MHz, 298 K, $CDCl_3$) of compound **S4**.

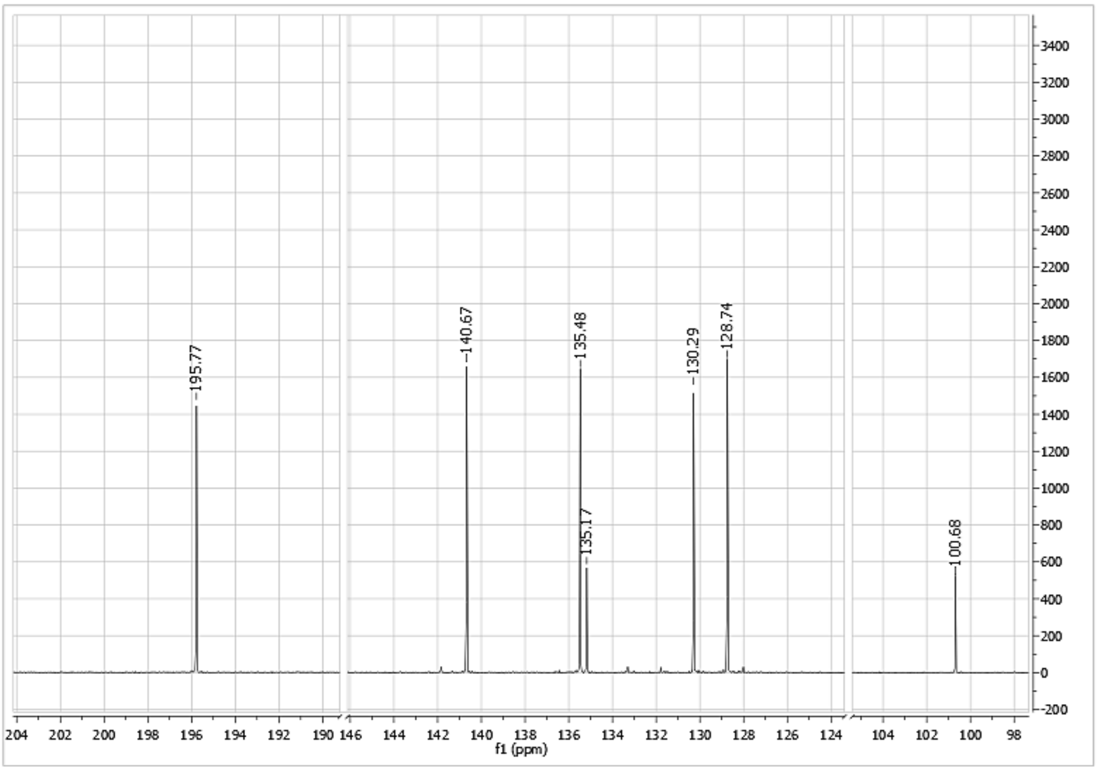

Figure S7 Partial ^{13}C NMR (126 MHz, 298 K, $CDCl_3$) of compound **S4**.

Methyl 2-iodobenzoate **S5**

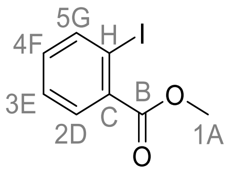

To a solution of 2-iodobenzoic acid (1.00 g, 4.03 mmol) in MeOH (25 mL), was added concentrated H_2SO_4 (1 mL) dropwise. The reaction mixture was heated to reflux overnight, then allowed to cool to room temperature. The mixture was poured into water (200 mL) and extracted with CH_2Cl_2 (3 × 30 mL). The organic layers were washed with saturated aqueous $NaHCO_3$ (3 × 30 mL) followed by brine (3 × 30 mL), dried over $MgSO_4$, filtered and concentrated *in vacuo*, to yield the product as a light yellow oil (1.023 g, 97%).

^{1}H NMR (500 MHz, $CDCl_3$) δ = 8.00 (H_5, dd, J = 8.0, 1.1 Hz, 1H), 7.80 (H_2, dd, J = 7.8, 1.7 Hz, 1H), 7.40 (H_3, td, J = 7.6, 1.2 Hz, 1H), 7.15 (H_4, td, J = 7.7, 1.7 Hz, 1H), 3.93 (H_1, s, 3H).

^{13}C NMR (126 MHz, $CDCl_3$) δ = 166.99 (C_B, s), 141.33 (C_G, s), 135.17 (C_C, s), 132.64 (C_F, s), 130.94 (C_D, s), 127.90 (C_E, s), 94.06 (C_H, s), 52.48 (C_A, s).

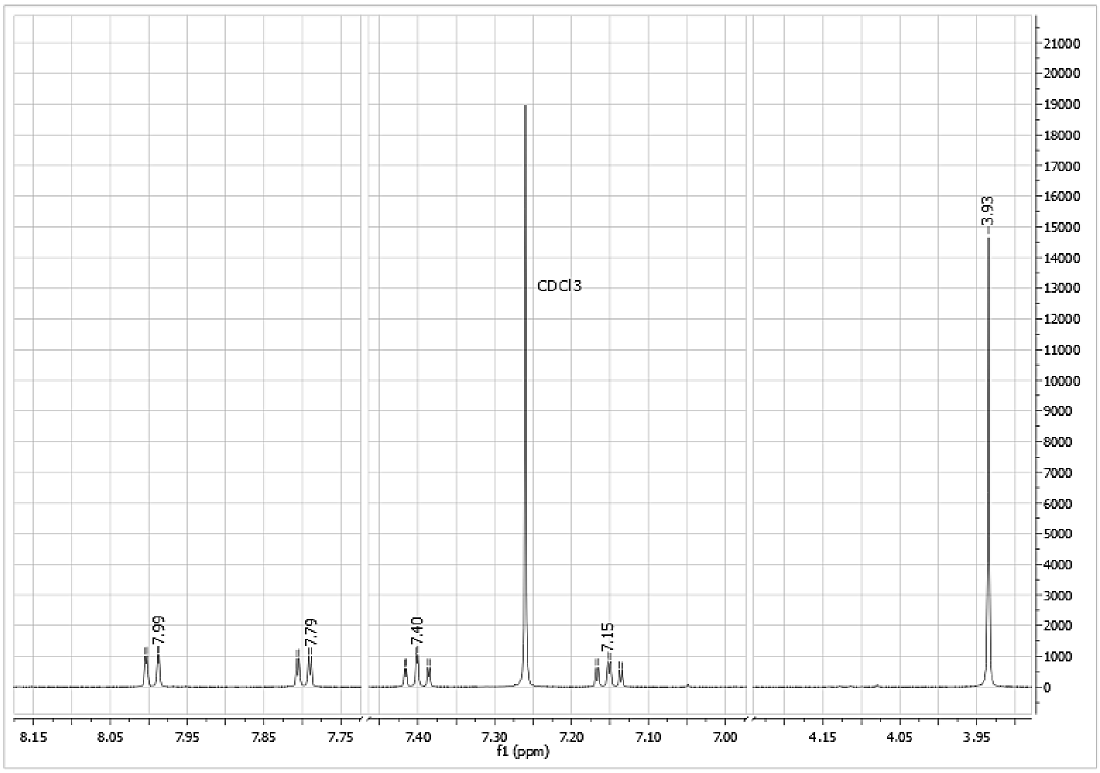

Figure S8 Partial ^{1}H NMR (500 MHz, 298 K, $CDCl_3$) of compound **S5**.

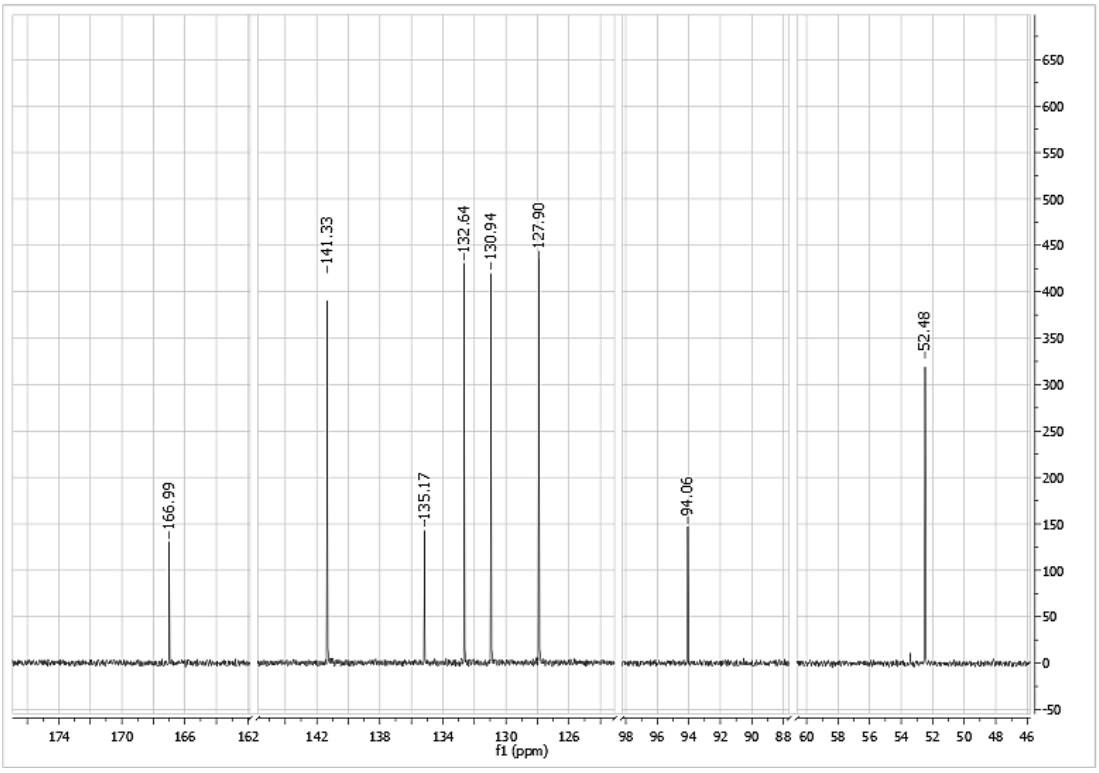

Figure S9 Partial ^{13}C NMR (126 MHz, 298 K, $CDCl_3$) of compound **S5**.

2-Iodo-*N,N*-dimethylbenzamide **S6**

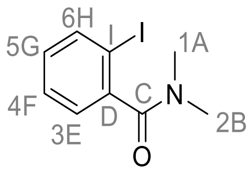

An aqueous solution of dimethylamine (40%, 40 mL) was added dropwise to 2-iodobenzoyl chloride (3.00 g, 11.26 mmol) at 0 °C. The reaction mixture was stirred at 0 °C for 30 min, quenched with a saturated aqueous solution of K_2CO_3 (100 mL), and extracted with CH_2Cl_2 (3 × 30 mL). The combined organics were dried over anhydrous $MgSO_4$, filtered and concentrated *in vacuo*. The oily residue was dried under high vacuum overnight to yield the product as a light yellow solid (3.010 g, 97%).

M. p. = 43–45 °C.

^{1}H NMR (500 MHz, $CDCl_3$) δ = 7.81 (H_6, dd, J = 8.0, 1.0 Hz, 1H), 7.38 (H_4, td, J = 7.5, 1.1 Hz, 1H), 7.20 (H_3, dd, J = 7.6, 1.6 Hz, 1H), 7.06 (H_5, ddd, J = 7.9, 7.6, 1.6 Hz, 1H), 3.13 (H_2, s, 3H), 2.84 (H_1, s, 3H).

^{13}C NMR (126 MHz, $CDCl_3$) δ = 170.77 (C_C, s), 142.86 (C_D, s), 139.10 (C_H, s), 130.08 (C_G, s), 128.42 (C_F, s), 127.05 (C_E, s), 92.42 (C_I, s), 38.43 (C_A, s), 34.73 (C_B, s).

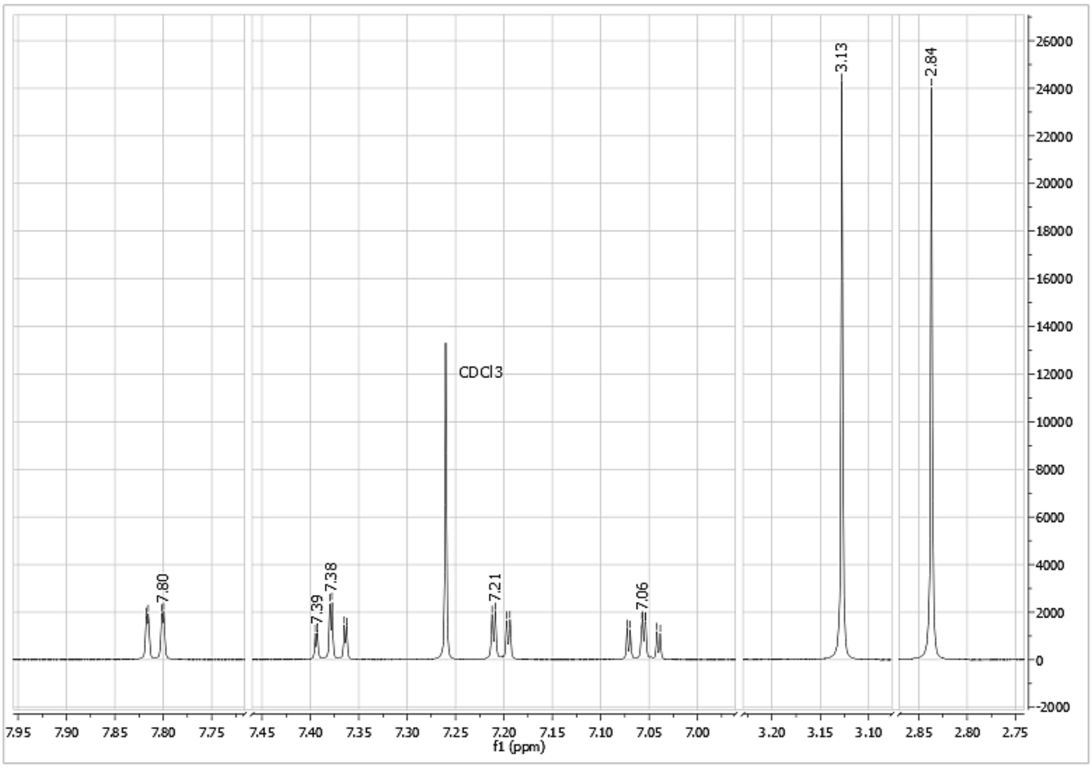

Figure S10 Partial ^{1}H NMR (500 MHz, 298 K, $CDCl_3$) of compound **S6**.

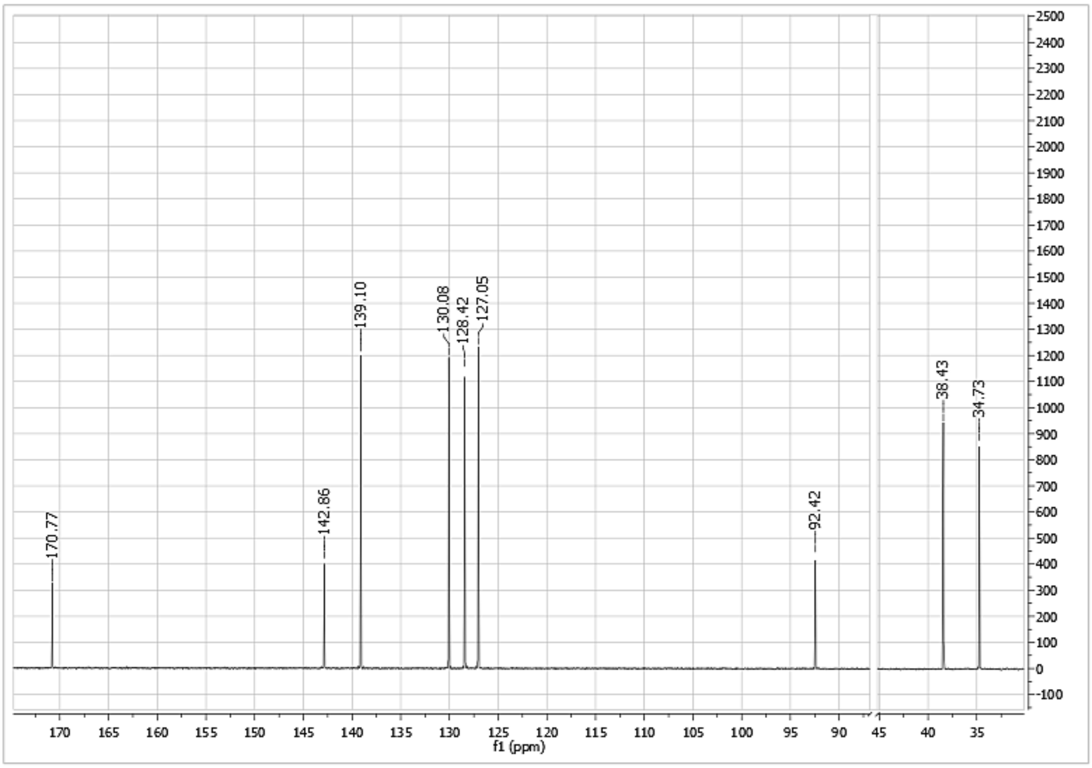

Figure S11 Partial ^{13}C NMR (126 MHz, 298 K, $CDCl_3$) of compound **S6**.

General procedure a: the microwave-assisted Goldberg-type copper(I)-catalysed C–N coupling

An over-dried microwaveable vial was charged with the formamide **S7** (typically approx. 200–300 g, 1.0 eq.),[S4] aryl iodide (1.2 eq.), copper(I) iodide (0.75 eq.), caesium fluoride (2.0 eq.) *N,N'*-dimethylethylenediamine (DMEDA) (0.10 eq.) and dry THF (typically approx. 2 mL). After sealing, the vial was stirred for 25 minutes at 120 °C under microwave irradiation. The reaction mixture was cooled to room temperature, diluted with CH_2Cl_2 (10 mL), quenched with aqueous saturated NH_4Cl (10 mL), and extracted with CH_2Cl_2 (3 × 10 mL). The combined organics were washed with brine, dried over $MgSO_4$, and the solvent was removed under a reduced pressure to yield a crude product, which was then subjected to purification as described individually.

N-(4-fluorophenyl)-*N*-(2-formylphenyl)formamide **1**-H

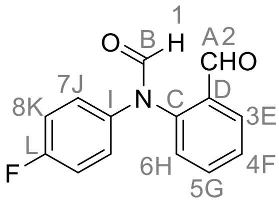

Prepared using general procedure **a**: Formamide **S7** (0.206 g, 1.48 mmol), 2-iodobenzaldehyde **S4** (0.400 g, 1.72 mmol), copper(I) iodide (0.141 g, 0.740 mmol), cesium fluoride (0.443 g, 2.91 mmol), DMEDA (0.150 mL, 0.170 mmol) and dry THF (2 mL). The crude product was purified by preparative TLC (EtOAc/hexane 1:1, *v*/*v*). The product was obtained as a yellow solid (0.0670 g, 19%).

M. p. = 68–71 °C.

^{1}H NMR (500 MHz, $CDCl_3$) δ = 10.15 (H_2', s), 10.09 (H_2, s), 8.77 (H_1, s), 8.54 (H_1', s), 8.05 (H_3', dd, J = 7.8, 1.6 Hz), 8.02 (H_3, dd, J = 7.7, 1.6 Hz), 7.76 (H_5', td, J = 7.7, 1.6 Hz), 7.71 (H_5, td, J = 7.7, 1.6 Hz), 7.62 (H_4', t, J = 7.6 Hz), 7.57 (H_4, t, J = 7.6 Hz), 7.36 (H_6', dd, J = 7.9, 0.7 Hz), 7.34–7.30 (H_7, m), 7.24 (H_6, dd, J = 7.9, 0.8 Hz), 7.20 – 7.16 (H_7', m), 7.16–7.11 (H_8, m), 7.11–7.07 (H_8', m).

^{13}C NMR (126 MHz, $CDCl_3$) δ = 189.00 (C_A, s), 188.78 (C_A', s), 162.12 (C_B, s), 161.57 (C_B', s), 161.32 (C_L, d, J = 248.1 Hz), 160.68 (C_L', d, J = 247.1 Hz), 142.22 (C_C', s), 140.02 (C_C, s), 137.61 (C_I, d, J = 3.1 Hz), 136.32 (C_I', d, J = 3.0 Hz), 135.43 (C_G', s), 135.14 (C_G, s), 132.36 (C_D', s), 132.16 (C_D, s), 131.20 (C_E', s), 130.90 (C_E, s), 129.61 (C_H', s), 129.12 (C_H, s), 129.07 (C_F', s), 128.76 (C_F, s), 126.40 (C_J', d, J = 8.3 Hz), 125.62 (C_J, d, J = 8.5 Hz), 116.87 (C_K, d, J = 23.0 Hz), 116.17 (C_K', d, J = 22.8 Hz).

^{19}F{^{1}H} NMR (471 MHz, $CDCl_3$) δ = −114.26 to −114.40 (m, major conformer), −114.53 to −114.64 (m, minor conformer′).

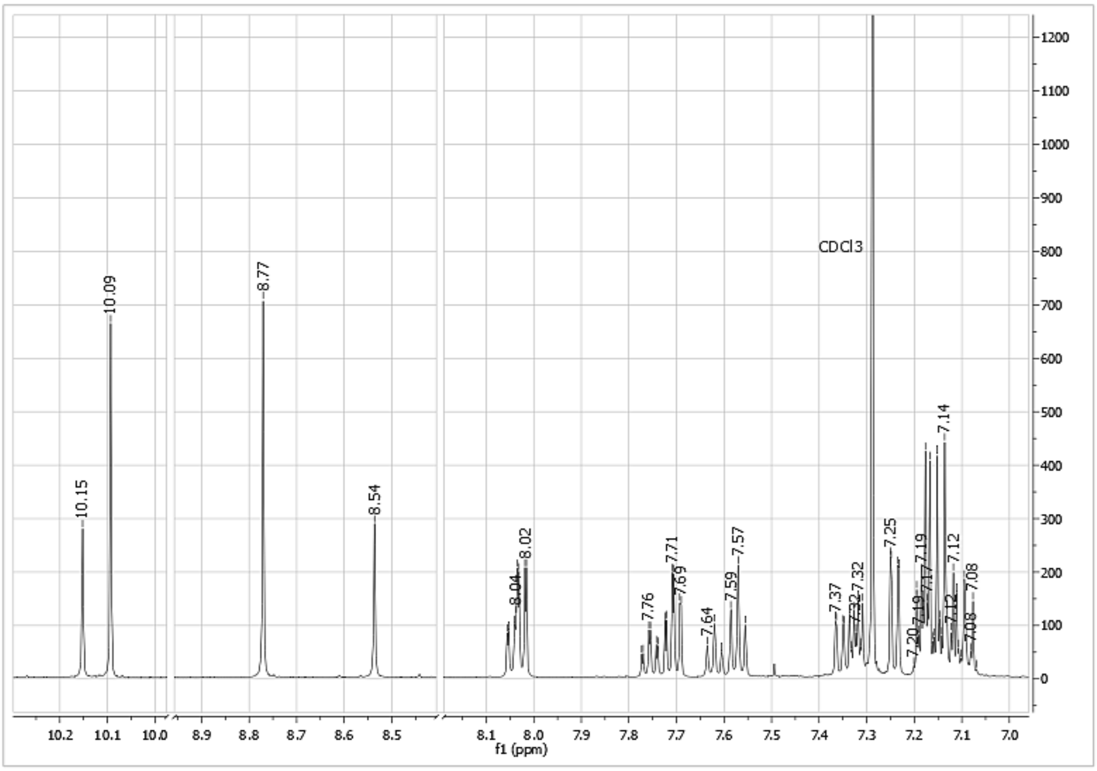

Figure S12 Partial ^{1}H NMR (500 MHz, 298 K, $CDCl_3$) of balance **1**-H.

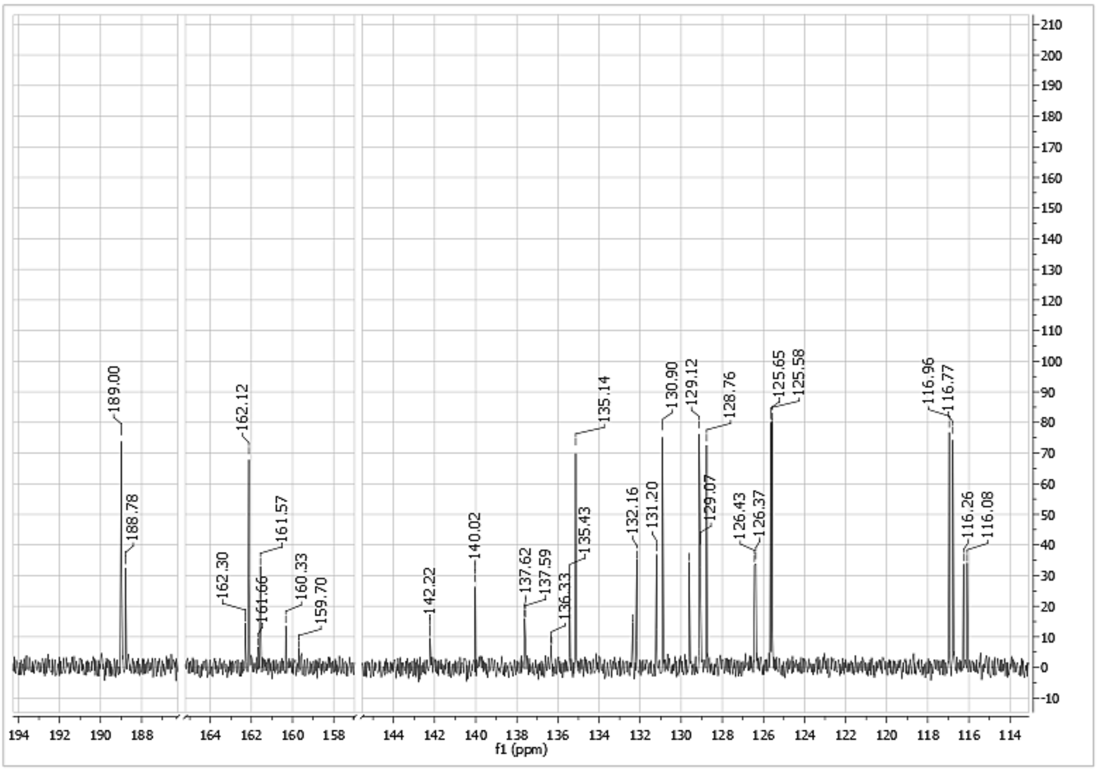

Figure S13 Partial ^{13}C NMR (126 MHz, 298 K, $CDCl_3$) of balance **1**-H.

N-(2-acetophenyl)-*N*-(4-fluorophenyl)formamide **1**-Me

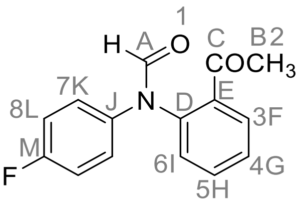

Prepared using general procedure **a**: Formamide **S7** (0.139 g, 1.00 mmol), 2-iodoacetophenone (0.285 g, 1.16 mmol), copper(I) iodide (0.095 g, 0.50 mmol), cesium fluoride (0.297 g, 1.96 mmol), DMEDA (0.12 mL, 0.12 mmol) and dry THF (2 mL). The crude product was purified by preparative TLC (EtOAc/hexane 1:1, *v*/*v*). The product was obtained as a yellow solid (0.0660 g, 26%).

M. p. = 97–99 °C.

^{1}H NMR (500 MHz, $CDCl_3$) δ = 8.45 (H_1, s), 8.43 (H_1', s), 7.72 (H_3', dd, J = 7.7, 1.5 Hz), 7.67 (H_3, dd, J = 7.7, 1.5 Hz), 7.59 (H_5', td, J = 7.7, 1.6 Hz), 7.52–7.46 (H_4', H_5, m), 7.40 (H_4, td, J = 7.6, 1.2 Hz), 7.30 (H_6', dd, J = 7.9, 1.0 Hz), 7.29–7.23 (H_7', H_7, m), 7.12–7.06 (H_8, H_6, m), 7.06–7.01 (H_8', m), 2.57 (2, s), 2.38 (H_2', s).

^{13}C NMR (126 MHz, $CDCl_3$) δ = 200.10 (C_C, s), 199.30 (C_C', s), 161.95 (C_A, s), 161.62 (C_A', s), 161.40 (C_M, d, J = 247.3 Hz), 160.52 (C_M', d, J = 246.4 Hz), 138.96 (C_D', s), 137.79 (C_J, d, J = 3.0 Hz), 137.61 (C_E, s), 137.29 (C_E', s), 136.27 (C_D, s), 135.79 (C_J', d, J = 2.8 Hz), 132.73 (C_H', s), 132.17 (C_H, s), 129.83 (C_F', s), 129.77 (C_I', s), 128.60 (C_I, s), 128.47 (C_G', s), 128.08 (C_F, s), 127.73 (C_G, s), 126.72 (C_K', d, J = 8.4 Hz), 126.60 (C_K, d, J = 8.5 Hz), 116.49 (C_L, d, J = 22.9 Hz), 115.78 (C_L', d, J = 22.8 Hz), 29.38 (C_B', s), 28.48 (C_B, s).

^{19}F{^{1}H} NMR (376 MHz, $CDCl_3$) δ = −114.71 (s, major conformer), −115.13 (s, minor conformer′).

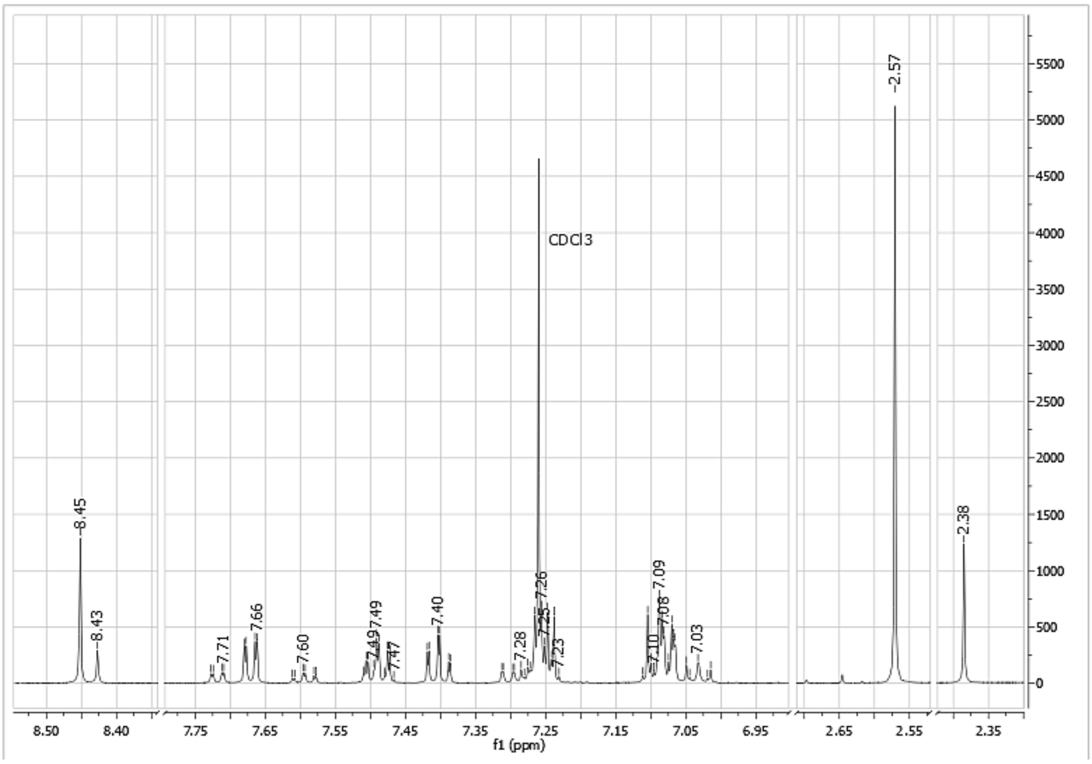

Figure S14 Partial ^{1}H NMR (500 MHz, 298 K, $CDCl_3$) of balance **1**-Me.

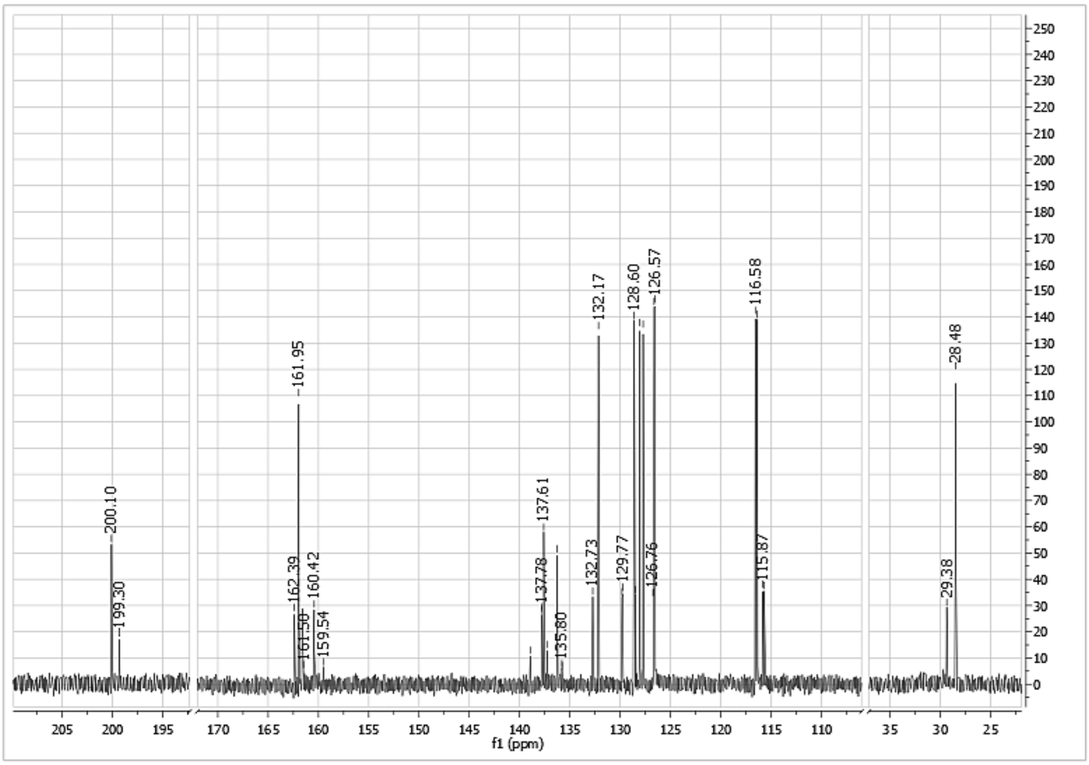

Figure S15 Partial ^{13}C NMR (126 MHz, 298 K, $CDCl_3$) of balance **1**-Me.

N-(4-fluorophenyl)-*N*-((2-methoxycarbonyl)phenyl)formamide **1**-OMe

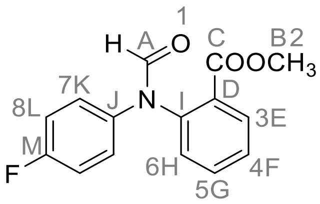

Prepared using the general procedure **a**: Formamide **S7** (0.274 g, 1.97 mmol), methyl 2-iodobenzoate **S5** (0.600 g, 2.29 mmol), copper(I) iodide (0188 g, 0.98 mmol), cesium fluoride (0.588 g, 3.87 mmol), DMEDA (0.250 mL, 0.230 mmol) and dry THF (2 mL). The crude product was purified by preparative TLC (EtOAc/hexane 5:2, *v*/*v*). The product was obtained as a light yellow oil (0.0720 g, 13%).

^{1}H NMR (500 MHz, $CDCl_3$) δ = 8.57 (H_1, s), 8.39 (H_1', s), 8.00 (H_3', dd, J = 7.9, 1.6 Hz), 7.98 (H_3, dd, J = 7.8, 1.5 Hz), 7.61 (H_5', td, J = 7.7, 1.6 Hz), 7.56 (H_5, td, J = 7.7, 1.6 Hz), 7.48 (H_4', td, J = 7.7, 1.2 Hz), 7.42 (H_4, td, J = 7.7, 1.2 Hz), 7.31–7.28 (H_6', m), 7.29–7.27 (H_7', m), 7.22–7.17 (H_7, m), 7.15 (H_6, dd, J = 7.9, 1.1 Hz), 7.11–7.06 (H_8, m), 7.06–7.00 (H_8', m), 3.83 (H_2, s), 3.80 (H_2', s).

^{13}C NMR (126 MHz, $CDCl_3$) δ = 165.99 (C_C, s), 165.98 (C_C', s), 161.94 (C_A, s), 161.60 (C_A', s), 161.20 (C_M, d, J = 247.0 Hz), 160.54 (C_M', d, J = 246.5 Hz), 140.56 (C_I, s), 138.55 (C_I', s), 137.75 (C_J, d, J = 2.9 Hz), 135.96 (C_J', d, J = 3.2 Hz), 133.36 (C_G', s), 133.14 (C_G, s), 132.01 (C_E', s), 131.17 (C_E, s), 129.97 (C_H', s), 129.15 (C_H, s), 129.08 (C_D, s), 129.01 (C_D', s), 128.35 (C_F', s), 127.97 (C_F, s), 126.70 (C_K', d, J = 8.3 Hz), 125.90 (C_K, d, J = 8.4 Hz), 116.43 (C_L, d, J = 22.9 Hz), 115.70 (C_L', d, J = 22.7 Hz), 52.61 (C_B', s), 52.40 (C_B, s).

^{19}F{^{1}H} NMR (471 MHz, $CDCl_3$) δ = −115.04 to −115.23 (m, major conformer), −115.37 to −115.56 (m, minor conformer′).

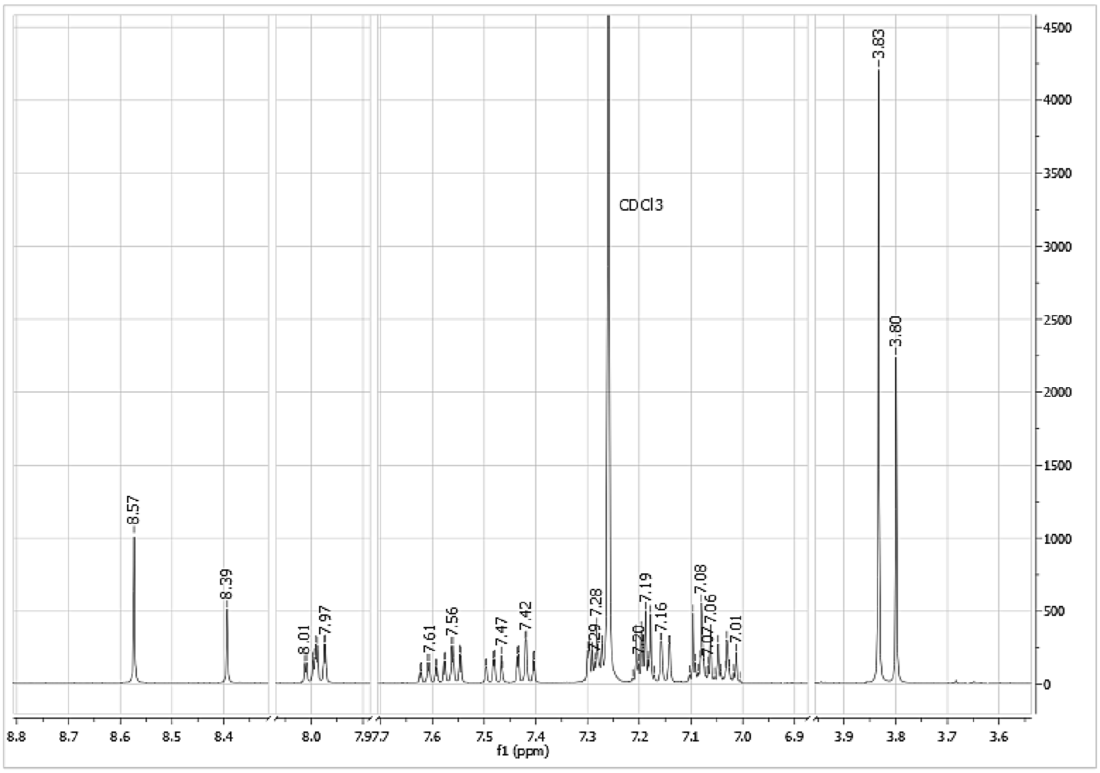

Figure S16 Partial ^{1}H NMR (500 MHz, 298 K, $CDCl_3$) of balance **1**-OMe.

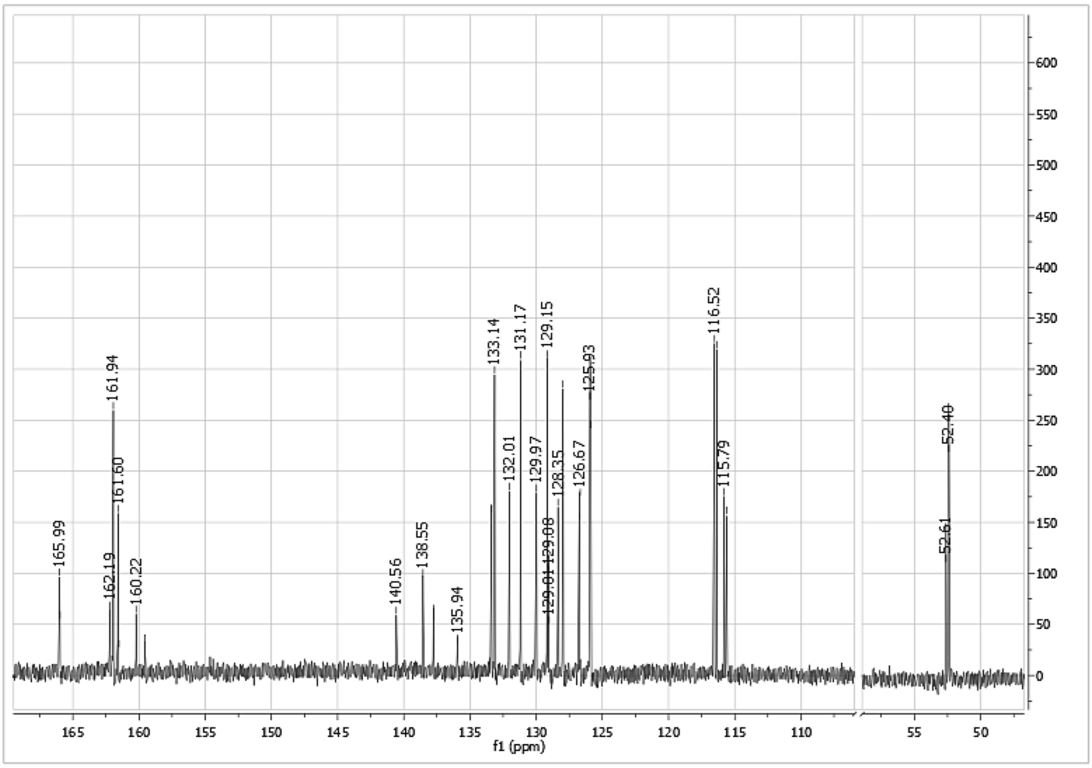

Figure S17 Partial ^{13}C NMR (126 MHz, 298 K, $CDCl_3$) of balance **1**-OMe.

N-(4-(Dimethylaminocarbonyl)phenyl)-*N*-(4-fluorophenyl)formamide **1**-NMe_2

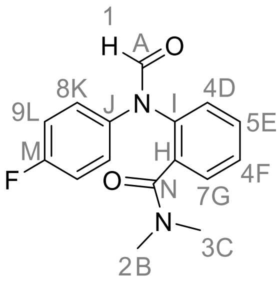

Prepared using the general procedure **a**: Formamide **S7** (0.174 g, 1.25 mmol), 2-iodo-*N,N*-dimethylbenzamide **S6** (0.400 g, 1.75 mmol), copper(I) iodide (0.119 g, 0.630 mmol), cesium fluoride (0.373 g, 2.46 mmol), DMEDA (0.160 mL, 0.150 mmol) and dry THF (2 mL). The crude product was purified by preparative TLC ($CHCl_3$/MeOH 15:1, *v*/*v*). The product was obtained as a light yellow oil (0.0370 g, 10%).

^{1}H NMR (500 MHz, $CDCl_3$) δ = 8.50 (H_1, H_1', s), 7.51 (H_5', td, J = 7.7, 1.6 Hz), 7.44–7.39 (H_6', H_5, m), 7.37 (H_4', dd, J = 3.9, 1.0 Hz), 7.36–7.33 (H_6, m), 7.34–7.29 (H_7', H_8', H_4, m), 7.28–7.23 (H_8, m), 7.14 (H_7, d, J = 8.0 Hz), 7.09–7.01 (H_9, H_9', m), 2.99 (H_2, s), 2.95 (H_3, s), 2.80 (H_2', s), 2.48 (H_3', s).

^{13}C NMR (126 MHz, $CDCl_3$) δ = 168.96 (C_N, s), 167.73 (C_N', s), 161.83 (C_A, s), 161.74 (C_A', s), 161.45 (C_M, d, J = 247.1 Hz), 160.84 (C_M', d, J = 248.7 Hz), 138.15 (C_I, s), 137.78 (C_J, d, J = 3.0 Hz), 136.99 (C_I', s), 135.18 (C_H, s), 135.07 (C_J', d, J = 3.1 Hz), 135.00 (C_H', s), 130.34 (C_E', s), 130.16 (C_E, s), 128.71 (C_D, s), 128.62 (C_D', s), 128.30 (C_G', s), 128.24 (C_F', s), 127.66 (C_K', d, J = 8.4 Hz), 127.59 (C_F, s), 127.51 (C_G, s), 126.86 (C_K, d, J = 8.5 Hz), 116.33 (C_L, d, J = 22.9 Hz), 115.62 (C_L', d, J = 22.7 Hz), 39.28 (C_C, s), 38.42 (C_C', s), 34.85 (C_B, s), 34.33 (C_B', s).

^{19}F{^{1}H} NMR (471 MHz, $CDCl_3$) δ = −114.53 to −114.65 (m, minor conformer′), −114.67 to −114.80 (m, major conformer).

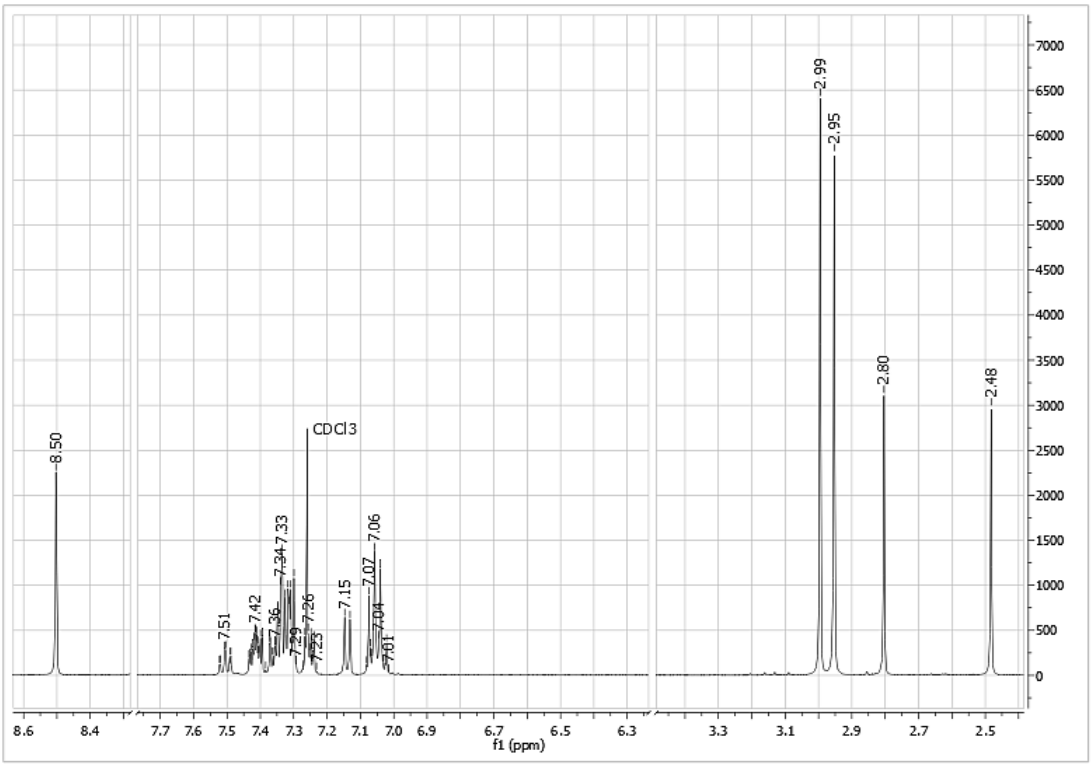

Figure S18 Partial ^{1}H NMR (500 MHz, 298 K, $CDCl_3$) of balance **1**-NMe_2.

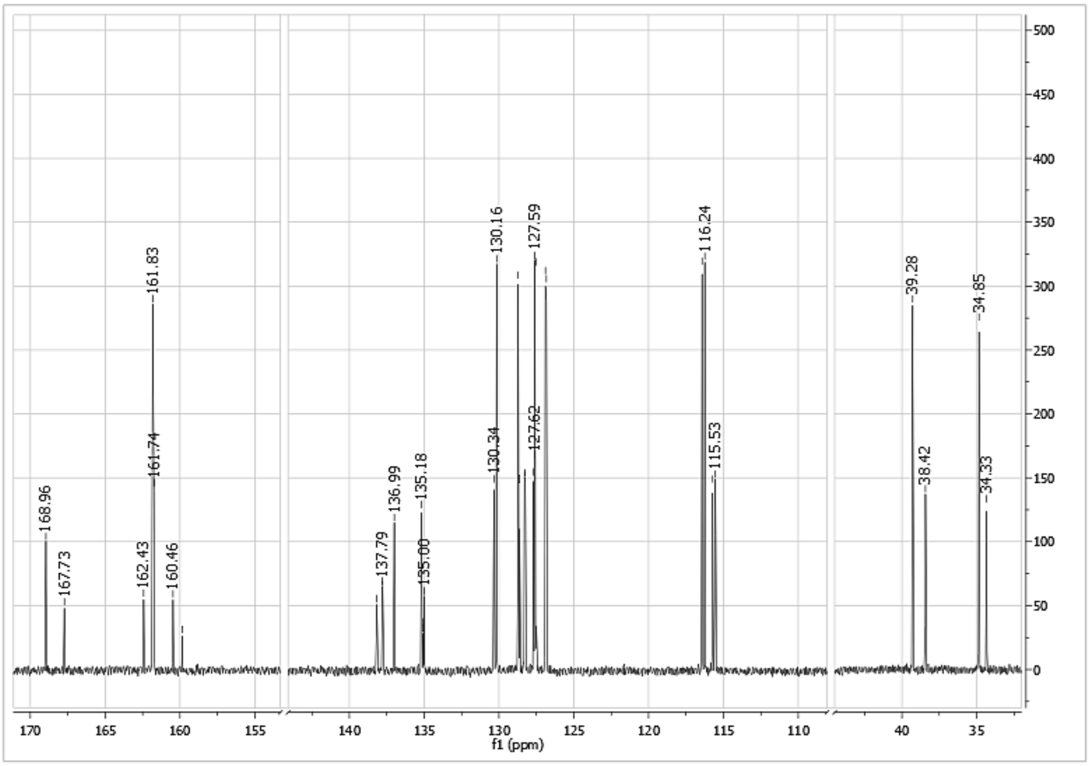

Figure S19 Partial ^{13}C NMR (126 MHz, 298 K, $CDCl_3$) of balance **1**-NMe_2.

N-(2-fluorophenyl)-4-fluorophenylamine **S8**

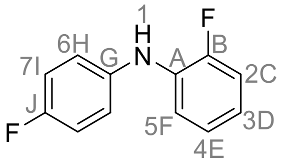

2-fluoroiodobenzene (0.325 mL, 2.70 mmol), 4-fluoroaniline (0.265 mL, 2.70 mmol), tris(dibenzylideneacetone)dipalladium(0) ($Pd_2(dba)_3$) (0.0640 g, 0.0700 mmol), bis(diphenyl-phosphino)ferrocene (0.058 g, 0.100 mmol), sodium *t*-butoxide (0.469 g, 4.18 mmol) were combined in a Schlenk flask under nitrogen. To this, dry toluene (5 mL) was added and the reaction vessel sealed. The mixture was refluxed under a nitrogen atmosphere until completion (48 h), after which a saturated aqueous solution of NH_4Cl (10 mL) was added and the product extracted with toluene (3 × 10 mL). The combined organic layers were dried over $MgSO_4$, filtered, and the toluene evaporated *in vacuo*, to afford the desired product as a brown oil (98%), which was used in the subsequent step without further purification.

^{1}H NMR (500 MHz, $CDCl_3$) δ = 7.14 (H_5, td, *J* = 8.4, 1.6 Hz, 1H), 7.12–7.05 (H_6, H_2, m, 3H), 7.04–6.97 (H_7, H_4, m, 3H), 6.85–6.78 (H_3, m, 1H), 5.69 (H_1, br s, 1H).

^{13}C NMR (126 MHz, $CDCl_3$) δ = 158.56 (C_J, d, *J* = 241.1 Hz), 152.62 (C_B, d, *J* = 240.6 Hz), 137.81 (C_G, d, *J* = 2.5 Hz), 132.57 (C_A, d, *J* = 11.0 Hz), 124.36 (C_E, d, *J* = 3.6 Hz), 121.55 (C_H, d, *J* = 7.9 Hz), 120.09 (C_D, d, *J* = 7.2 Hz), 116.10 (C_F, d, *J* = 2.3 Hz), 116.06 (C_I, d, *J* = 22.6 Hz), 115.42 (C_C, d, *J* = 19.0 Hz).

^{19}F{^{1}H} NMR (471 MHz, $CDCl_3$) δ = −120.78 to −121.03 (m), −133.32 to −133.58 (m).

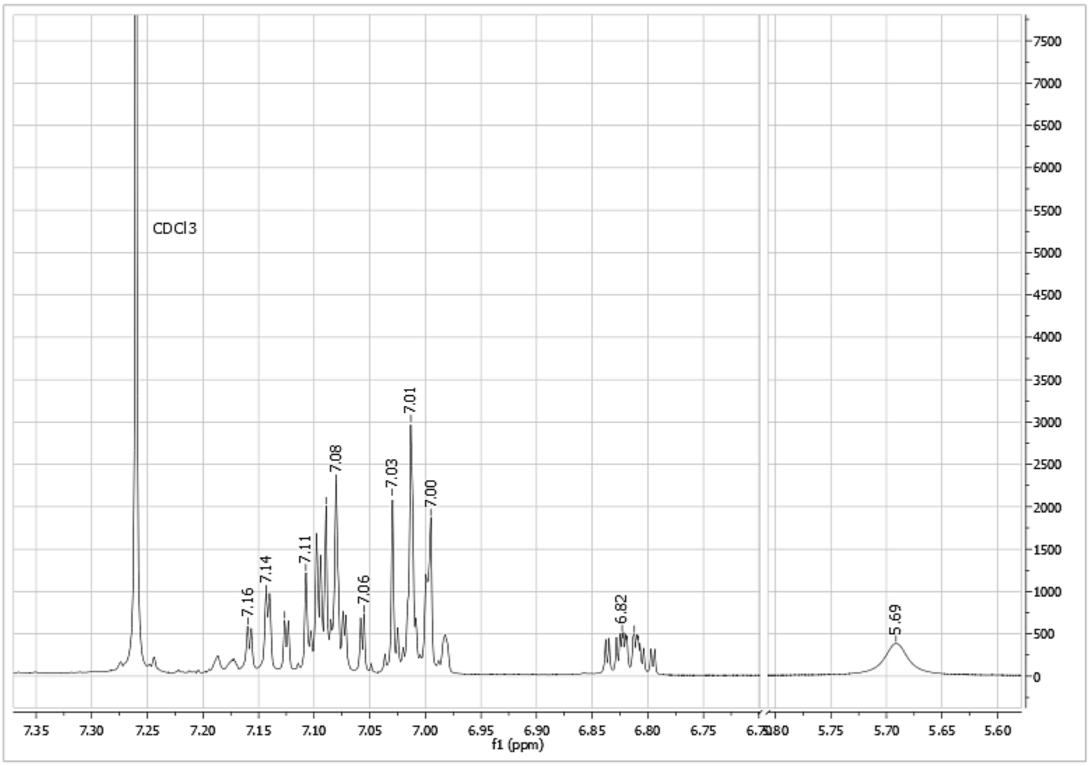

Figure S20 Partial ^{1}H NMR (500 MHz, 298 K, $CDCl_3$) of compound **S8**.

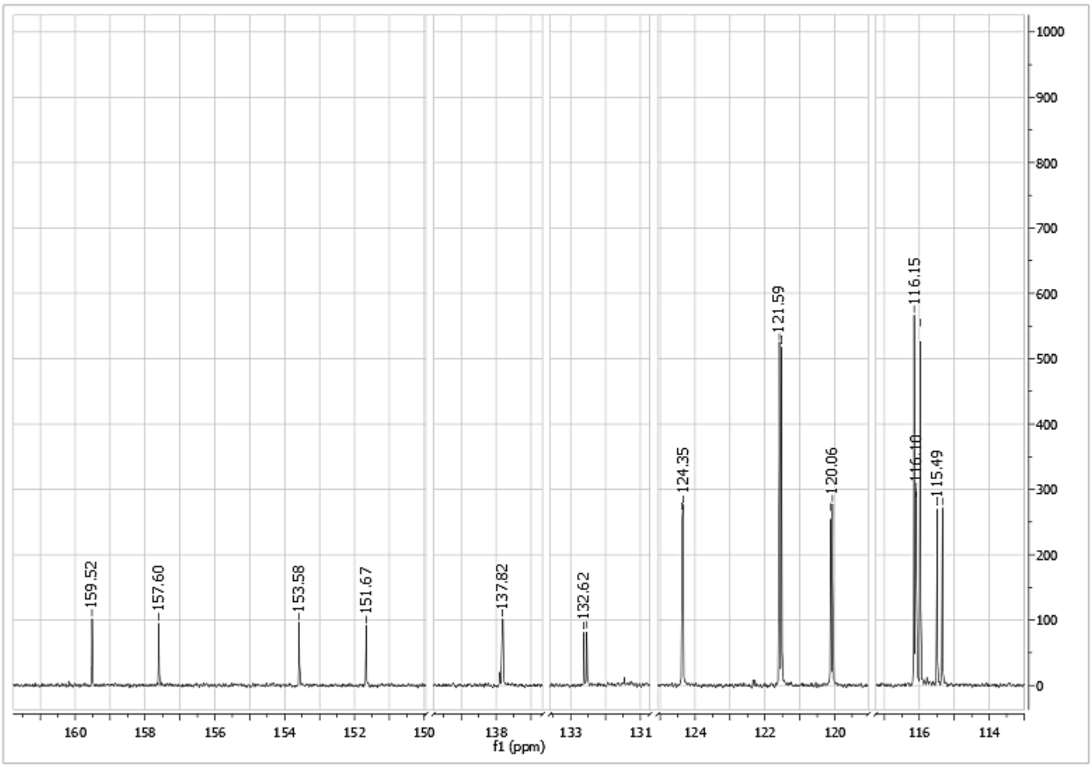

Figure S21 Partial ^{13}C NMR (126 MHz, 298 K, $CDCl_3$) of compound **S8**.

N-(2-fluorophenyl)-*N*-(4-fluorophenyl)formamide **S1**-F

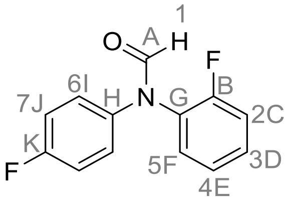

N-(2-fluorophenyl)-4-fluorophenylamine **S8** (0.560 g, 2.73 mmol) was dissolved in formic acid (8 mL) and heated to reflux for 16 h. After cooling to room temperature, CH_2Cl_2 (50 mL) was added and the reaction mixture was neutralised with saturated aqueous $NaHCO_3$ (30 mL). The organic layer was separated, washed with brine (3 × 30 mL), dried over $MgSO_4$, filtered and concentrated to yield the desired product as a brown oil (0.558 g, 88%).

^{1}H NMR (500 MHz, DMSO-d$_6$) δ = 8.71 (H_1, d, *J* = 0.9 Hz), 8.50 (H_1', d, *J* = 2.0 Hz), 7.55 (H_5', td, *J* = 8.0, 1.7 Hz), 7.51–7.43 (H_3, H_3', m), 7.44–7.36 (H_2', H_2, H_5, m), 7.36–7.28 (H_4', H_6', H_6, H_4, m), 7.28–7.22 (H_7, H_7', m).

^{13}C NMR (126 MHz, DMSO-d$_6$) δ = 162.70 (C_A', d, *J* = 1.2 Hz), 162.28 (C_A, s), 160.83 (C_K, d, *J* = 243.6 Hz), 160.53 (C_K', d, *J* = 243.8 Hz), 157.86 (C_B, d, *J* = 249.5 Hz), 157.77 (C_B', d, *J* = 248.5 Hz), 137.88 (C_H, d, *J* = 2.7 Hz), 136.26 (C_H', d, *J* = 2.8 Hz), 130.66 (C_F, s), 130.61 (C_D', d, *J* = 8.2 Hz), 130.59 (C_D, d, *J* = 8.0 Hz), 130.31 (C_F', s), 128.94 (C_G', d, *J* = 11.7 Hz), 127.69 (C_I', d, *J* = 8.6 Hz), 127.20 (C_G, d, *J* = 12.8 Hz), 126.07 (C_E', d, *J* = 3.8 Hz), 125.91 (C_I, d, *J* = 8.7 Hz), 125.79 (C_E, d, *J* = 3.6 Hz), 117.42 (C_C', d, *J* = 19.8 Hz), 117.13 (C_C, d, *J* = 19.6 Hz), 116.88 (C_J, d, *J* = 22.9 Hz), 116.32 (C_J', d, *J* = 22.7 Hz).

^{19}F{^{1}H} NMR (471 MHz, DMSO-d$_6$) δ = −115.30 to −115.57 (m), −115.87 to −116.16 (m), −120.37 to −120.73 (m), −123.00 to −123.35 (m).

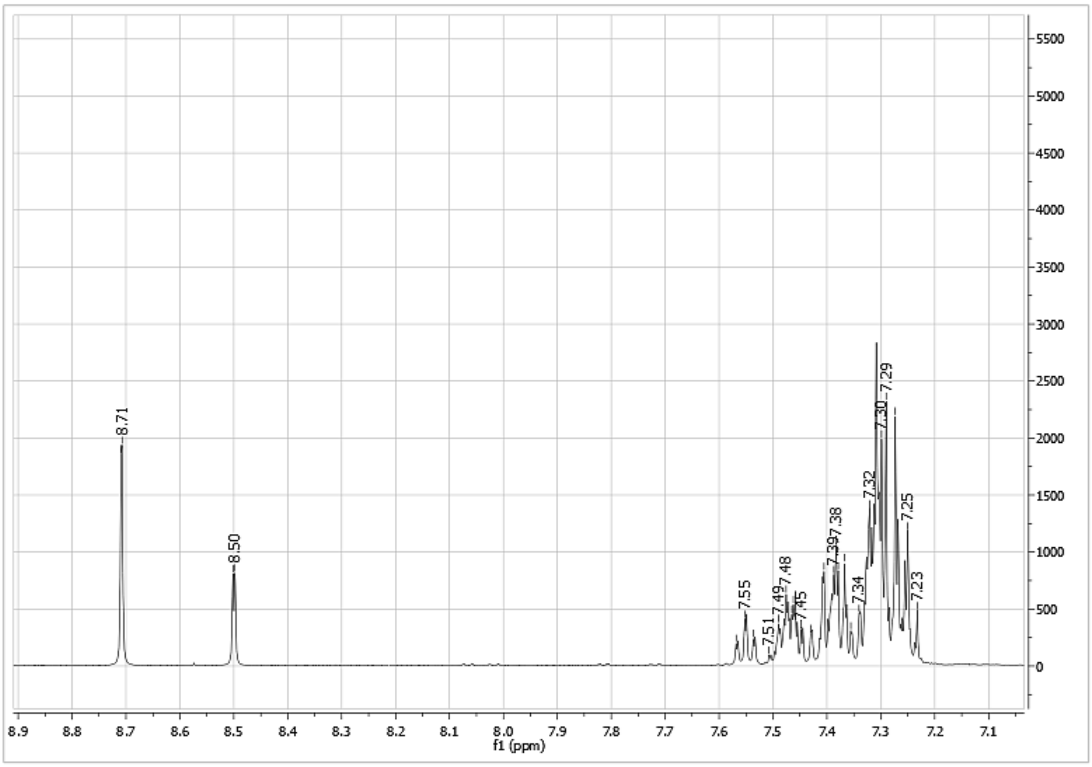

Figure S22 Partial ^{1}H NMR (500 MHz, 298 K, DMSO-d_6) of balance **S1**.

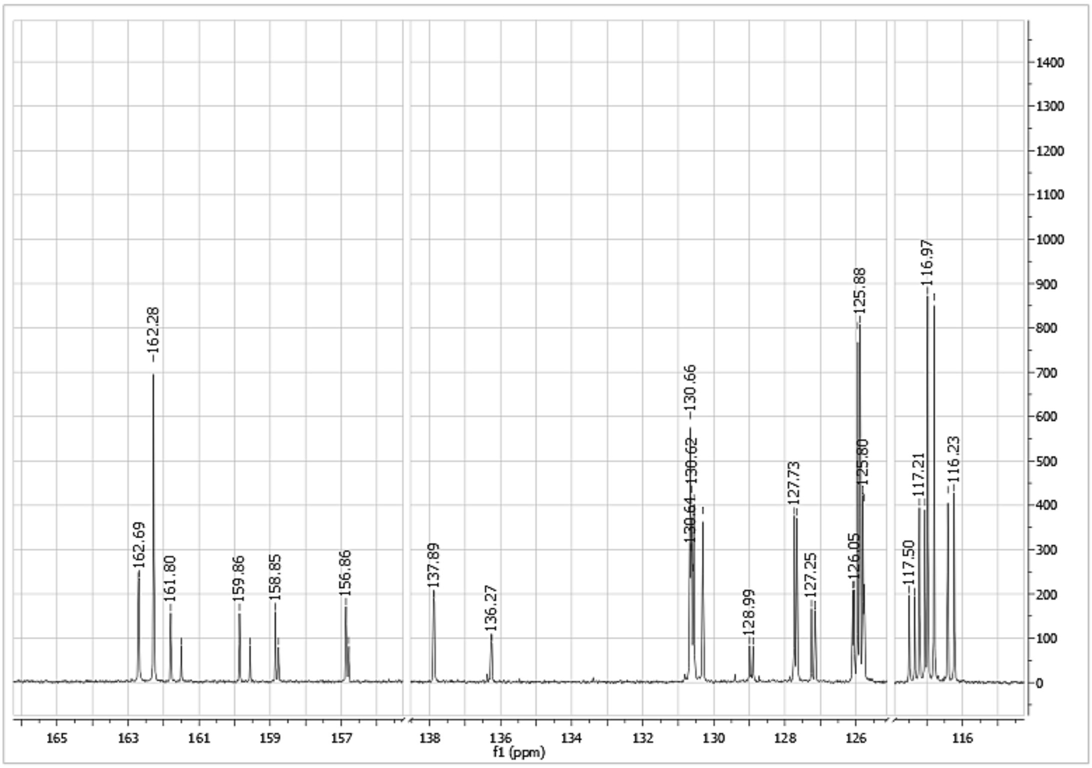

Figure S23 Partial ^{13}C NMR (126 MHz, 298 K, DMSO-d_6) of balance **S1**.

S2.3 Conformer assignment using NMR spectroscopy

Conformer assignment for balance series **1** and control balances **S1**, **S2** and **S3** was performed by a combination of NMR spectroscopy techniques (^{1}H, ^{13}C, COSY, ^{1}H-^{13}C HSQC, ^{1}H-^{13}C HMBC and ^{1}H NOESY). The main characteristics for distinguishing between the two conformers were:

a) ^{1}H-^{13}C long-range through-bond correlation between the major formyl proton and the major quaternary carbon in the X-substituted ring, and between the minor formyl proton and the minor quaternary carbon in the F-substituted ring (marked in blue in **Figure S24**), resulting in respective cross-peaks on the ^{1}H-^{13}C HMBC spectrum that allowed for an unambiguous determination of the major and minor conformer.

b) Nuclear Overhauser Effect resulting in the through-space coupling between the major formyl proton and the major proton on the F-substituted aromatic ring, and between the minor formyl proton and the minor aromatic proton on the X-substituted ring (marked in pink in **Figure S24**), resulting in respective cross peaks on the NOESY spectrum. These were treated as a confirmation of the assignment from a), as occasionally the minor cross-peak was too weak to be seen, or multiple cross-peaks would be visible due to the NOESY experiment timescale being comparable to the conformer interconversion timescale.

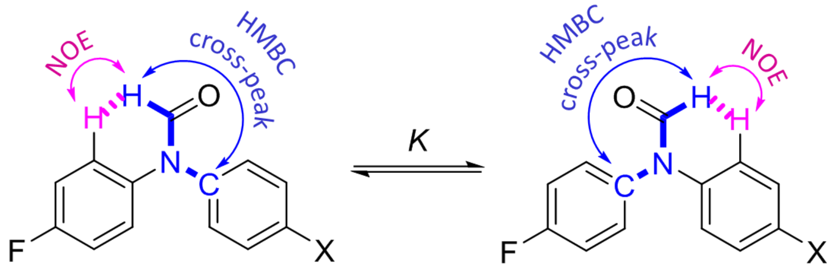

“O” conformer “H” conformer

Figure S24 ^{1}H-^{1}H and ^{1}H-^{13}C couplings used for “O” and “H” conformer assignment (closed and open conformers respectively).

S2.4 Crystal Structures of 1-H and 1-Me

Balances **1**-H and **1**-Me were crystallised from an Et_2O/*n*-hexane solvent system by slow vapour diffusion technique, and the structures determined by X-ray diffraction are shown in **Figure S25** and **Figure S26**.

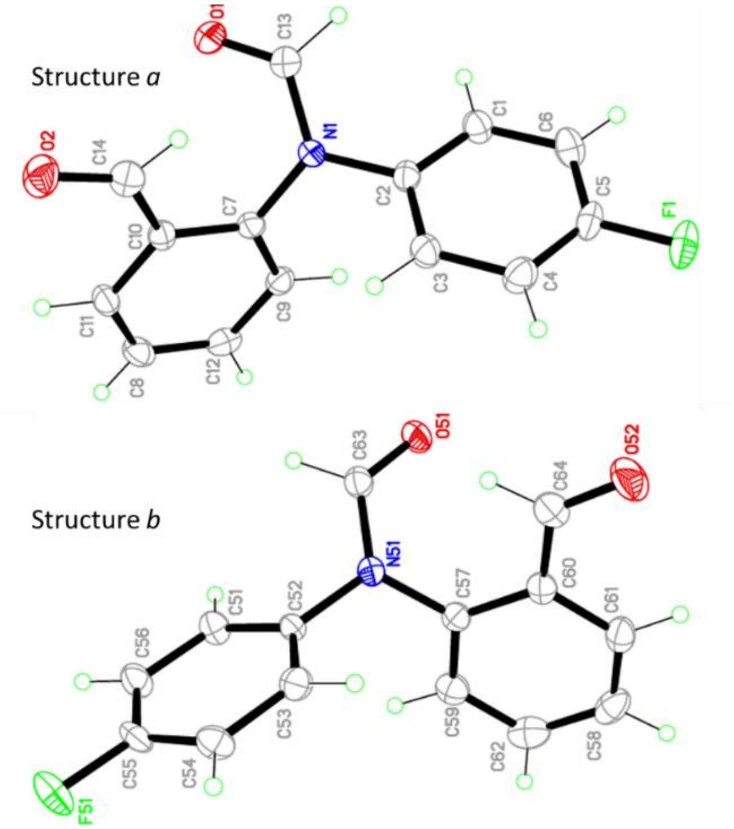

Figure S25 The asymmetric unit of balance **1**-H, with displacement ellipsoids at the 50% probability level.

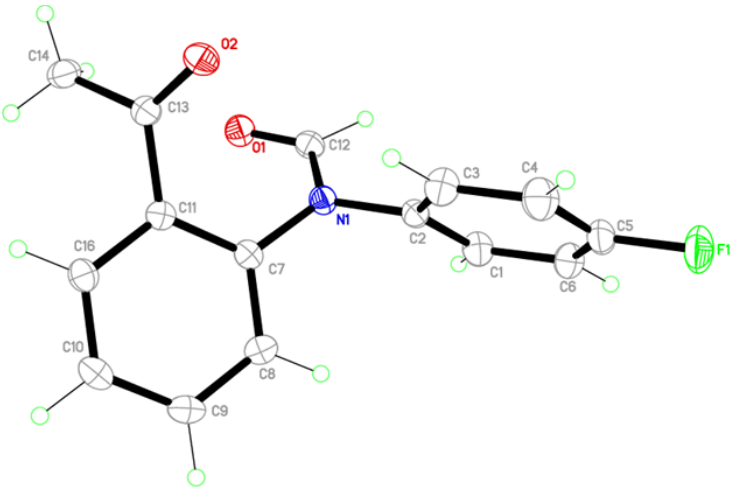

Figure S26 The asymmetric unit of balance **1**-Me, with displacement ellipsoids at the 50% probability level.

Balance **1**-H crystallised as an asymmetric unit containing two distinct polymorphs ***a*** and ***b*** (**Figure S25**). The carbonyl groups of these structures seem to display a sheared parallel orientation. The measured distances between the aldehyde carbon and the formyl oxygen are longer (2.930 Å in structure *a* and 3.035 Å in structure *b*, **Figure S27A**) are shorter than the sum of van der Waals radii of carbon and oxygen (3.22 Å). The interaction angles are larger (123.43° and 131.67°, Figure 29a), than that seen for a typical of a Bürgi-Dunitz trajectory (100–110°). There is also no significant pyramidalisation of any of the carbonyl carbons.

Balance **1**-Me crystallised as the "O" (closed) conformer (**Figure S26**), with a distance of 2.784 Å between the acetyl carbon and the formyl oxygen (**Figure S27B**, **C**), shorter than the sum of van der Waals radii of carbon and oxygen (3.22 Å). The shortest contact, however, arises between the acetyl α proton (slightly acidic) and the formyl oxygen (2.599 Å). This may constitute an additional attractive interaction stabilising the "O" (closed) conformer of **1**-Me. No significant pyramidalisation of any of the carbonyl carbons was seen (**Figure S29**).

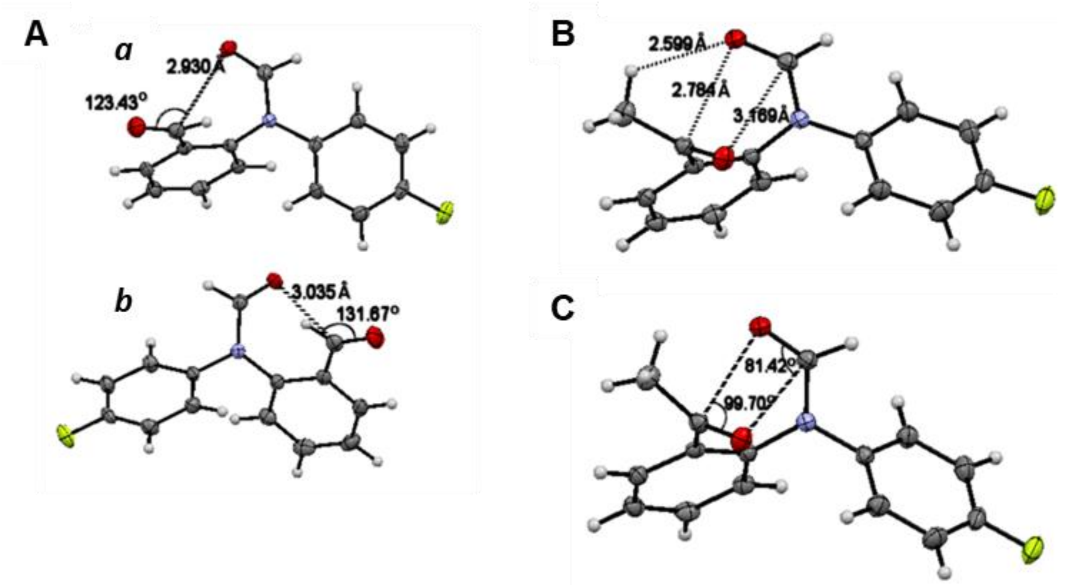

Figure S27 Interaction distances and angles in **A** balance **1**-H (polymorphs ***a*** and ***b***) and **B**, **C** balance **1**-Me.

The extended crystal structure reveals the presence of weak and long intermolecular CH–O hydrogen bonds in the solid state of **1**-Me (**Figure S28**).

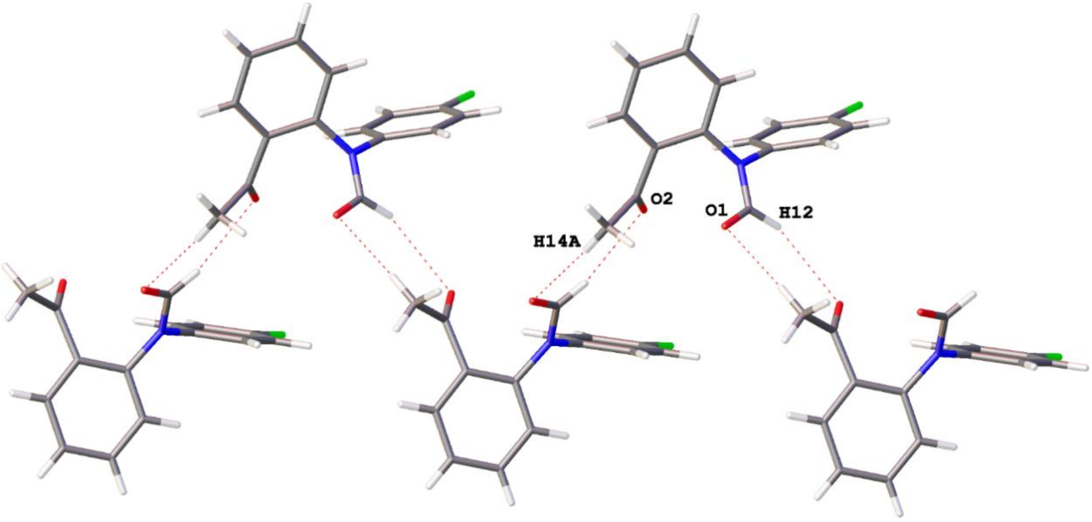

Figure S28 Intermolecular interactions involving the carbonyl groups in the extended crystal packing of balance **1**-Me (dashed lines).

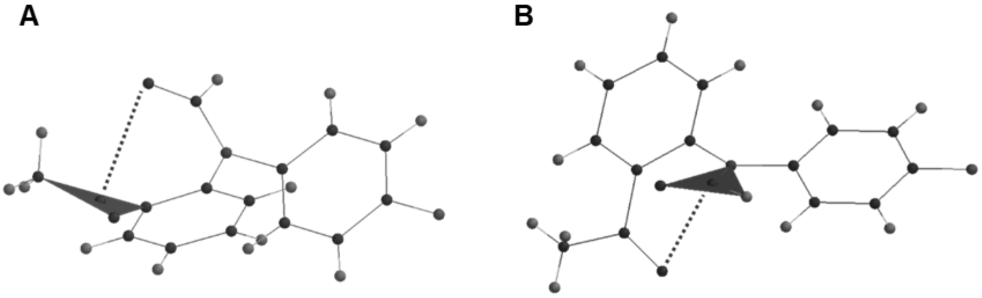

Figure S29 Example of pyramidalisation measurements on balance **1**-Me for **A** the *ortho*-substituent carbonyl carbon and **B** the formamide carbonyl carbon.

Table S1 Selected distances and angles of the C=O···C=O interactions determined from the crystal structures of **1**-H (polymorphs ***a*** and **b**) and **1**-Me.

**Balance**	**1**-H ***a***	**1**-H ***b***	**1**-Me	
C=O···C=O distance, Å	2.930(3)	3.035(3)	2.784	3.169
Angle of contact, °	123.43	131.67	99.70	81.42
Formamide carbonyl C pyramidalisation Δ, Å	0.005(7)	0.009(6)	0.0003	
Substituent carbonyl C pyramidalisation Δ, Å	0.004(7)	0.003(6)	0.0051	

Full Crystallographic data for balance 1-H

Table S2 Crystal data and structure refinement for balance **1**-H.

**Identification code**	sc4007	
**Empirical formula**	$C_{14} H_{10} F N O_2$	
**Formula weight**	243.23	
**Temperature**	120.0 K	
**Wavelength**	0.71073 Å	
**Crystal system**	Monoclinic	
**Space group**	P 1 21/c 1	
**Unit cell dimensions**	a = 18.1318(19) Å	α = 90°.
	b = 7.8169(6) Å	β = 114.155(12)°.
	c = 17.8670(16) Å	γ = 90°.
**Volume**	2310.7(4) $Å^3$	
**Z**	8	
**Density (calculated)**	1.398 Mg/m^3	
**Absorption coefficient**	0.105 mm^{-1}	
**F(000)**	1008	
**Crystal size**	0.191 × 0.1532 × 0.1009 mm3	
**Theta range for data collection**	2.882 to 26.371°.	
**Index ranges**	−22<=h<=22, −9<=k<=9, −21<=l<=22	
**Reflections collected**	19448	
**Independent reflections**	4711 [R(int) = 0.0579]	
**Completeness to theta = 26.000°**	99.8%	
**Absorption correction**	Gaussian	
**Max. and min. transmission**	0.999 and 0.998	
**Refinement method**	Full-matrix least-squares on F2	
**Data / restraints / parameters**	4711 / 0 / 406	
**Goodness-of-fit on F2**	1.069	
**Final R indices [I>2sigma(I)]**	R1 = 0.0529, wR2 = 0.0965	
**R indices (all data)**	R1 = 0.0773, wR2 = 0.1058	
**Extinction coefficient**	0.0021(4)	
**Largest diff. peak and hole**	0.232 and -0.236 e.$Å^{-3}$	

Table S3 Atomic coordinates (× 10^4) and equivalent isotropic displacement parameters ($Å^2$ × 10^3) for balance **1**-H. U(eq) is defined as one third of the trace of the orthogonalised U^{ij} tensor.

	x	y	z	U(eq)
**F(1)**	5823(1)	6490(2)	6272(1)	34(1)
**O(1)**	2723(1)	10400(2)	2413(1)	33(1)
**O(2)**	1017(1)	10912(2)	3062(1)	31(1)
**N(1)**	3187(1)	8570(2)	3506(1)	20(1)
**C(1)**	4639(1)	7922(3)	4202(1)	22(1)
**C(2)**	3881(1)	8014(3)	4221(1)	19(1)
**C(3)**	3777(1)	7572(3)	4921(1)	24(1)
**C(4)**	4434(1)	7043(3)	5612(1)	26(1)
**C(5)**	5176(1)	7000(3)	5582(1)	22(1)
**C(6)**	5301(1)	7431(3)	4901(1)	27(1)
**C(7)**	2458(1)	7564(3)	3221(1)	18(1)
**C(8)**	1059(1)	5604(3)	2578(1)	26(1)
**C(9)**	2510(1)	5830(3)	3096(1)	21(1)
**C(10)**	1709(1)	8328(3)	3054(1)	18(1)
**C(11)**	1012(1)	7331(3)	2724(1)	23(1)
**C(12)**	1810(1)	4860(3)	2776(1)	25(1)
**C(13)**	3259(1)	9912(3)	3054(1)	27(1)
**C(14)**	1652(1)	10131(3)	3269(1)	24(1)
**F(51)**	−1069(1)	3206(2)	5300(1)	32(1)
**O(51)**	2772(1)	3137(2)	4627(1)	27(1)
**O(52)**	4305(1)	3903(2)	6924(1)	38(1)
**N(51)**	1976(1)	2533(2)	5307(1)	19(1)
**C(51)**	494(1)	2364(3)	4582(1)	22(1)
**C(52)**	1178(1)	2669(3)	5298(1)	18(1)
**C(53)**	1104(1)	3120(3)	6016(1)	23(1)
**C(54)**	343(1)	3273(3)	6020(1)	24(1)
**C(55)**	−321(1)	2998(3)	5298(1)	22(1)
**C(56)**	−269(1)	2548(3)	4578(1)	25(1)
**C(57)**	2525(1)	1295(3)	5847(1)	18(1)
**C(58)**	3541(2)	−1226(3)	6836(1)	30(1)
**C(59)**	2251(1)	−373(3)	5817(1)	24(1)
**C(60)**	3317(1)	1719(3)	6383(1)	20(1)

**C(61)**	3819(1)	430(3)	6869(1)	26(1)
**C(62)**	2754(2)	−1625(3)	6316(1)	31(1)
**C(63)**	2146(1)	3327(3)	4715(1)	21(1)
**C(64)**	3617(1)	3500(3)	6477(1)	25(1)

Table S4 Bond lengths [Å] and angles [°] for balance **1**-H.

**F(1)-C(5)**	1.367(2)
**O(1)-C(13)**	1.222(3)
**O(2)-C(14)**	1.220(3)
**N(1)-C(2)**	1.445(2)
**N(1)-C(7)**	1.439(3)
**N(1)-C(13)**	1.363(3)
**C(1)-H(1)**	1.00(2)
**C(1)-C(2)**	1.390(3)
**C(1)-C(6)**	1.386(3)
**C(2)-C(3)**	1.383(3)
**C(3)-H(3)**	0.94(2)
**C(3)-C(4)**	1.383(3)
**C(4)-H(4)**	0.96(2)
**C(4)-C(5)**	1.369(3)
**C(5)-C(6)**	1.369(3)
**C(6)-H(6)**	0.94(2)
**C(7)-C(9)**	1.384(3)
**C(7)-C(10)**	1.400(3)
**C(8)-H(8)**	0.97(2)
**C(8)-C(11)**	1.384(3)
**C(8)-C(12)**	1.386(3)
**C(9)-H(9)**	0.98(2)
**C(9)-C(12)**	1.386(3)
**C(10)-C(11)**	1.394(3)
**C(10)-C(14)**	1.475(3)
**C(11)-H(11)**	0.98(2)
**C(12)-H(12)**	0.96(2)
**C(13)-H(13)**	1.03(2)

**C(14)-H(14)**	1.01(3)
**F(51)-C(55)**	1.368(2)
**O(51)-C(63)**	1.218(2)
**O(52)-C(64)**	1.215(3)
**N(51)-C(52)**	1.443(2)
**N(51)-C(57)**	1.440(3)
**N(51)-C(63)**	1.367(3)
**C(51)-H(51)**	0.96(2)
**C(51)-C(52)**	1.391(3)
**C(51)-C(56)**	1.387(3)
**C(52)-C(53)**	1.388(3)
**C(53)-H(53)**	0.97(2)
**C(53)-C(54)**	1.388(3)
**C(54)-H(54)**	0.94(2)
**C(54)-C(55)**	1.374(3)
**C(55)-C(56)**	1.373(3)
**C(56)-H(56)**	0.97(2)
**C(57)-C(59)**	1.389(3)
**C(57)-C(60)**	1.400(3)
**C(58)-H(58)**	0.96(2)
**C(58)-C(61)**	1.381(3)
**C(58)-C(62)**	1.382(3)
**C(59)-H(59)**	0.97(2)
**C(59)-C(62)**	1.385(3)
**C(60)-C(61)**	1.397(3)
**C(60)-C(64)**	1.479(3)
**C(61)-H(61)**	0.95(2)
**C(62)-H(62)**	0.98(3)
**C(63)-H(63)**	1.02(2)
**C(64)-H(64)**	1.02(2)
**C(7)-N(1)-C(2)**	119.35(16)
**C(13)-N(1)-C(2)**	119.61(17)
**C(13)-N(1)-C(7)**	120.54(17)
**C(2)-C(1)-H(1)**	120.9(12)
**C(6)-C(1)-H(1)**	119.5(12)

**C(6)-C(1)-C(2)**	119.5(2)
**C(1)-C(2)-N(1)**	120.45(18)
**C(3)-C(2)-N(1)**	118.79(18)
**C(3)-C(2)-C(1)**	120.76(19)
**C(2)-C(3)-H(3)**	120.3(14)
**C(4)-C(3)-C(2)**	119.7(2)
**C(4)-C(3)-H(3)**	120.0(14)
**C(3)-C(4)-H(4)**	121.8(13)
**C(5)-C(4)-C(3)**	118.3(2)
**C(5)-C(4)-H(4)**	119.9(13)
**F(1)-C(5)-C(4)**	117.86(19)
**F(1)-C(5)-C(6)**	118.66(19)
**C(6)-C(5)-C(4)**	123.5(2)
**C(1)-C(6)-H(6)**	120.5(14)
**C(5)-C(6)-C(1)**	118.1(2)
**C(5)-C(6)-H(6)**	121.3(14)
**C(9)-C(7)-N(1)**	118.64(19)
**C(9)-C(7)-C(10)**	120.5(2)
**C(10)-C(7)-N(1)**	120.82(19)
**C(11)-C(8)-H(8)**	120.7(14)
**C(11)-C(8)-C(12)**	119.4(2)
**C(12)-C(8)-H(8)**	119.9(14)
**C(7)-C(9)-H(9)**	121.0(13)
**C(7)-C(9)-C(12)**	119.4(2)
**C(12)-C(9)-H(9)**	119.5(13)
**C(7)-C(10)-C(14)**	121.13(19)
**C(11)-C(10)-C(7)**	118.9(2)
**C(11)-C(10)-C(14)**	119.82(19)
**C(8)-C(11)-C(10)**	120.7(2)
**C(8)-C(11)-H(11)**	120.6(13)
**C(10)-C(11)-H(11)**	118.6(13)
**C(8)-C(12)-H(12)**	119.8(13)
**C(9)-C(12)-C(8)**	120.9(2)
**C(9)-C(12)-H(12)**	119.3(13)
**O(1)-C(13)-N(1)**	124.3(2)

**O(1)-C(13)-H(13)**	123.3(13)
**N(1)-C(13)-H(13)**	112.4(13)
**O(2)-C(14)-C(10)**	123.8(2)
**O(2)-C(14)-H(14)**	120.7(14)
**C(10)-C(14)-H(14)**	115.4(14)
**C(57)-N(51)-C(52)**	118.18(16)
**C(63)-N(51)-C(52)**	120.02(17)
**C(63)-N(51)-C(57)**	120.72(17)
**C(52)-C(51)-H(51)**	120.7(12)
**C(56)-C(51)-H(51)**	119.2(12)
**C(56)-C(51)-C(52)**	120.0(2)
**C(51)-C(52)-N(51)**	120.71(17)
**C(53)-C(52)-N(51)**	118.93(18)
**C(53)-C(52)-C(51)**	120.36(19)
**C(52)-C(53)-H(53)**	120.2(13)
**C(54)-C(53)-C(52)**	119.9(2)
**C(54)-C(53)-H(53)**	119.9(12)
**C(53)-C(54)-H(54)**	121.6(13)
**C(55)-C(54)-C(53)**	118.2(2)
**C(55)-C(54)-H(54)**	120.2(13)
**F(51)-C(55)-C(54)**	117.90(18)
**F(51)-C(55)-C(56)**	118.75(19)
**C(56)-C(55)-C(54)**	123.3(2)
**C(51)-C(56)-H(56)**	123.1(13)
**C(55)-C(56)-C(51)**	118.1(2)
**C(55)-C(56)-H(56)**	118.8(13)
**C(59)-C(57)-N(51)**	117.80(18)
**C(59)-C(57)-C(60)**	119.97(19)
**C(60)-C(57)-N(51)**	122.23(18)
**C(61)-C(58)-H(58)**	117.0(15)
**C(61)-C(58)-C(62)**	119.9(2)
**C(62)-C(58)-H(58)**	123.1(15)
**C(57)-C(59)-H(59)**	117.7(14)
**C(62)-C(59)-C(57)**	120.5(2)
**C(62)-C(59)-H(59)**	121.8(14)

**C(57)-C(60)-C(64)**	121.85(19)
**C(61)-C(60)-C(57)**	118.6(2)
**C(61)-C(60)-C(64)**	119.4(2)
**C(58)-C(61)-C(60)**	121.0(2)
**C(58)-C(61)-H(61)**	121.3(14)
**C(60)-C(61)-H(61)**	117.7(14)
**C(58)-C(62)-C(59)**	120.0(2)
**C(58)-C(62)-H(62)**	119.9(14)
**C(59)-C(62)-H(62)**	120.2(14)
**O(51)-C(63)-N(51)**	124.2(2)
**O(51)-C(63)-H(63)**	123.6(11)
**N(51)-C(63)-H(63)**	112.1(11)
**O(52)-C(64)-C(60)**	123.4(2)
**O(52)-C(64)-H(64)**	120.7(13)
**C(60)-C(64)-H(64)**	115.9(13)

Table S5 Anisotropic displacement parameters ($Å^2 \times 10^3$) for balance **1**-H. The anisotropic displacement factor exponent takes the form: $-2\pi^2[h^2 a^{*2}U^{11} + ... + 2 h k a^* b^* U^{12}]$

	U^{11}	U^{22}	U^{33}	U^{23}	U^{13}	U^{12}
**F(1)**	26(1)	37(1)	25(1)	3(1)	-2(1)	9(1)
**O(1)**	24(1)	37(1)	31(1)	17(1)	4(1)	-1(1)
**O(2)**	31(1)	32(1)	36(1)	4(1)	18(1)	11(1)
**N(1)**	15(1)	21(1)	21(1)	4(1)	6(1)	−2(1)
**C(1)**	19(1)	27(1)	22(1)	−1(1)	9(1)	0(1)
**C(2)**	17(1)	18(1)	18(1)	0(1)	5(1)	1(1)
**C(3)**	18(1)	30(1)	26(1)	4(1)	11(1)	4(1)
**C(4)**	28(1)	30(1)	21(1)	4(1)	9(1)	3(1)
**C(5)**	21(1)	19(1)	19(1)	−1(1)	0(1)	6(1)
**C(6)**	18(1)	33(1)	28(1)	−4(1)	9(1)	3(1)
**C(7)**	18(1)	21(1)	16(1)	2(1)	7(1)	−1(1)
**C(8)**	21(1)	31(1)	23(1)	0(1)	6(1)	−9(1)
**C(9)**	22(1)	21(1)	19(1)	4(1)	9(1)	3(1)
**C(10)**	18(1)	22(1)	15(1)	1(1)	7(1)	1(1)
**C(11)**	15(1)	32(1)	22(1)	4(1)	8(1)	1(1)

**C(12)**	33(1)	20(1)	22(1)	2(1)	11(1)	-2(1)
**C(13)**	22(1)	26(1)	29(1)	6(1)	6(1)	-1(1)
**C(14)**	26(1)	28(1)	21(1)	−1(1)	12(1)	2(1)
**F(51)**	20(1)	38(1)	42(1)	−11(1)	18(1)	-4(1)
**O(51)**	18(1)	38(1)	28(1)	6(1)	13(1)	2(1)
**O(52)**	25(1)	50(1)	32(1)	−5(1)	5(1)	−14(1)
**N(51)**	16(1)	23(1)	19(1)	2(1)	7(1)	1(1)
**C(51)**	20(1)	26(1)	21(1)	−4(1)	9(1)	0(1)
**C(52)**	15(1)	18(1)	22(1)	3(1)	9(1)	1(1)
**C(53)**	22(1)	27(1)	19(1)	0(1)	6(1)	−2(1)
**C(54)**	26(1)	28(1)	22(1)	−4(1)	14(1)	−3(1)
**C(55)**	16(1)	23(1)	32(1)	−1(1)	14(1)	−3(1)
**C(56)**	18(1)	32(1)	23(1)	−3(1)	7(1)	−4(1)
**C(57)**	18(1)	20(1)	19(1)	0(1)	10(1)	3(1)
**C(58)**	36(2)	32(1)	27(1)	9(1)	18(1)	16(1)
**C(59)**	25(1)	23(1)	27(1)	−2(1)	13(1)	0(1)
**C(60)**	19(1)	27(1)	17(1)	−2(1)	10(1)	2(1)
**C(61)**	20(1)	40(2)	19(1)	2(1)	9(1)	7(1)
**C(62)**	43(2)	21(1)	36(1)	1(1)	25(1)	3(1)
**C(63)**	18(1)	24(1)	21(1)	0(1)	6(1)	0(1)
**C(64)**	24(1)	32(1)	20(1)	−5(1)	10(1)	−4(1)

Table S6 Hydrogen coordinates (× 10^4) and isotropic displacement parameters (Å^2 × 10^3) for balance **1**-H.

	**x**	**y**	**z**	**U(eq)**
**H(1)**	4715(12)	8220(30)	3694(12)	21(5)
**H(3)**	3262(15)	7660(30)	4932(13)	34(6)
**H(4)**	4384(13)	6760(30)	6114(13)	31(6)
**H(6)**	5816(15)	7370(30)	4898(13)	36(7)
**H(8)**	575(14)	4920(30)	2334(13)	31(6)
**H(9)**	3036(13)	5270(30)	3242(12)	23(6)
**H(11)**	490(13)	7870(30)	2604(12)	23(6)
**H(12)**	1848(12)	3650(30)	2700(12)	18(5)
**H(13)**	3827(15)	10460(30)	3308(14)	40(7)
**H(14)**	2184(15)	10700(30)	3615(14)	41(7)

**H(51)**	541(12)	2000(30)	4090(12)	22(6)
**H(53)**	1583(13)	3350(30)	6513(13)	25(6)
**H(54)**	276(12)	3580(30)	6499(13)	23(6)
**H(56)**	−766(14)	2370(30)	4091(13)	28(6)
**H(58)**	3920(15)	−2060(30)	7176(14)	38(7)
**H(59)**	1699(14)	−620(30)	5444(13)	29(6)
**H(61)**	4355(14)	740(30)	7227(13)	27(6)
**H(62)**	2553(14)	−2790(30)	6304(13)	36(7)
**H(63)**	1677(13)	4070(30)	4340(12)	20(5)
**H(64)**	3207(14)	4400(30)	6146(13)	31(6)

Table S7 Torsion angles [°] for balance **1**-H.

**F(1)-C(5)-C(6)-C(1)**	179.1(2)
**N(1)-C(2)-C(3)-C(4)**	−179.7(2)
**N(1)-C(7)-C(9)-C(12)**	−176.17(16)
**N(1)-C(7)-C(10)-C(11)**	175.46(16)
**N(1)-C(7)-C(10)-C(14)**	−8.4(3)
**C(1)-C(2)-C(3)-C(4)**	0.5(3)
**C(2)-N(1)-C(7)-C(9)**	−51.2(2)
**C(2)-N(1)-C(7)-C(10)**	130.34(19)
**C(2)-N(1)-C(13)-O(1)**	175.0(2)
**C(2)-C(1)-C(6)-C(5)**	2.0(3)
**C(2)-C(3)-C(4)-C(5)**	1.0(3)
**C(3)-C(4)-C(5)-F(1)**	179.42(19)
**C(3)-C(4)-C(5)-C(6)**	−0.9(4)
**C(4)-C(5)-C(6)-C(1)**	−0.6(4)
**C(6)-C(1)-C(2)-N(1)**	178.2(2)
**C(6)-C(1)-C(2)-C(3)**	−2.0(3)
**C(7)-N(1)-C(2)-C(1)**	130.5(2)
**C(7)-N(1)-C(2)-C(3)**	−49.3(3)
**C(7)-N(1)-C(13)-O(1)**	3.1(3)
**C(7)-C(9)-C(12)-C(8)**	0.1(3)
**C(7)-C(10)-C(11)-C(8)**	1.3(3)
**C(7)-C(10)-C(14)-O(2)**	171.98(19)

**C(9)-C(7)-C(10)-C(11)**	−3.0(3)
**C(9)-C(7)-C(10)-C(14)**	173.17(18)
**C(10)-C(7)-C(9)-C(12)**	2.3(3)
**C(11)-C(8)-C(12)-C(9)**	−1.8(3)
**C(11)-C(10)-C(14)-O(2)**	−11.9(3)
**C(12)-C(8)-C(11)-C(10)**	1.0(3)
**C(13)-N(1)-C(2)-C(1)**	−41.5(3)
**C(13)-N(1)-C(2)-C(3)**	138.7(2)
**C(13)-N(1)-C(7)-C(9)**	120.7(2)
**C(13)-N(1)-C(7)-C(10)**	−57.8(3)
**C(14)-C(10)-C(11)-C(8)**	−174.90(19)
**F(51)-C(55)-C(56)-C(51)**	179.1(2)
**N(51)-C(52)-C(53)-C(54)**	179.5(2)
**N(51)-C(57)-C(59)-C(62)**	179.36(18)
**N(51)-C(57)-C(60)-C(61)**	−178.07(17)
**N(51)-C(57)-C(60)-C(64)**	5.2(3)
**C(51)-C(52)-C(53)-C(54)**	−0.2(3)
**C(52)-N(51)-C(57)-C(59)**	50.9(2)
**C(52)-N(51)-C(57)-C(60)**	−130.09(19)
**C(52)-N(51)-C(63)-O(51)**	−172.2(2)
**C(52)-C(51)-C(56)-C(55)**	−1.4(3)
**C(52)-C(53)-C(54)-C(55)**	−1.2(3)
**C(53)-C(54)-C(55)-F(51)**	−177.85(19)
**C(53)-C(54)-C(55)-C(56)**	1.3(4)
**C(54)-C(55)-C(56)-C(51)**	0.0(4)
**C(56)-C(51)-C(52)-N(51)**	−178.2(2)
**C(56)-C(51)-C(52)-C(53)**	1.5(3)
**C(57)-N(51)-C(52)-C(51)**	−119.5(2)
**C(57)-N(51)-C(52)-C(53)**	60.9(3)
**C(57)-N(51)-C(63)-O(51)**	−4.2(3)
**C(57)-C(59)-C(62)-C(58)**	−1.4(3)
**C(57)-C(60)-C(61)-C(58)**	−1.1(3)
**C(57)-C(60)-C(64)-O(52)**	−178.50(19)
**C(59)-C(57)-C(60)-C(61)**	1.0(3)
**C(59)-C(57)-C(60)-C(64)**	−175.75(18)

**C(60)-C(57)-C(59)-C(62)**	0.3(3)
**C(61)-C(58)-C(62)-C(59)**	1.3(3)
**C(61)-C(60)-C(64)-O(52)**	4.8(3)
**C(62)-C(58)-C(61)-C(60)**	0.0(3)
**C(63)-N(51)-C(52)-C(51)**	48.8(3)
**C(63)-N(51)-C(52)-C(53)**	−130.8(2)
**C(63)-N(51)-C(57)-C(59)**	−117.3(2)
**C(63)-N(51)-C(57)-C(60)**	61.8(3)
**C(64)-C(60)-C(61)-C(58)**	175.70(19)

Table S8 Hydrogen bonds for balance **1**-H [Å and °].

**D-H...A**	**d(D-H)**	**d(H...A)**	**d(D...A)**	**<(DHA)**
**C(6)-H(6)...O(51)#1**	0.94(2)	2.38(2)	3.270(3)	159.4(19)
**C(9)-H(9)...F(1)#1**	0.98(2)	2.34(2)	3.302(3)	167.6(17)
**C(13)-H(13)...F(1)#2**	1.03(2)	2.50(3)	3.238(3)	127.9(17)
**C(14)-H(14)...O(51)#3**	1.01(3)	2.54(3)	3.391(3)	141.6(18)
**C(51)-H(51)...O(2)#4**	0.96(2)	2.48(2)	3.418(3)	165.6(17)
**C(53)-H(53)...O(1)#5**	0.97(2)	2.26(2)	3.192(3)	161.3(18)
**C(54)-H(54)...O(2)#5**	0.94(2)	2.59(2)	3.400(3)	144.6(16)
**C(59)-H(59)...F(51)#6**	0.97(2)	2.43(2)	3.159(3)	131.5(18)
**C(63)-H(63)...F(51)#7**	1.02(2)	2.60(2)	3.334(3)	128.9(14)

Symmetry transformations used to generate equivalent atoms:

#1 −x+1,-y+1, −z+1 #2 −x+1, −y+2, −z+1 #3 x,y+1,z

#4 x,y−1,z #5 x, −y+3/2,z+1/2 #6 −x,-y, −z+1

#7 −x,-y+1, −z+1

Full Crystallographic data for balance 1-Me

Table S9 Crystal data and structure refinement for balance **1**-Me.

**Identification code**	sc4001	
**Empirical formula**	$C_{15} H_{12} F N O_2$	
**Formula weight**	257.26	
**Temperature**	120.0 K	
**Wavelength**	1.54184 Å	
**Crystal system**	Monoclinic	
**Space group**	P 1 21/c 1	
**Unit cell dimensions**	a = 13.77538(18) Å	α = 90°.
	b = 8.10251(11) Å	β = 94.5274(12)°.
	c = 10.98340(15) Å	γ = 90°.
**Volume**	1222.09(3) Å^3	
**Z**	4	
**Density (calculated)**	1.398 Mg/m^3	
**Absorption coefficient**	0.863 mm^{-1}	
**F(000)**	536	
**Crystal size**	0.2834 × 0.1818 × 0.0829 mm^3	
**Theta range for data collection**	3.218 to 76.052°.	
**Index ranges**	−17<=h<=17, −8<=k<=10, −13<=l<=13	
**Reflections collected**	10324	
**Independent reflections**	2526 [R(int) = 0.0192]	
**Completeness to theta = 67.684°**	99.9 %	
**Absorption correction**	Gaussian	
**Max. and min. transmission**	0.983 and 0.961	
**Refinement method**	Full-matrix least-squares on F2	
**Data / restraints / parameters**	2526 / 0 / 221	
**Goodness-of-fit on F2**	1.060	
**Final R indices [I>2sigma(I)]**	R1 = 0.0306, wR2 = 0.0810	
**R indices (all data)**	R1 = 0.0321, wR2 = 0.0824	
**Extinction coefficient**	0.0036(5)	
**Largest diff. peak and hole**	0.252 and -0.181 e.Å-3	

Table S10 Atomic coordinates (× 10^4) and equivalent isotropic displacement parameters (Å^2 × 10^3) for balance **1**-Me. U(eq) is defined as one third of the trace of the orthogonalised U^{ij} tensor.

	x	y	z	U(eq)
**F(1)**	3644(1)	1671(1)	1356(1)	38(1)
**O(1)**	8698(1)	−1138(1)	3428(1)	23(1)
**O(2)**	7527(1)	−989(1)	5850(1)	24(1)
**N(1)**	7389(1)	602(1)	3376(1)	18(1)
**C(1)**	6252(1)	1148(1)	1595(1)	24(1)
**C(2)**	6420(1)	902(1)	2844(1)	18(1)
**C(3)**	5656(1)	914(1)	3594(1)	25(1)
**C(4)**	4710(1)	1185(2)	3100(1)	29(1)
**C(5)**	4562(1)	1420(1)	1857(1)	26(1)
**C(6)**	5305(1)	1406(1)	1093(1)	27(1)
**C(7)**	7859(1)	1820(1)	4164(1)	17(1)
**C(8)**	7844(1)	3456(1)	3770(1)	21(1)
**C(9)**	8347(1)	4663(1)	4461(1)	23(1)
**C(10)**	8882(1)	4233(1)	5538(1)	23(1)
**C(11)**	8369(1)	1385(1)	5269(1)	17(1)
**C(12)**	7873(1)	−768(1)	3037(1)	20(1)
**C(13)**	8315(1)	−320(1)	5804(1)	19(1)
**C(14)**	9247(1)	−1114(1)	6297(1)	24(1)
**C(16)**	8895(1)	2604(1)	5934(1)	20(1)

Table S11 Bond lengths [Å] and angles [°] for balance **1**-Me.

**F(1)-C(5)**	1.3550(12)
**O(1)-C(12)**	1.2202(12)
**O(2)-C(13)**	1.2176(12)
**N(1)-C(2)**	1.4349(12)
**N(1)-C(7)**	1.4334(12)
**N(1)-C(12)**	1.3616(13)
**C(1)-H(1)**	0.949(14)
**C(1)-C(2)**	1.3871(14)
**C(1)-C(6)**	1.3904(15)
**C(2)-C(3)**	1.3876(14)

**C(3)-H(3)**	0.978(15)
**C(3)-C(4)**	1.3885(15)
**C(4)-H(4)**	0.948(17)
**C(4)-C(5)**	1.3768(17)
**C(5)-C(6)**	1.3741(17)
**C(6)-H(6)**	0.953(16)
**C(7)-C(8)**	1.3935(14)
**C(7)-C(11)**	1.3997(13)
**C(8)-H(8)**	0.971(14)
**C(8)-C(9)**	1.3889(15)
**C(9)-H(9)**	0.977(14)
**C(9)-C(10)**	1.3881(15)
**C(10)-H(10)**	0.974(15)
**C(10)-C(16)**	1.3894(15)
**C(11)-C(13)**	1.5050(13)
**C(11)-C(16)**	1.3969(14)
**C(12)-H(12)**	1.001(13)
**C(13)-C(14)**	1.4992(14)
**C(14)-H(14A)**	0.943(16)
**C(14)-H(14B)**	0.954(18)
**C(14)-H(14C)**	0.965(19)
**C(16)-H(16)**	0.976(13)
**C(7)-N(1)-C(2)**	119.36(8)
**C(12)-N(1)-C(2)**	119.14(8)
**C(12)-N(1)-C(7)**	121.25(8)
**C(2)-C(1)-H(1)**	119.8(8)
**C(2)-C(1)-C(6)**	119.49(10)
**C(6)-C(1)-H(1)**	120.8(8)
**C(1)-C(2)-N(1)**	120.32(9)
**C(1)-C(2)-C(3)**	120.68(9)
**C(3)-C(2)-N(1)**	119.00(9)
**C(2)-C(3)-H(3)**	118.4(9)
**C(2)-C(3)-C(4)**	120.14(10)
**C(4)-C(3)-H(3)**	121.5(9)
**C(3)-C(4)-H(4)**	120.9(10)

**C(5)-C(4)-C(3)**	117.98(10)
**C(5)-C(4)-H(4)**	121.1(9)
**F(1)-C(5)-C(4)**	118.89(10)
**F(1)-C(5)-C(6)**	118.02(10)
**C(6)-C(5)-C(4)**	123.09(10)
**C(1)-C(6)-H(6)**	122.1(9)
**C(5)-C(6)-C(1)**	118.62(10)
**C(5)-C(6)-H(6)**	119.2(9)
**C(8)-C(7)-N(1)**	118.23(9)
**C(8)-C(7)-C(11)**	120.21(9)
**C(11)-C(7)-N(1)**	121.47(9)
**C(7)-C(8)-H(8)**	118.8(8)
**C(9)-C(8)-C(7)**	120.40(9)
**C(9)-C(8)-H(8)**	120.8(8)
**C(8)-C(9)-H(9)**	119.8(8)
**C(10)-C(9)-C(8)**	119.79(9)
**C(10)-C(9)-H(9)**	120.4(8)
**C(9)-C(10)-H(10)**	120.4(9)
**C(9)-C(10)-C(16)**	119.91(9)
**C(16)-C(10)-H(10)**	119.6(9)
**C(7)-C(11)-C(13)**	122.23(9)
**C(16)-C(11)-C(7)**	118.64(9)
**C(16)-C(11)-C(13)**	119.00(9)
**O(1)-C(12)-N(1)**	124.54(9)
**O(1)-C(12)-H(12)**	123.6(7)
**N(1)-C(12)-H(12)**	111.9(7)
**O(2)-C(13)-C(11)**	119.77(9)
**O(2)-C(13)-C(14)**	122.33(9)
**C(14)-C(13)-C(11)**	117.88(8)
**C(13)-C(14)-H(14A)**	110.8(9)
**C(13)-C(14)-H(14B)**	114.1(11)
**C(13)-C(14)-H(14C)**	106.3(11)
**H(14A)-C(14)-H(14B)**	109.7(14)
**H(14A)-C(14)-H(14C)**	110.1(14)
**H(14B)-C(14)-H(14C)**	105.6(15)

**C(10)-C(16)-C(11)**	120.98(9)
**C(10)-C(16)-H(16)**	119.2(8)
**C(11)-C(16)-H(16)**	119.8(8)

Table S12 Anisotropic displacement parameters (Å^2 × 10^3) for balance **1**-Me. The anisotropic displacement factor exponent takes the form: $-2\pi^2[h^2 a^{*2}U^{11} + ... + 2 h k a^* b^* U^{12}]$

	U^{11}	U^{22}	U^{33}	U^{23}	U^{13}	U^{12}
**F(1)**	20(1)	42(1)	49(1)	−3(1)	−10(1)	4(1)
**O(1)**	21(1)	22(1)	26(1)	−3(1)	1(1)	3(1)
**O(2)**	21(1)	26(1)	27(1)	4(1)	4(1)	−5(1)
**N(1)**	17(1)	18(1)	19(1)	−2(1)	0(1)	0(1)
**C(1)**	22(1)	30(1)	21(1)	0(1)	2(1)	−2(1)
**C(2)**	18(1)	17(1)	21(1)	−1(1)	0(1)	−1(1)
**C(3)**	23(1)	31(1)	22(1)	1(1)	3(1)	1(1)
**C(4)**	20(1)	36(1)	33(1)	−1(1)	6(1)	1(1)
**C(5)**	18(1)	22(1)	35(1)	−2(1)	−6(1)	1(1)
**C(6)**	28(1)	29(1)	23(1)	1(1)	−5(1)	−1(1)
**C(7)**	15(1)	18(1)	18(1)	−2(1)	4(1)	0(1)
**C(8)**	23(1)	20(1)	20(1)	2(1)	3(1)	2(1)
**C(9)**	25(1)	16(1)	29(1)	1(1)	6(1)	0(1)
**C(10)**	21(1)	21(1)	27(1)	−6(1)	5(1)	−4(1)
**C(11)**	15(1)	18(1)	19(1)	0(1)	4(1)	1(1)
**C(12)**	21(1)	19(1)	20(1)	−2(1)	3(1)	−1(1)
**C(13)**	20(1)	20(1)	16(1)	−1(1)	3(1)	−1(1)
**C(14)**	22(1)	22(1)	28(1)	7(1)	1(1)	−1(1)
**C(16)**	17(1)	23(1)	20(1)	−2(1)	2(1)	−1(1)

Table S13 Hydrogen coordinates (× 10^4) and isotropic displacement parameters (Å^2 × 10^3) for balance **1**-Me.

	**x**	**y**	**z**	**U(eq)**
**H(1)**	6782(10)	1141(17)	1094(13)	28(3)
**H(3)**	5801(11)	724(18)	4468(14)	34(4)
**H(4)**	4182(12)	1204(19)	3604(14)	43(4)

**H(6)**	5159(11)	1585(19)	240(15)	41(4)
**H(8)**	7485(10)	3731(17)	3000(13)	27(3)
**H(9)**	8325(10)	5808(18)	4181(12)	27(3)
**H(10)**	9237(10)	5071(19)	6028(13)	32(3)
**H(12)**	7470(9)	−1445(16)	2426(12)	22(3)
**H(14A)**	9128(11)	−1930(20)	6877(14)	40(4)
**H(14B)**	9723(13)	−360(20)	6640(16)	52(5)
**H(14C)**	9530(13)	−1610(20)	5607(17)	55(5)
**H(16)**	9268(9)	2318(17)	6698(12)	23(3)

Table S14 Torsion angles [°] for balance **1**-Me.

**F(1)-C(5)-C(6)-C(1)**	−179.94(10)
**N(1)-C(2)-C(3)-C(4)**	−179.34(10)
**N(1)-C(7)-C(8)-C(9)**	−175.46(9)
**N(1)-C(7)-C(11)-C(13)**	−10.78(14)
**N(1)-C(7)-C(11)-C(16)**	173.40(8)
**C(1)-C(2)-C(3)-C(4)**	−0.38(17)
**C(2)-N(1)-C(7)-C(8)**	−48.39(12)
**C(2)-N(1)-C(7)-C(11)**	135.20(9)
**C(2)-N(1)-C(12)-O(1)**	179.72(9)
**C(2)-C(1)-C(6)-C(5)**	0.44(17)
**C(2)-C(3)-C(4)-C(5)**	0.62(17)
**C(3)-C(4)-C(5)-F(1)**	179.41(10)
**C(3)-C(4)-C(5)-C(6)**	−0.35(18)
**C(4)-C(5)-C(6)-C(1)**	−0.18(17)
**C(6)-C(1)-C(2)-N(1)**	178.78(10)
**C(6)-C(1)-C(2)-C(3)**	−0.16(16)
**C(7)-N(1)-C(2)-C(1)**	112.06(11)
**C(7)-N(1)-C(2)-C(3)**	−68.98(12)
**C(7)-N(1)-C(12)-O(1)**	5.52(15)
**C(7)-C(8)-C(9)-C(10)**	1.20(15)
**C(7)-C(11)-C(13)-O(2)**	−46.58(13)
**C(7)-C(11)-C(13)-C(14)**	135.07(10)
**C(7)-C(11)-C(16)-C(10)**	2.74(14)

**C(8)-C(7)-C(11)-C(13)**	172.87(9)
**C(8)-C(7)-C(11)-C(16)**	−2.94(14)
**C(8)-C(9)-C(10)-C(16)**	−1.41(15)
**C(9)-C(10)-C(16)-C(11)**	−0.58(15)
**C(11)-C(7)-C(8)-C(9)**	1.00(15)
**C(12)-N(1)-C(2)-C(1)**	−62.25(13)
**C(12)-N(1)-C(2)-C(3)**	116.71(11)
**C(12)-N(1)-C(7)-C(8)**	125.80(10)
**C(12)-N(1)-C(7)-C(11)**	−50.62(13)
**C(13)-C(11)-C(16)-C(10)**	−173.21(9)
**C(16)-C(11)-C(13)-O(2)**	129.22(10)
**C(16)-C(11)-C(13)-C(14)**	−49.13(12)

Table S15 Hydrogen bonds for balance **1**-Me [Å and °].

**D-H...A**	**d(D-H)**	**d(H...A)**	**d(D...A)**	**<(DHA)**
**C(4)-H(4)...O(2)#1**	0.948(17)	2.481(17)	3.3781(13)	157.8(13)
**C(9)-H(9)...O(1)#2**	0.977(14)	2.672(14)	3.6308(13)	167.2(11)
**C(10)-H(10)...O(1)#3**	0.974(15)	2.924(14)	3.5563(13)	123.5(10)
**C(12)-H(12)...O(2)#4**	1.001(13)	2.710(13)	3.5665(12)	143.8(10)
**C(14)-H(14A)...O(1)#5**	0.943(16)	2.420(16)	3.3593(13)	174.5(13)
**C(14)-H(14B)...O(1)#6**	0.954(18)	2.494(18)	3.3604(13)	151.0(14)
**C(14)-H(14C)...O(1)**	0.965(19)	2.598(18)	3.1826(13)	119.2(13)

Symmetry transformations used to generate equivalent atoms:

#1 −x+1,-y,-z+1 #2 x,y+1,z #3 x, −y+1/2,z+1/2 #4 x, −y−1/2,z-1/2 #5 x, −y−1/2,z+1/2 #6 −x+2, −y, −z+1

S3. Experimental conformational free energies, ΔG_{exp}

S3.1 Experimental determination conformational free energies, ΔG_{exp}

NMR measurement of conformational free energies, ΔG_{exp}

Since rotation around the formamide bond is slow on the NMR timescale, discrete peaks corresponding to the "O" and "H" conformers (closed and open conformers, respectively) are observed. Thus, integration of the conformer peaks provides direct access to the conformational equilibrium constant, K, which can be used to determine the conformational free energy difference, ΔG_{exp} = −RT ln [closed]/[[open] = −RT ln K.[S1]

Conformational free energy (ΔG_{exp}) errors

The conformational free energy error $\delta(\Delta G_{exp})$ for the ΔG_{exp} values between −2.09 - +2.09 kJ mol^{-1} (*i.e.* conformer ratio ≥ 0.43 : 1) was estimated not to exceed ± 0.125 kJ mol^{-1}, which remains in accordance with other NMR-based literature studies of molecular balances.[S5] Integration errors associated with modern NMR spectrometers generally do not exceed 1–2%.[S6] Conformational free energy ΔG_{exp} values falling in the −2.09 to +2.09 kJ mol^{-1} range (*minor/major* peak integral ratio on NMR spectra ≥ 0.43) bear a ± 0.125 kJ mol^{-1} error, as described above. ΔG_{exp} values outside this range (< 2.09 kJ mol^{-1} and > 2.09 kJ mol^{-1}), with *minor/major* peak integral ratio on NMR spectra < 0.43 carry a conservative ± 3 % integration error (± 0.03 in the *minor/major* peak integral ratio), resulting in asymmetric ΔG_{exp} error margins.

Table S16 ΔG_{exp} values as determined by $^{19}F\{^1H\}$ NMR spectroscopy (376.5 MHz, 298 K) for all balance series **1** and control balances **S1**, **S2** and **S3** obtained in different solvents. Values for balances **S2** and **S3** (marked [a]) are obtained from the literature.[S1]

	ΔG_{exp}, kJ mol^{-1}	+error	−error	ΔG_{exp}, kJ mol^{-1}	+error	−error	ΔG_{exp}, kJ mol^{-1}	+error	−error	ΔG_{exp}, kJ mol^{-1}	+error	−error
		**1**-H			**1**-Me			**1**-OMe			**1**-NMe$_2$	
**Chloroform-*d***	1.77	0.125	0.125	−3.15	0.281	0.252	−1.44	0.125	0.125	−1.77	0.125	0.125
**Acetone**	2.53	0.198	0.216	−2.67	0.229	0.209	−1.62	0.125	0.125	−0.81	0.125	0.125
**Acetonitrile-*d*$_3$**	2.40	0.188	0.204	−2.33	0.198	0.184	−1.77	0.125	0.125	0.49	0.125	0.125
**Benzene-*d*$_6$**	2.09	0.125	0.125	−4.11	0.426	0.363	−1.67	0.125	0.125	−3.15	0.281	0.252
**Ethyl acetate**	2.15	0.171	0.184	−3.24	0.292	0.261	−1.44	0.125	0.125	−1.82	0.125	0.125
**Hexane**	0.92	0.125	0.125	−4.25	0.452	0.382	−0.99	0.125	0.125	−4.87	0.597	0.481
**THF**	2.09	0.167	0.179	−3.15	0.281	0.252	−1.31	0.125	0.125	−1.87	0.125	0.125
**DCM**	1.82	0.125	0.125	−2.60	0.222	0.204	−1.44	0.125	0.125	−0.96	0.125	0.125
**Ethanol**			*n.a.*	−3.07	0.271	0.244	−1.92	0.125	0.125	1.03	0.125	0.125
**Methanol**			*n.a.*	−2.98	0.261	0.236	−2.03	0.175	0.163	0.71	0.125	0.125
**DMSO-*d*$_6$**	2.82	0.222	0.244	−2.67	0.229	0.209	−2.27	0.193	0.179	0.65	0.125	0.125
**Diethyl ether**	1.62	0.125	0.125	−3.54	0.331	0.292	−1.07	0.125	0.125	−2.98	0.261	0.236
**CCl_4**	1.31	0.125	0.125	−4.25	0.452	0.382	−1.14	0.125	0.125	−4.39	0.481	0.403
**CS_2**	1.14	0.125	0.125	−4.87	0.597	0.481	−1.22	0.125	0.125	−3.64	0.346	0.304
		**S1**-F			**S2**-OMe[a]			**S3**-Me[a]				
**Chloroform-*d***	0.40	0.125	0.125	−0.15	0.125	0.125	0.75	0.125	0.125			
**Acetone**	0.85	0.125	0.125	−0.55	0.125	0.125	0.81	0.125	0.125			
**Acetonitrile-*d*$_3$**	1.11	0.125	0.125	−1.18	0.125	0.125	0.13	0.125	0.125			
**Benzene-*d*$_6$**	0.88	0.125	0.125	0.15	0.125	0.125	0.78	0.125	0.125			
**Ethyl acetate**	0.62	0.125	0.125	0.05	0.125	0.125	1.07	0.125	0.125			
**Hexane**	0.23	0.125	0.125	1.35	0.125	0.125	1.87	0.125	0.125			
**THF**	0.46	0.125	0.125	0.13	0.125	0.125	1.44	0.125	0.125			
**DCM**	0.65	0.125	0.125	0.29	0.125	0.125	0.55	0.125	0.125			
**Ethanol**	0.96	0.125	0.125	−0.78	0.125	0.125	0.49	0.125	0.125			
**Methanol**	1.14	0.125	0.125	−1.18	0.125	0.125	0.02	0.125	0.125			
**DMSO-*d*$_6$**	1.53	0.125	0.125	−1.48	0.125	0.125	−0.08	0.125	0.125			
**Diethyl ether**	0.10	0.125	0.125	0.88	0.125	0.125	1.82	0.125	0.125			
**CCl_4**	0.02	0.125	0.125			*n.a.*			*n.a.*			
**CS_2**	0.10	0.125	0.125			*n.a.*			*n.a.*			

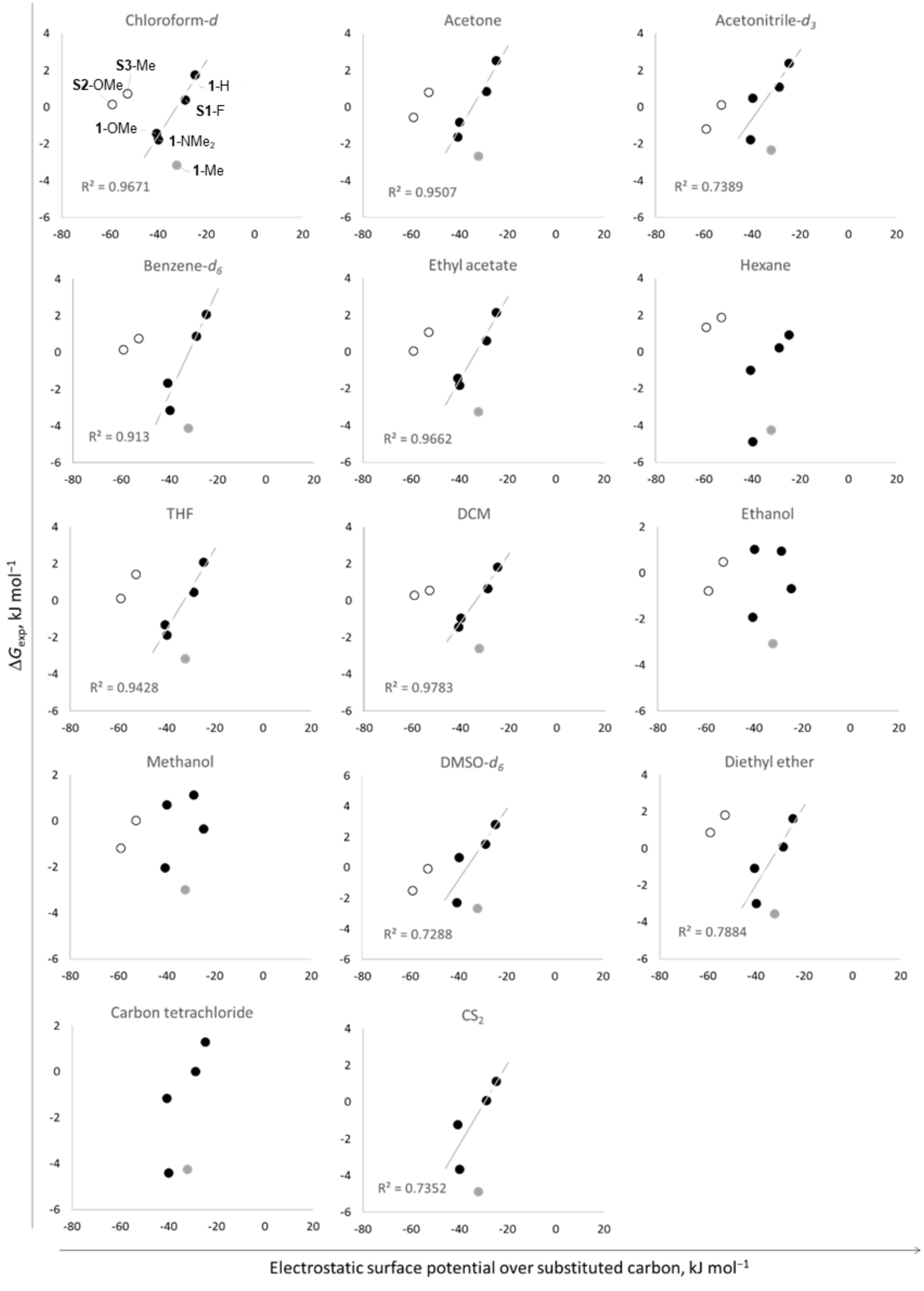

Figure S30 Plots of ΔG_{exp} in different solvents *vs.* ESP over the substituted carbon (**Table S24**, DFT/B3LYP/6-31G*). Error bars have been omitted for clarity.

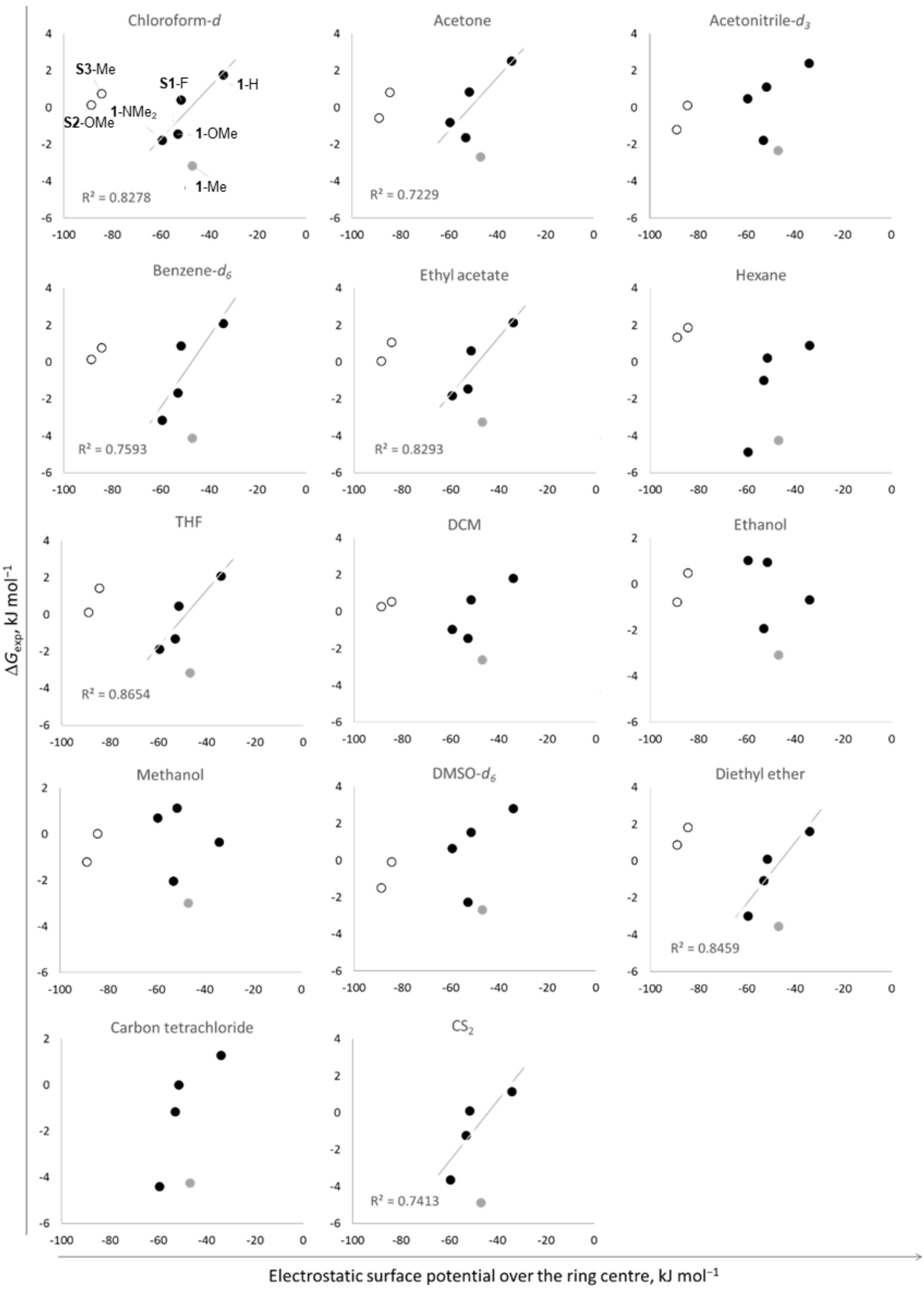

Figure S31 Plots of ΔG_{exp} in different solvents *vs.* ESP over the centre of the substituted ring (**Table S24**, DFT/B3LYP/6-31G*). Error bars have been omitted for clarity.

S3.2 Correlations of solvent parameters with ΔG_{exp}

Table S17 Solvent parameters used for correlations with ΔG_{exp} in. [a] Values of α_s and β_s retrieved from references.[S7] [b] $E_T(30)$ values retrieved from reference.[S8] [c] Dielectric constant ε values retrieved from reference.[S9] [d] Cohesive energy density values retrieved from reference.[S10] [e] Hildebrand parameter values (square root of c.e.d.) retrieved from references.[S7c, S11] [f] Values calculated at the DFT/B3LYP/6-31G* level of theory. [g] Hansen solubility parameters values retrieved from reference.[S12]

Solvent	α_s[a]	β_s[a]	$\alpha_s\beta_s$[a]	$E_T(30)$[b], kcal mol^{-1}	Dielectric constant ε[c], kcal mol^{-1}	Cohesive energy density (c.e.d.)[d], cal cm^{-3}	Hildebrand parameter (δ_H)[e], (cal cm^3)$^{1/2}$	Electrostatic potential min[f], kJ mol^{-1}	molecular surface area[f], km^2 dm^{-3}	Hansen parameters δ_P+δ_{HB}[g]
**Chloroform**	2.2	0.9	2.0	39.1	4.8	85.4	9.2	−37.09	7.51	8.80
**Acetone**	1.5	5.8	8.7	42.2	21.0	94.3	9.7	−177.08	7.85	17.40
**Acetonitrile**	1.7	5.1	8.7	46.0	36.6	139.2	11.8	−191.14	8.47	24.10
**Benzene**	1.1	2.1	2.3	34.3	2.3	83.7	9.2	−88.30	7.77	2.00
**Ethyl acetate**	1.5	5.3	8.0	38.1	-	81.7	9.0	-	7.82	-
**Hexane**	1.2	0.6	0.7	31.0	1.9	52.4	7.2	−21.91	7.01	0.00
**THF**	0.9	5.9	5.3	37.4	7.5	86.9	9.3	−185.05	7.85	13.70
**DCM**	1.9	1.1	2.1	40.7	8.9	98.5	9.9	−61.94	7.91	11.45
**Ethanol**	2.7	5.3	14.3	52.0	25.3	161.3	12.7	−178.91	8.46	28.20
**Methanol**	2.7	5.3	14.3	55.5	33.0	208.8	14.5	−189.82	9.10	34.60
**DMSO**	2.2	8.7	19.1	45.1	47.2	168.6	13.0	−231.65	8.81	26.60
**Diethyl ether**	0.9	5.3	4.8	34.5	-	59.9	7.7		7.24	
**CCl_4**	1.4	0.6	0.8	32.4	2.2	73.6	8.6	−15.08	7.24	0.60
**CS_2**	1.0	0.1	0.1	32.6	2.6	100.0	10.0	−2.96	7.81	0.60

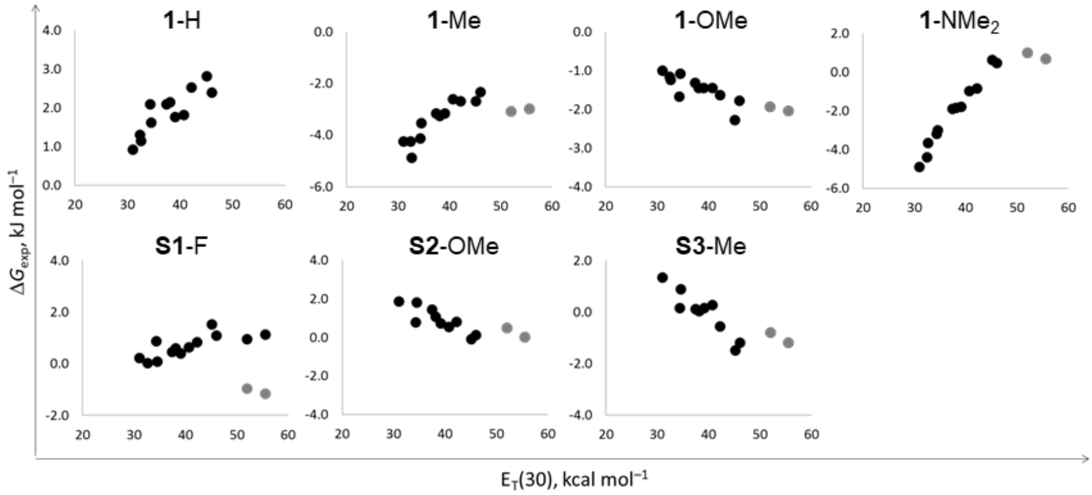

Figure S32 Correlation of ΔG_{exp} *vs.* E▯(30). Grey data points correspond to measurements in methanol and ethanol (outliers).

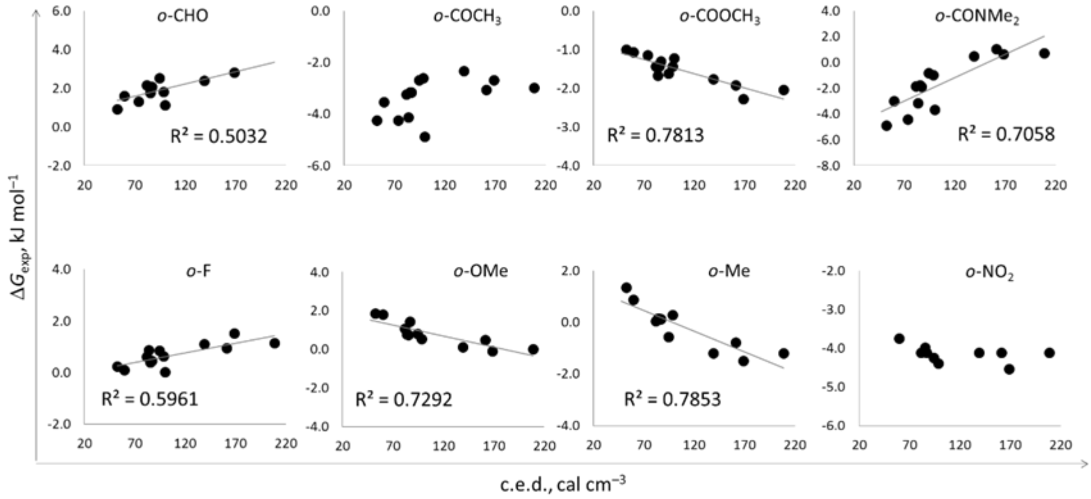

Figure S33 Correlation of ΔG_{exp} *vs*. cohesive energy density (c.e.d.).

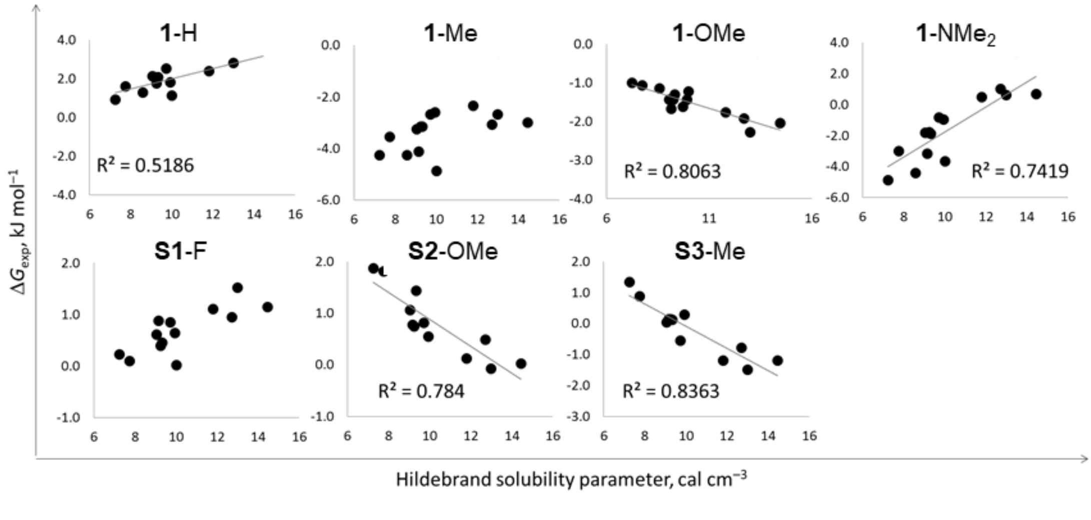

Figure S34 Correlation of ΔG_{exp} *vs*. Hildebrand solubility parameter.

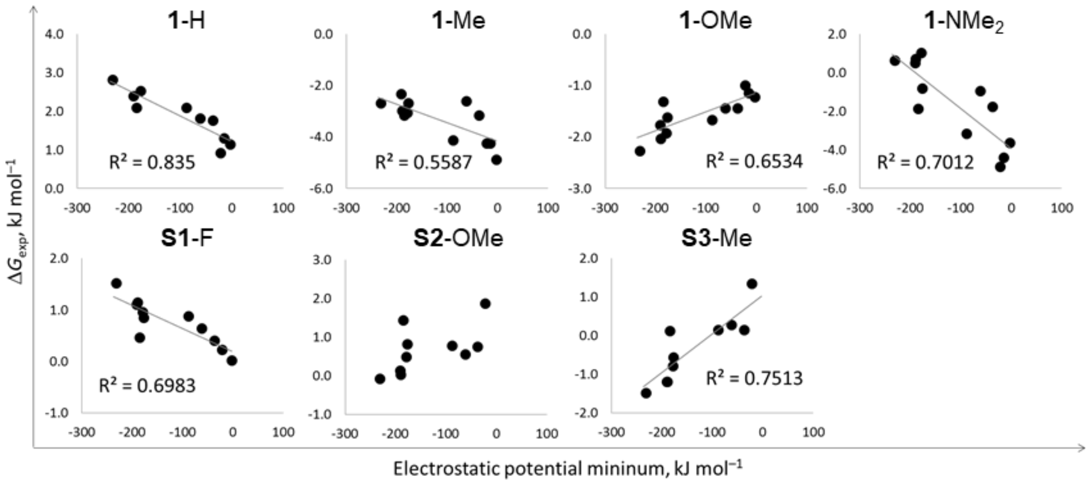

Figure S35 Correlation of ΔG_{exp} *vs*. electrostatic potential minimum of the solvent (DFT/B3LYP/6-31G*).

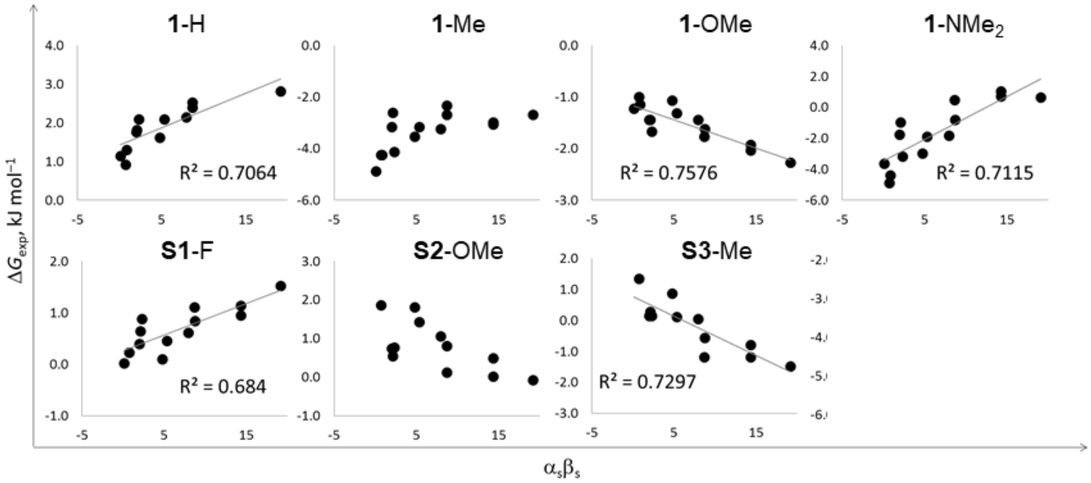

Figure S36 Correlation of ΔG_{exp} *vs*. $\alpha_\sigma\beta_s$ parameter, where α_s and β_s are solvent H-bond donor and acceptor parameters, respectively.

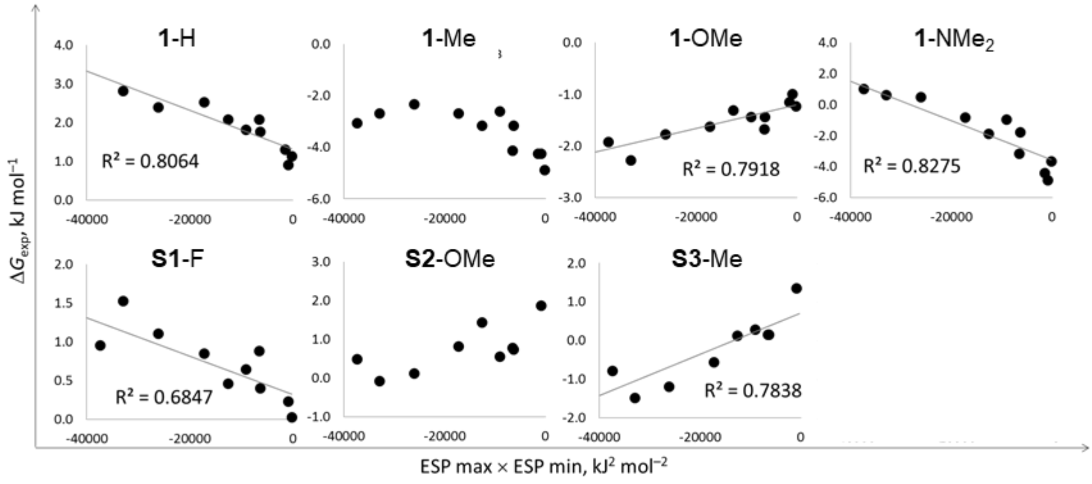

Figure S37 Correlation of ΔG_{exp} *vs*. cross-product of ESP maximum x ESP minimum of the solvent (estimate of solvophobicity, DFT/B3LYP/6-31G*)

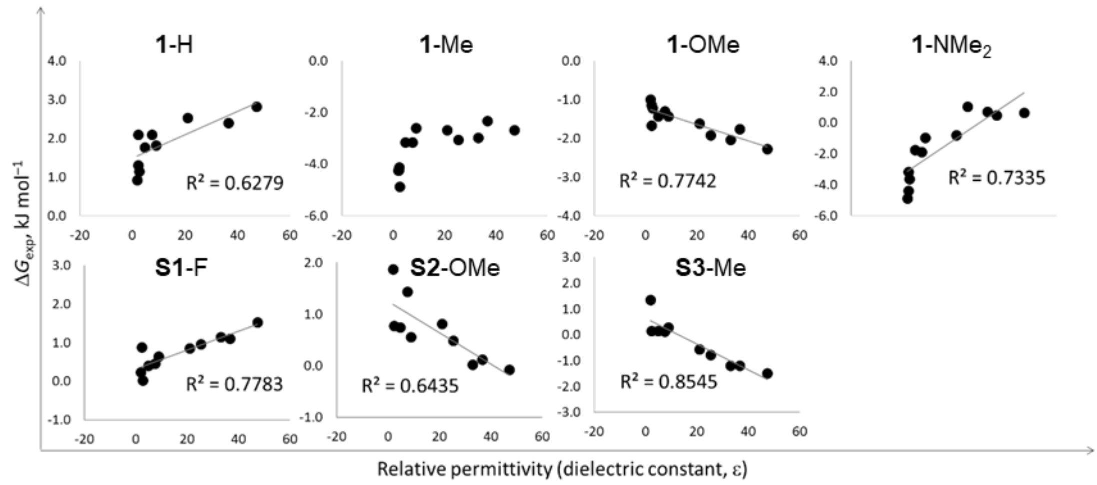

Figure S38 Correlation of ΔG_{exp} *vs*. relative permittivity (dielectric constant, ε).

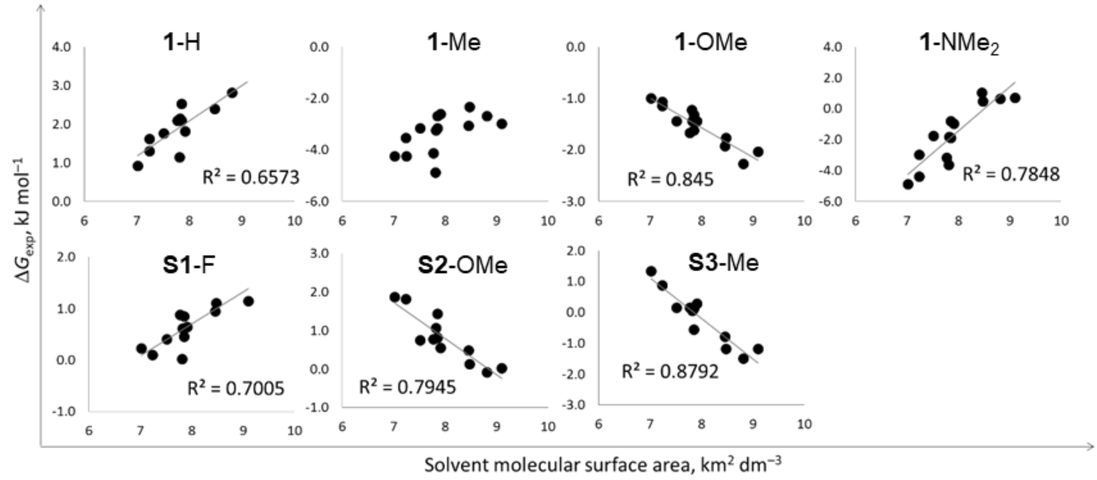

Figure S39 Correlation of ΔG_{exp} *vs*. molecular surface area of the solvent (DFT/B3LYP/6-31G*).

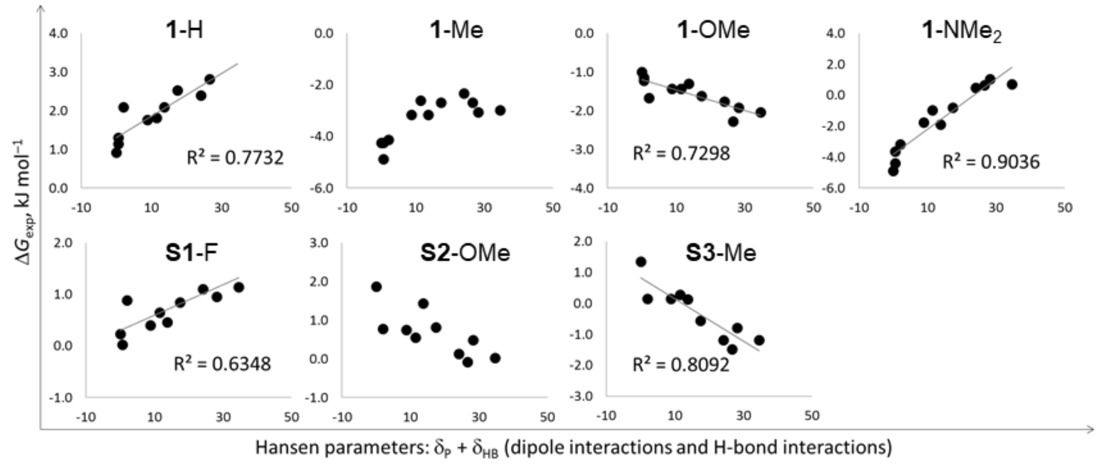

Figure S40 Correlation of ΔG_{exp} *vs*. Hansen solubility parameters $\delta_P + \delta_{HB}$ (dipole interactions and H-bond interactions).

S3.3 Application of the Hunter solvation model

The data can be further analysis based on the Hunter solvation model[S1, S7a, S7b] (**Figure S41** and **Equation S1**), where two parameters describe solvent polarity (α_s and β_s, describing H-bond donor and acceptor ability of the solvent respectively).

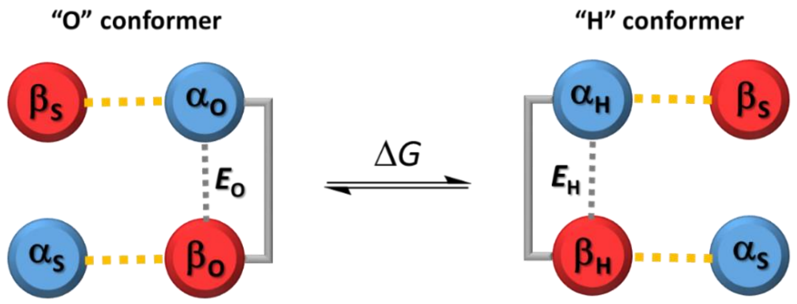

Figure S41 Hunter's solvation model adapted for the formamide balances.[S1, S7a, S7b] E_H and E_O correspond to intramolecular interactions in the "H" and "O" conformer (or open/closed conformers respectively). α_s and β_s correspond to H-bond donor and acceptor constants of the solvent respectively. α_O and β_O correspond to to H-bond donor and acceptor constants of the O conformer respectively, and α_H and β_H correspond to H-bond donor and acceptor constants of the H conformer respectively.

Equation S1 describes the Hunter solvation model as adapted for formamide balances.[S1, S7a, S7b]

$$\Delta G_{calc} = E_H - E_O + \alpha_s(\beta_O - \beta_H) + \beta_s(\alpha_O - \alpha_H) = \Delta E_{exp} + \alpha_s\Delta\beta + \beta_s\Delta\alpha \quad \textbf{Equation S1}$$

ΔE_{exp} is the intramolecular interaction energy, and $\Delta\alpha$ and $\Delta\beta$ are the differences in the H-bond donor and acceptor constants between the "O" and "H" conformers (also referred to as closed and open conformers respectively) (understood globally for each conformer, i.e. Boltzmann-averaged).

Calculated conformational free energy (ΔG_{calc}) errors

Calculated conformational free energy (ΔG_{calc}) errors were calculated using the following equation:

$$\delta\Delta G_{calc} = \sqrt{(\delta\Delta E)^2 + (\delta\Delta\alpha)^2 + (\delta\Delta\beta)^2} \quad \textbf{Equation S2}$$

where $\delta\Delta E$, $\delta\Delta\alpha$ and $\delta\Delta\beta$ are standard multilinear regression fitting errors, as output by *Origin v9.0*.

Table S18 ΔE_{exp}, $\Delta\beta$ and $\Delta\alpha$ values obtained by fitting the conformational free energies ΔG_{exp} measured in different solvents to **Equation S1**. Errors correspond to standard multilinear regression fitting errors as output by *Origin v.9.0* software.

Balance	ΔE_{exp}, kJ mol^{-1}	$\Delta\beta$	$\Delta\alpha$
**1**-H	3.09 ± 0.73	−1.10 ± 0.36	0.05 ± 0.09
**1**-Me	−4.45 ± 0.44	0.40 ± 0.22	0.15 ± 0.05
**1**-OMe	−0.76 ± 0.24	−0.26 ± 0.12	−0.10 ± 0.03
**1**-NMe_2	−5.31 ± 0.81	1.19 ± 0.40	0.37 ± 0.10
**S1**-F	−0.09 ± 0.30	0.21 ± 0.15	0.11 ± 0.04
**S2**-OMe[a]	2.10 ± 0.46	−0.54 ± 0.23	−0.11 ± 0.06
**S3**-Me[a]	1.95 ± 0.45	−0.64 ± 0.22	−0.26 ± 0.06

[a] values of ΔE_{int}, $\Delta\beta$ and $\Delta\alpha$, as well as full analysis of solvent effects was obtained from the literature[S1]

Table S19 ΔG_{calc} values determined for all balances by fitting to the Hunter solvation model (**Equation S1**).

	1-H	1-Me	1-OMe	1-NMe$_2$	S1-F	S2-OMe	S3-Me
**Chloroform-*d***	0.82 ± 0.82	−3.47 ± 0.49	−1.39 ± 0.27	−2.47 ± 0.91	0.46 ± 0.34	0.88 ± 0.51	0.37 ± 0.51
**Acetone**	1.71 ± 0.82	−2.99 ± 0.49	−1.71 ± 0.27	−1.35 ± 0.91	0.88 ± 0.34	0.67 ± 0.51	−0.52 ± 0.51
**Acetonitrile-*d*$_3$**	1.46 ± 0.82	−3.00 ± 0.49	−1.70 ± 0.27	−1.33 ± 0.91	0.85 ± 0.34	0.63 ± 0.51	−0.49 ± 0.51
**Benzene-*d*$_6$**	2.09 ±0.82	−3.74 ± 0.49	−1.22 ± 0.27	−3.33 ± 0.91	0.36 ± 0.34	1.34 ± 0.51	0.76 ± 0.51
**Ethyl acetate**	1.69 ± 0.82	−3.06 ± 0.49	−1.66 ± 0.27	−1.54 ± 0.91	0.82 ± 0.34	0.73 ± 0.51	−0.39 ± 0.51
**Hexane**	2.01 ± 0.82	−3.97 ± 0.49	−1.07 ± 0.27	−3.93 ± 0.91	0.18 ± 0.34	1.51 ± 0.51	1.18 ± 0.51
**THF**	2.38 ± 0.82	−3.21 ± 0.49	−1.56 ± 0.27	−2.02 ± 0.91	0.76 ± 0.34	0.98 ± 0.51	−0.16 ± 0.51
**DCM**	1.05 ± 0.82	−3.52 ± 0.49	−1.36 ± 0.27	−2.63 ± 0.91	0.44 ± 0.34	0.96 ± 0.51	0.45 ± 0.51
**Ethanol**	0.36 ± 0.82	−2.58 ± 0.49	−1.97 ± 0.27	−0.11 ± 0.91	1.08 ± 0.34	0.08 ± 0.51	−1.16 ± 0.51
**Methanol**	0.36 ± 0.82	−2.58 ± 0.49	−1.97 ± 0.27	−0.11 ± 0.91	1.08 ± 0.34	0.08 ± 0.51	−1.16 ± 0.51
**DMSO-*d*$_6$**	2.63 ± 0.82	−2.81 ± 0.49	−1.82 ± 0.27	−1.02 ± 0.91	1.08 ± 0.34	0.72 ± 0.51	−0.88 ± 0.51
**Diethyl ether**	2.35 ± 0.82	−3.30 ± 0.49	−1.50 ± 0.27	−2.25 ± 0.91	0.70 ± 0.34	1.05 ± 0.51	−0.01 ± 0.51
**CCl$_4$**	1.58 ± 0.82	−3.80 ± 0.49	−1.18 ± 0.27	−3.42 ± 0.91	0.28 ± 0.34	1.29 ± 0.51	0.90 ± 0.51

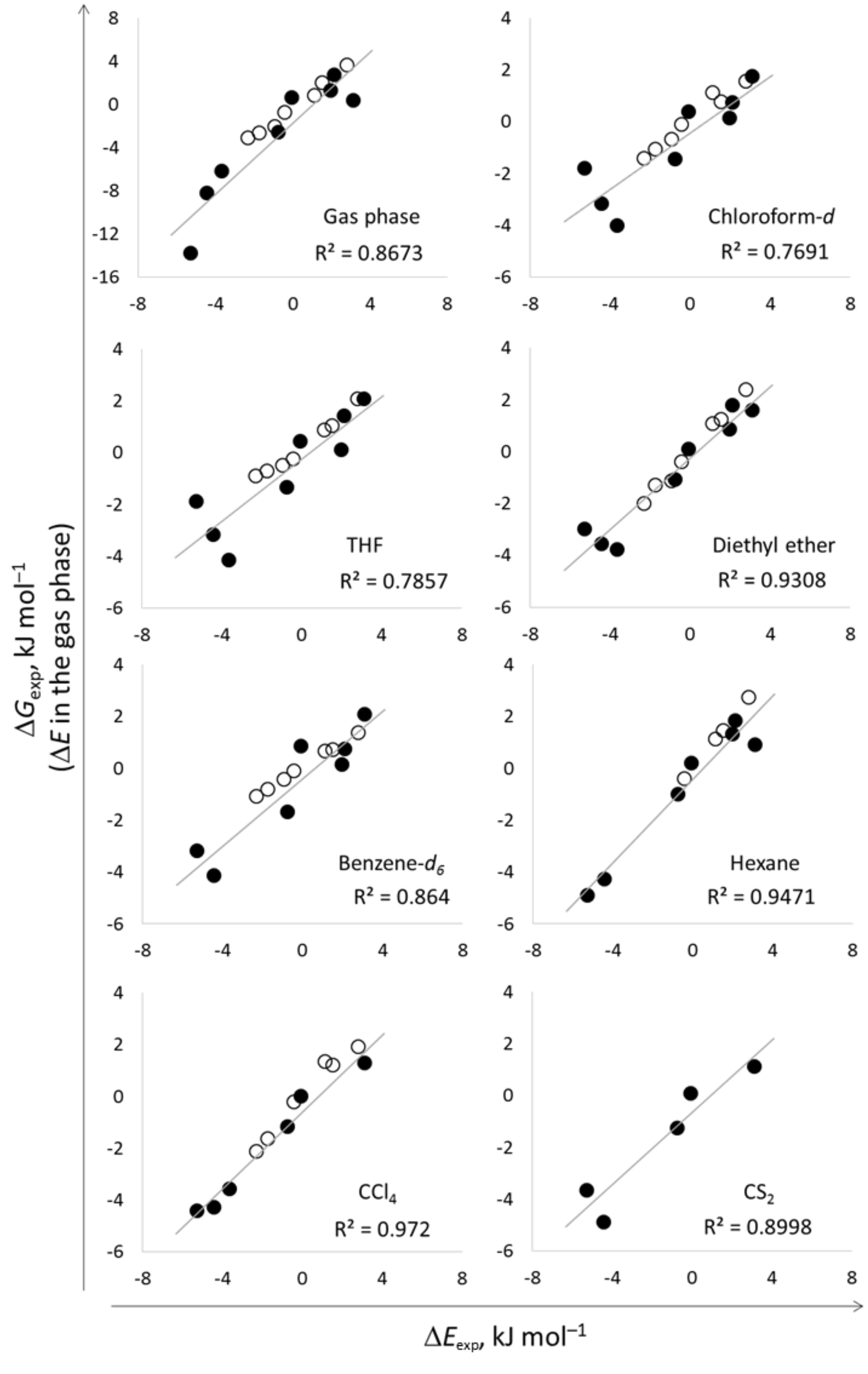

Figure S42 Correlation of ΔG_{exp} (ΔE in gas phase calculations (**Table S21**, B3LYP/6-31G*) and conformational free energies obtained in weakly solvating solvents for all balances) *vs*. ΔE_{exp} obtained using the solvation model. Filled circles correspond to *ortho*-substituted balances (carbonyl series and controls), hollow circles correspond to representative examples of the *para*-substituted series (for comparison where available: *p*-NEt_2, *p*-OMe, *p*-H, *p*-Br, Pyr, *p*-CN, *p*-NO_2; values of ΔG_{exp} (ΔE) and ΔE_{exp} from literature data.[S1] ΔG_{exp} errors have been omitted for clarity.

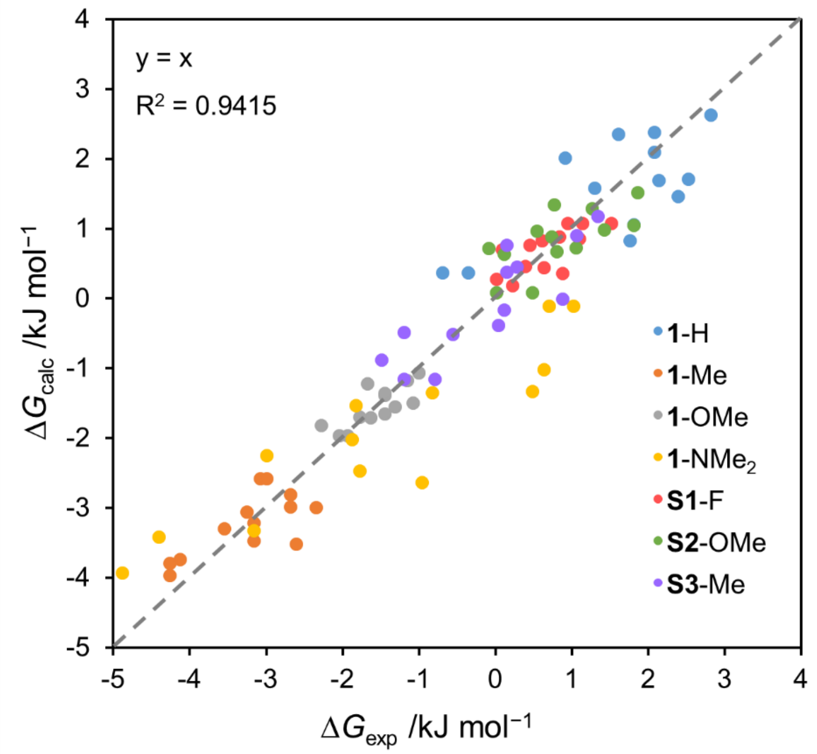

Figure S43 Plot of ΔG_{exp} *vs*. ΔG_{calc} for all studied balances in up to thirteen solvents each. Error bars have been omitted for clarity.

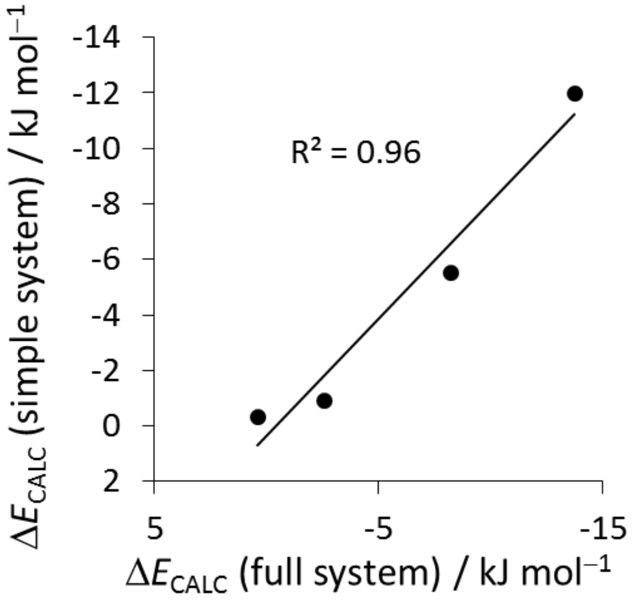

Figure S44 Plot of conformational energy differences between full molecular balances and molecular balance series **1**-X. Calculated using B3LYP/6-31G*.

S4. Computational methods and data

S4.1 Geometry minimisation and calculated conformational free energies

Full molecular balance structures shown in **Figures S1** and **S2** were minimised in both the open and closed conformations using either the B3LYP or ωB97X-D methods and basis sets 6-31G* in Spartan ’14. Frequency calculations were performed on all minimised structures, which confirmed no imaginary frequencies. The resulting gas-phase energies and corresponding energy differences, ΔE_{calc} in each conformer are reported.

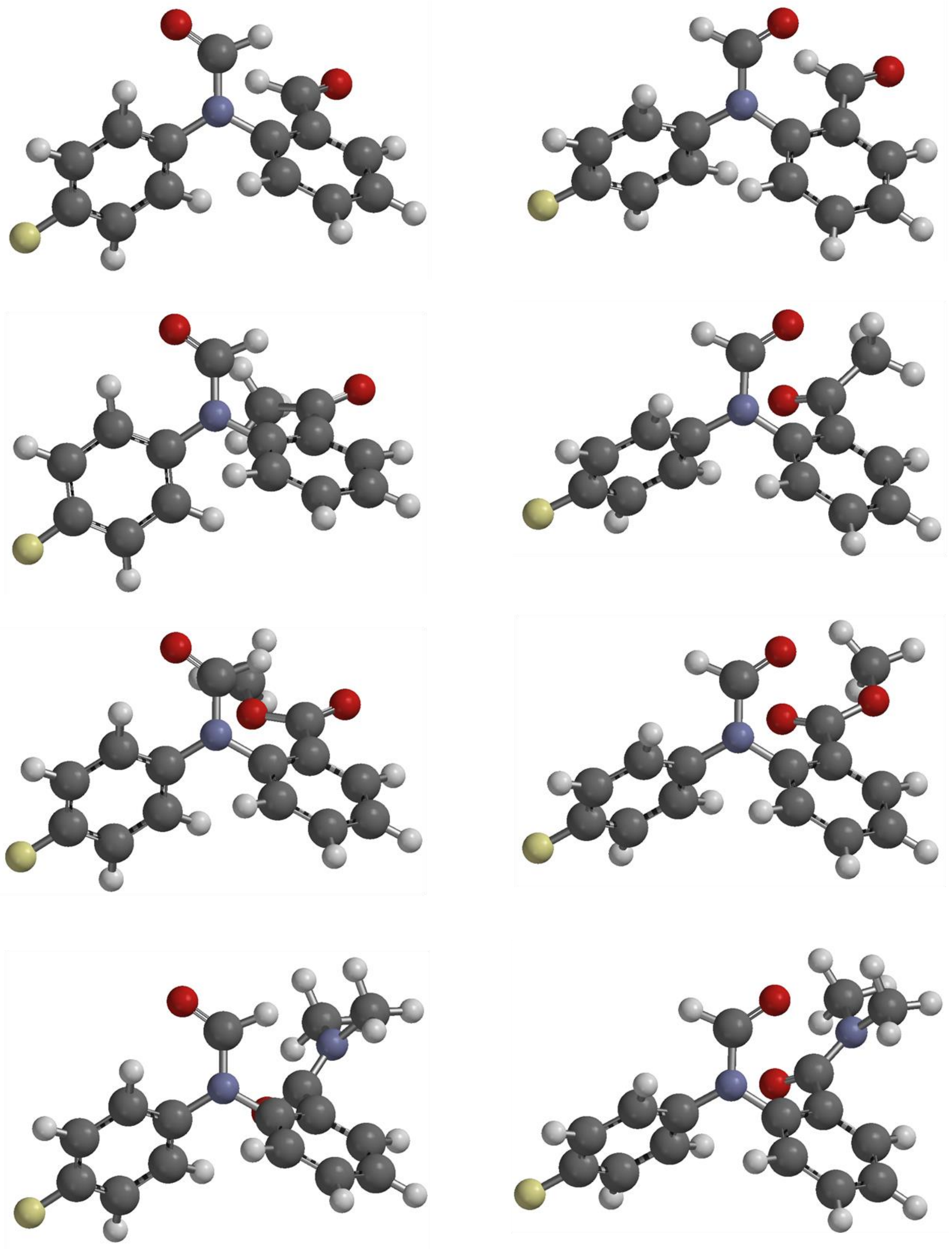

Figure S45 Minimised geometries of the open and closed conformers of molecular balances in series **1**-X. Minimised using Spartan ’14 DFT/B3LYP/6-31G*.

Only minimal differences were observed between minimised structures using either B3LYP or ωB97X-D methods (**Figure S46**).

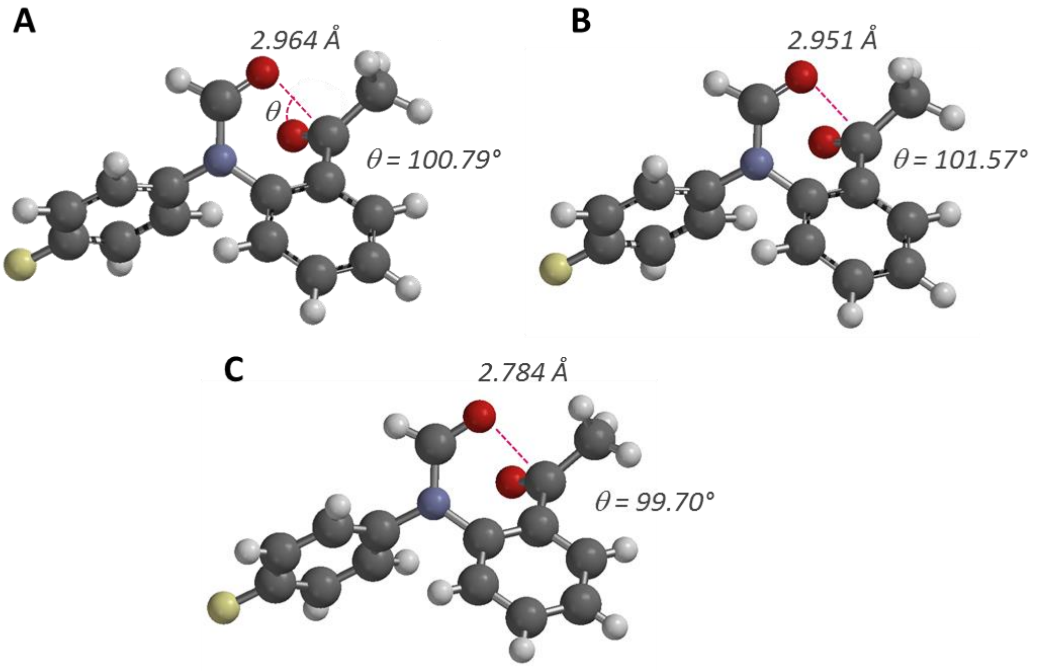

Figure S46 Structures of molecular balance **1**-Me minimised using **A** DFT/B3LYP/6-31G*, **B** DFT/ωB97X-D/6-31G* and **C** crystal structure. Angle θ is defined as the O···C=O angle.

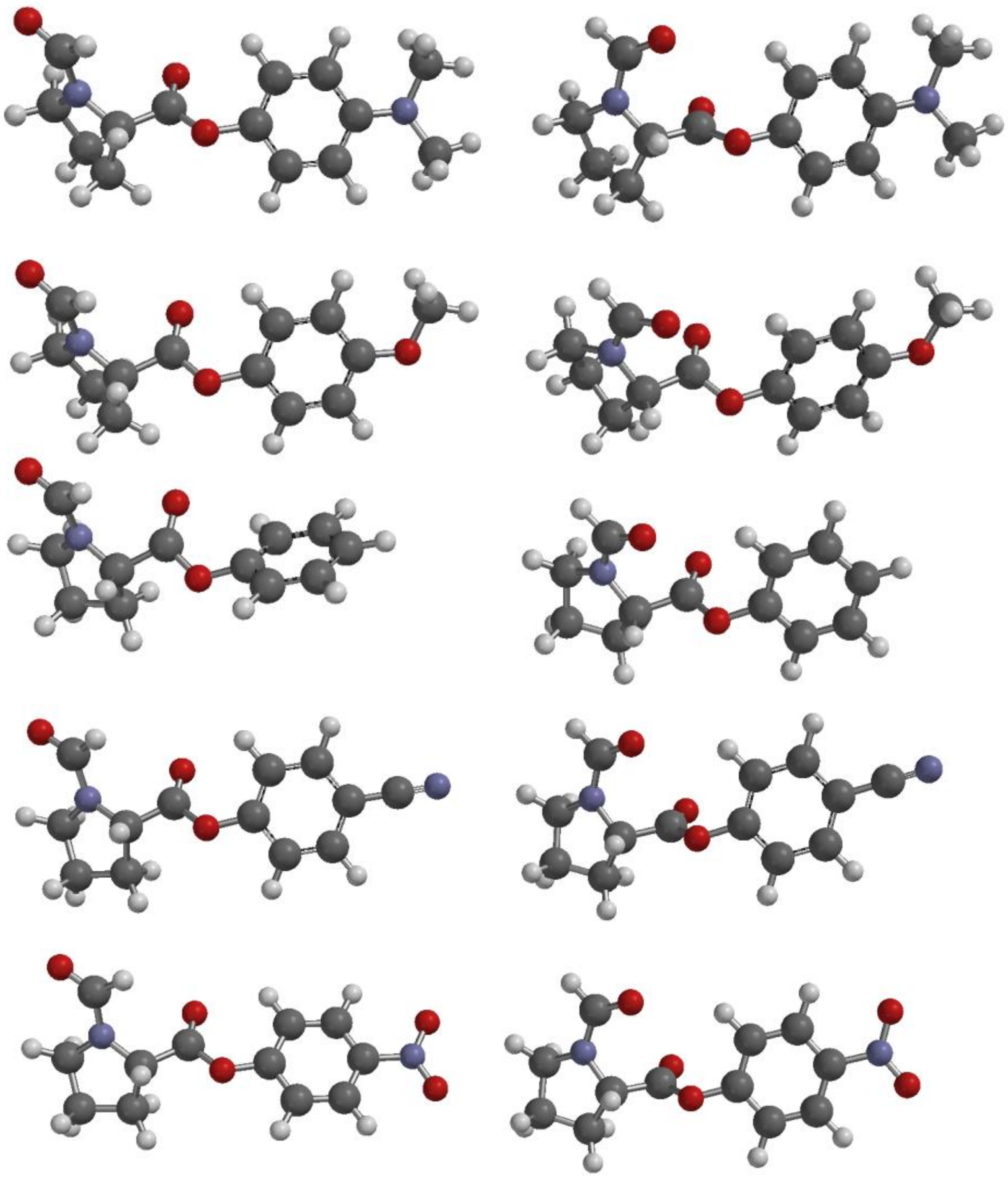

Figure S47 Minimised geometries of the open and closed conformers of molecular balances in series **3**-Y. Minimised using Spartan ’14 DFT/B3LYP/6-31G*.

Table S20 Distance and interaction angles between carbonyl donor and acceptor groups in the closed conformer for molecular balances in series **1**-X, **2** and **3**-Y (**Figures S1** and **S2**). Calculated using Spartan ’14 DFT/B3LYP/6-31G* except for balance **2**, which is the X-ray geometry.

Balance	Carbonyl···carbonyl distance / Å	Interaction angle / °
**1**-H	3.062	130.2
**1**-Me	2.964	100.8
**1**-OMe	3.022	90.0
**1**-NMe_2	3.070	110.71
**2** (X-ray)	3.333	109.3
**3**-NO_2	2.797	99.8
**3**-CN	2.803	99.6
**3**-H	2.844	100.4
**3**-OMe	2.900	98.9
**3**-NMe_2	2.931	98.3

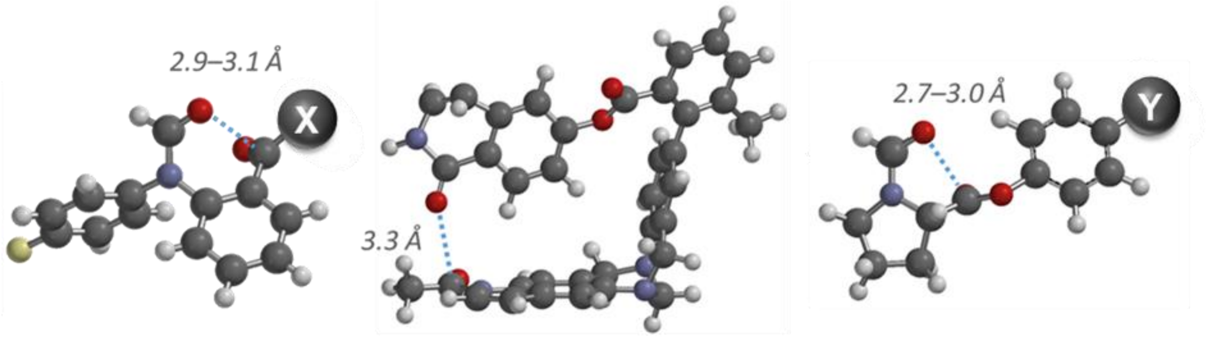

1-X **2** **3**-Y

Figure S48 Summary of the O···C distances observed in the minimised structures of balances **1**-X, **3**-Y and the X-ray structure of **2**.

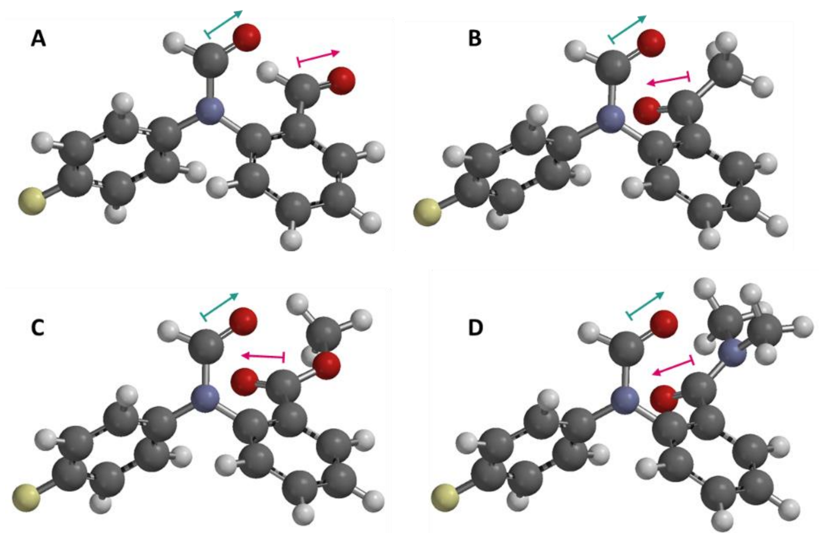

Figure S49 Minimised structures of molecular balances **A** **1**-H, **B** **1**-Me, **C** **1**-OMe and **D** **1**-NMe_2 showing dipoles for the donor (teal) and acceptor (pink) carbonyls. The orientation of the *ortho*-carbonyl acceptor group is different in **1**-H than the other molecular balances resulting in repulsive local dipoles. Structures minimised using Spartan '14 DFT/B3LYP/6-31G*.

Table S21 Calculated conformational energy differences of full balance series **1**-X calculated and minimised using Spartan '14 DFT/B3LYP/6 31G*.

**Balance**	**Closed conformer *E* / kJ mol^{-1}**	**Open conformer *E* / kJ mol^{-1}**	**ΔE_{calc} / kJ mol^{-1}**
**1**-H	−2217335.71	−2217335.29	0.42
**1**-Me	−2320569.35	−2320577.52	−8.17
**1**-OMe	−2518095.02	−2518097.55	−2.53
**1**-NMe_2	−2569138.45	−2569152.16	−13.71

Table S22 Calculated conformational energy differences of full balance series **1**-X calculated and minimised using Spartan '14 DFT/ωB97X-D/6 31G*.

**Balance**	**Closed conformer *E* / kJ mol^{-1}**	**Open conformer *E* / kJ mol^{-1}**	**ΔE_{calc} / kJ mol^{-1}**
**1**-H	−2216627.37	−2216627.31	0.06
**1**-Me	−2319841.55	−2319847.04	−5.49
**1**-OMe	−2517304.40	−2517306.31	−1.91
**1**-NMe_2	−2568342.41	−2568356.72	−14.31

Table S23 Calculated conformational energy differences of full balance series **3**-**Y** calculated and minimised using Spartan '14 DFT/B3LYP/6 31G*.

Balance	Open conformer *E* / kJ mol^{-1}	Closed conformer *E* / kJ mol^{-1}	ΔE_{calc} / kJ mol^{-1}
**3**-NO_2	−2494332	−2494343	−10.9
**3**-CN	−2199599	−2199608	−9.7
**3**-H	−1957417	−1957423	−6.0
**3**-OMe	−2258097	−2258100	−3.6
**3**-NMe_2	−2309148	−2309151	−2.8

S4.2 Electrostatic Surface Potentials

Electrostatic Surface Potentials were obtained using *Spartan '14* software on minimised balance structures (DFT/B3LYP/6-31G*). The ESP errors were determined as standard deviation from four ESP measurements (two measurements on the position of interest on both faces of the aromatic ring).

Table S24 ESP calculated for X-substituted rings. Values measured over the substituted carbon or over the centre of the ring. [a] very large error due to asymmetric geometry and large ESP differences between both sides of the ring

Balance	ESP over subst. C (*ortho* to amide) / kJ mol^{-1}	ESP over ring centre / kJ mol^{-1}
**1**-H	−24.7 ± 0.2	−34.2 ± 0.3
**1**-Me	−32.3 ± 1.1	−47.0 ± 0.2
**1**-OMe	−40.7 ± 0.8	−53.0 ± 0.3
**1**-NMe_2	−39.9 ± 48.8[a]	−59.5 ± 14.6[a]
**S1**-F	−28.8 ± 1.1	−51.6 ± 0.4
**S2**-OMe	−52.9 ± 0.5	−84.5 ± 0.2
**S3**-Me	−59.1 ± 4.4	−88.9 ± 0.5

S4.3 fiSAPT analysis

The PSI4 software[S13] was used to perform functional group intramolecular symmetry adapted perturbation theory (fiSAPT) calculations using the SAPT0 methodology.[S14] Geometries from minimisations performed in Spartan ’14 were used as an input for fiSAPT calculations. The 6-311G*, jun-cc-pVDZ or aug-cc-pVQZ basis sets were used. The output gave the energetic contributions of electrostatics, induction, exchange-repulsion and dispersion to give a total SAPT interaction energy prediction.

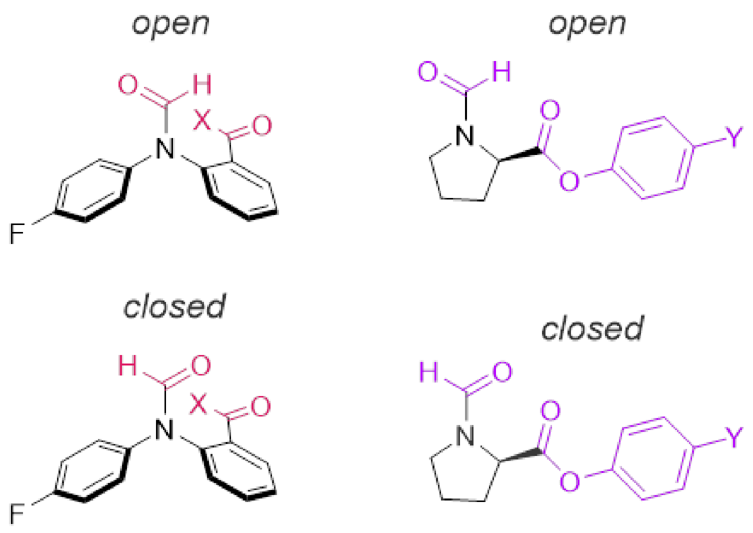

Figure S50 Defining the geometry for intramolecular fiSAPT calculations for balance series **1**-X and **3**-Y. Colours define the interacting functional groups, while black bonds define the linking groups. Balance **2** was treated with a comparable intermolecular SAPT analysis (see **Figure S54**).

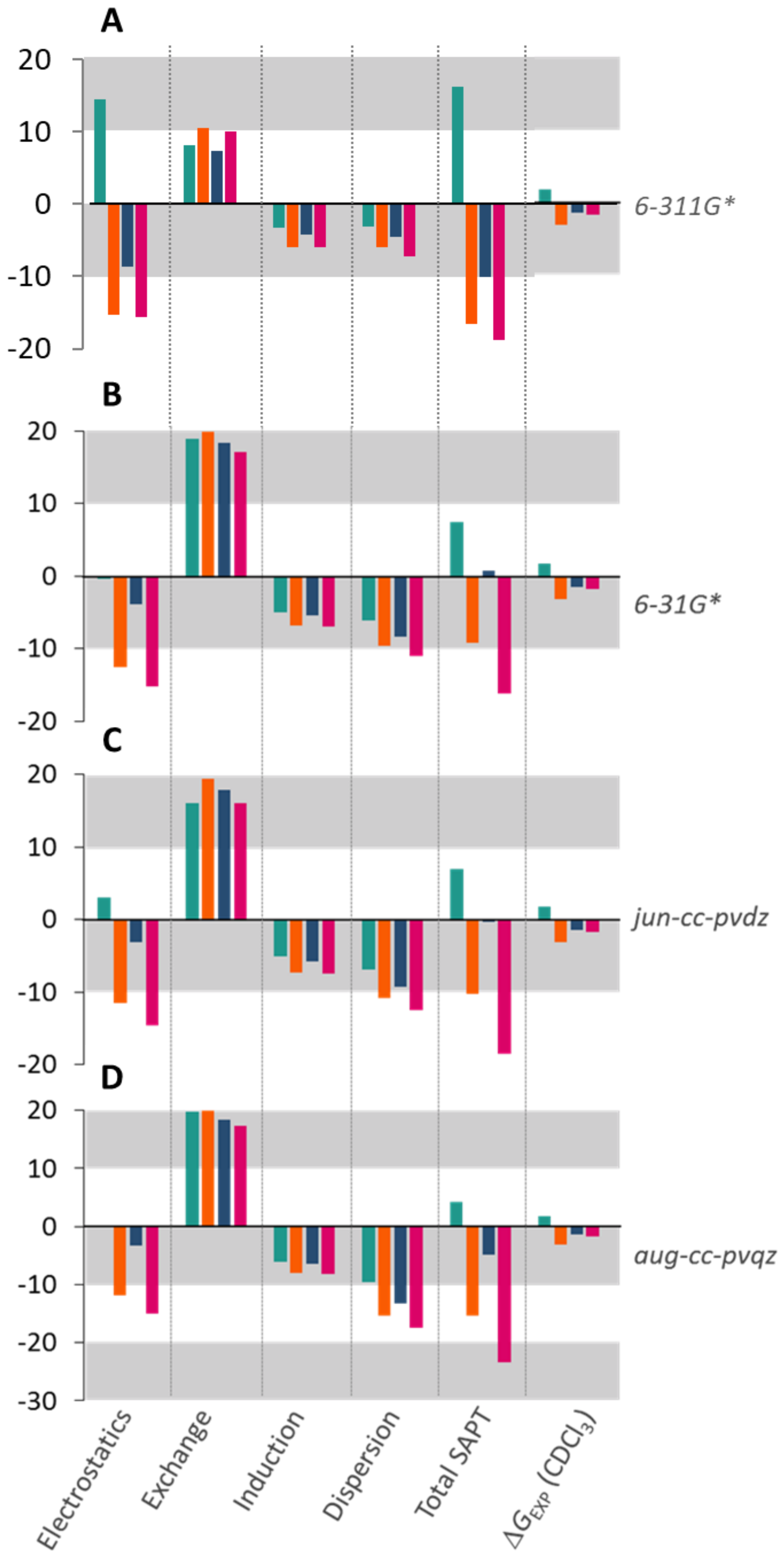

Figure S51 Closed conformation predicted fiSAPT contributions for molecular balances **1**-H (teal), **1**-Me (orange), **1**-OMe (blue) and **1**-NMe_2 (pink) using DFT/B3LYP/6-31G* minimised geometries. Calculated using the SAPT0 methodology and basis set **A** 6-311G*, **B** 6-31G*, **C** jun-cc-pVDZ and **D** aug-cc-pVQZ.

A further fiSAPT analysis was performed on the ωB97X-D/6-31G* minimised structures of the closed conformations of balance series **1**-X that included dispersion correction. This resulted in only minor changes to the components and total SAPT interaction energy compared to the B3LYP-minimised structures (**Figure S52**, *c.f.* **Figure S51**).

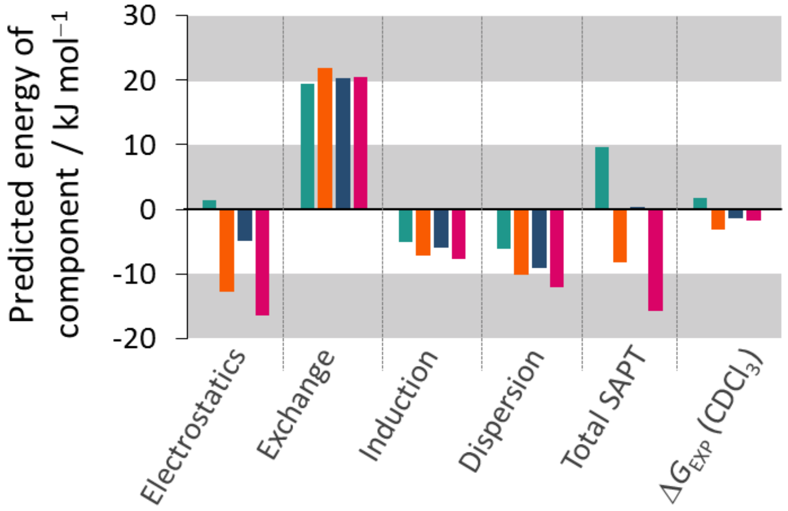

Figure S52 Closed conformation predicted fiSAPT contributions of molecular balances **1**-H (teal), **1**-Me (orange), **1**-OMe (blue) and **1**-NMe_2 (pink) using DFT/ωB97X-D/6-31G* minimised geometries. Calculated using PSI4 SAPT0/6-311G*.

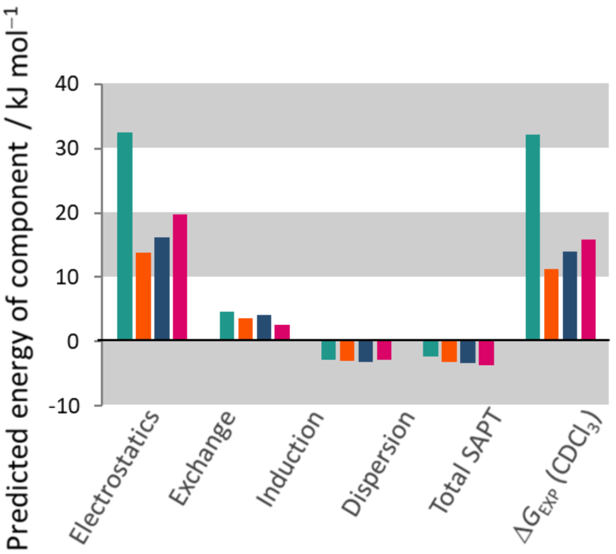

Figure S53 Open conformation fiSAPT energy contributions of molecular balances **1**-H (teal), **1**-Me (orange), **1**-OMe (blue) and **1**-NMe_2 (pink) using DFT/B3LYP/6-31G* minimised geometries. Calculated using PSI4 SAPT0/6-311G*.

Table S25 Energetic contributions of the interaction seen in molecular balance series **1**-X in the open and closed conformations. Calculated using PSI4 SAPT0/6-311G* using geometry minimised balances from Spartan '14 DFT/B3LYP/6-31G*.

Compound	Electrostatics / kJ mol^{-1}	Exchange / kJ mol^{-1}	Induction / kJ mol^{-1}	Dispersion / kJ mol^{-1}	Total SAPT / kJ mol^{-1}
open **1**-H	32.53	4.71	−2.78	−2.37	32.09
open **1**-Me	13.82	3.57	−3.00	−3.12	11.27
open **1**-OMe	16.25	4.14	−3.19	−3.25	13.94
open **1**-NMe_2	19.83	2.58	−2.81	−3.68	15.91
closed **1**-H	14.49	8.18	−3.20	−3.19	16.27
closed **1**-Me	−15.26	10.57	−5.91	−5.93	−16.52
closed **1**-OMe	−8.65	7.42	−4.28	−4.58	−10.09
closed **1**-NMe_2	−15.71	10.01	−5.90	−7.23	−18.84

Table S26 Energetic contributions of the interactions seen in the closed conformer of molecular balance **1**-H. Calculated using PSI4 SAPT0 with various basis sets using geometry minimised balances from Spartan '14 DFT/B3LYP/6-31G*.

1-H Basis set	Electrostatics / kJ mol^{-1}	Exchange / kJ mol^{-1}	Induction / kJ mol^{-1}	Dispersion / kJ mol^{-1}	Total SAPT / kJ mol^{-1}
6-311G*	15.40	9.36	−4.11	−3.64	17.01
jun-cc-PTDZ	16.92	8.89	−4.58	−4.07	17.15
6-311+G**	17.10	9.13	−4.67	−4.24	17.33

Table S27 Energetic contributions of the interactions seen in the closed conformer of molecular balance **1**-H. Calculated using PSI4 SAPT0 with various basis sets using the geometry from the crystal structure.

1-H Basis set	Electrostatics / kJ mol^{-1}	Exchange / kJ mol^{-1}	Induction / kJ mol^{-1}	Dispersion / kJ mol^{-1}	Total SAPT / kJ mol^{-1}
6-311G*	15.40	9.36	−4.11	−3.64	17.01
jun-cc-PTDZ	16.92	8.89	−4.58	−4.07	17.15
6-311+G**	17.10	9.13	−4.67	−4.24	17.33

Table S28 Closed conformation energetic contributions of the interaction seen in molecular balance series **1**-X and **2**. Calculated using PSI4 SAPT0/6-31G* using geometry minimised balances from Spartan ’14 DFT/B3LYP/6-31G*.

Compound	Electrostatics / kJ mol^{-1}	Exchange / kJ mol^{-1}	Induction / kJ mol^{-1}	Dispersion / kJ mol^{-1}	Total SAPT / kJ mol^{-1}
**1**-H	−0.42	18.91	−4.99	−6.04	7.45
**1**-Me	−12.60	19.90	−6.78	−9.63	−9.12
**1**-OMe	−3.88	18.39	−5.44	−8.32	0.76
**1**-NMe_2	−15.23	17.13	−6.99	−11.04	−16.12

Table S29 Closed conformation energetic contributions of the interaction seen in molecular balance series **1**-X. Calculated using PSI4 SAPT0/jun-cc-pVDZ using geometry minimised balances from Spartan ’14 DFT/B3LYP/6-31G*.

Compound	Electrostatics / kJ mol^{-1}	Exchange / kJ mol^{-1}	Induction / kJ mol^{-1}	Dispersion / kJ mol^{-1}	Total SAPT / kJ mol^{-1}
**1**-H	3.05	15.97	−5.12	−6.95	6.94
**1**-Me	−11.45	19.42	−7.36	−10.81	−10.19
**1**-OMe	−3.10	17.78	−5.79	−9.23	−0.34
**1**-NMe_2	−14.52	16.07	−7.47	−12.51	−18.43

Table S30 Closed conformation energetic contributions of the interaction seen in molecular balance series **1**-X. Calculated using PSI4 SAPT0/aug-cc-pVQZ using geometry minimised balances from Spartan ’14 DFT/B3LYP/6-31G*.

Compound	Electrostatics / kJ mol^{-1}	Exchange / kJ mol^{-1}	Induction / kJ mol^{-1}	Dispersion / kJ mol^{-1}	Total SAPT / kJ mol^{-1}
**1**-H	0.10	19.78	−6.04	−9.64	4.21
**1**-Me	−11.90	19.86	−8.00	−15.29	−15.33
**1**-OMe	−3.35	18.31	−6.47	−13.30	−4.81
**1**-NMe_2	−15.04	17.34	−8.22	−17.41	−23.33

Table S31 Energetic contributions of the interactions seen in molecular balance series **1**-X. Calculated using PSI4 SAPT0/6-311G* using geometry minimised balances from Spartan ’14 DFT/ωB97X-D/6-31G*.

Compound	Electrostatics / kJ mol^{-1}	Exchange / kJ mol^{-1}	Induction / kJ mol^{-1}	Dispersion / kJ mol^{-1}	Total SAPT / kJ mol^{-1}
**1**-H	1.34	19.38	−4.99	−6.14	9.60
**1**-Me	−12.80	21.86	−7.20	−10.09	−8.23
**1**-OMe	−4.94	20.31	−5.94	−9.04	0.39
**1**-NMe_2	−16.48	20.55	−7.64	−12.15	−15.72

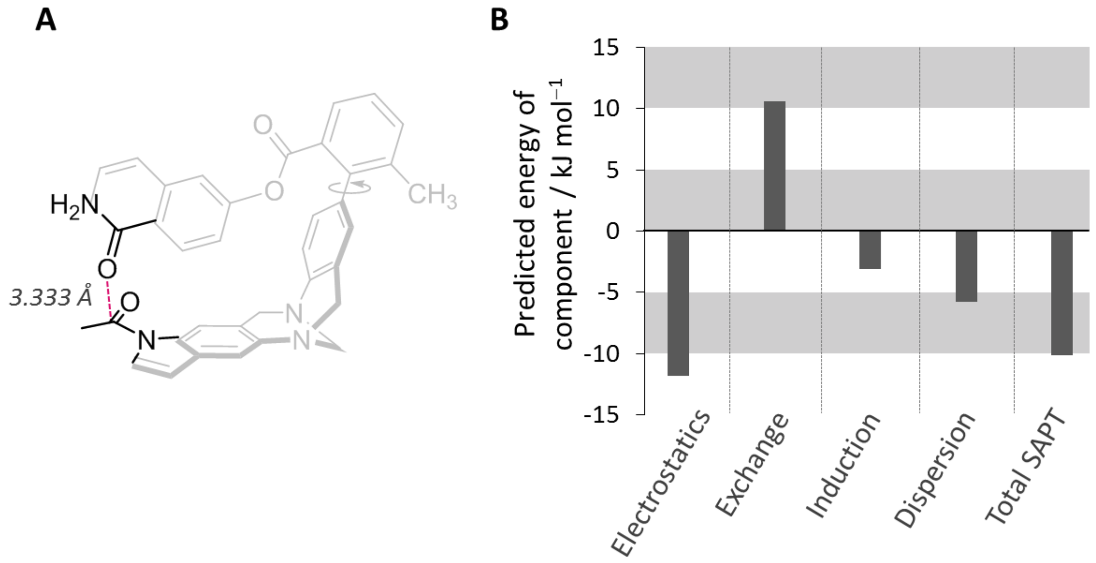

Figure S54 **A** Structure of the two interacting fragments used in the SAPT analysis of carbonyl···carbonyl interactions isolated from Diederich's molecular balance **2**. The fragment geometry was identical to the known crystal structure of balance **2**. **B** Energetic contributions of the interaction terms in the depicted molecular fragment. Calculated using PSI4 SAPT0/6-311G*.

Table S32 Energetic contributions of the fragment depicted in Figure S54 dervied from the known X-ray structure of balance **2**. Calculated using PSI4 SAPT0/6-311G*.

Compound	Electrostatics / kJ mol^{-1}	Exchange / kJ mol^{-1}	Induction / kJ mol^{-1}	Dispersion / kJ mol^{-1}	Total SAPT / kJ mol^{-1}
**2**	−11.81	10.58	−3.07	−5.81	−10.12

Table S33 Energetic contributions of the interaction seen in molecular balance series **3**-Y in the open and closed conformations. Calculated using PSI4 SAPT0/6-311G* using geometry minimised balances from Spartan ’14 DFT/B3LYP/6-31G*.

Compound	Electrostatics / kJ mol^{-1}	Exchange / kJ mol^{-1}	Induction / kJ mol^{-1}	Dispersion / kJ mol^{-1}	Total SAPT / kJ mol^{-1}
open **3**-NO_2	43.10	5.12	−4.15	−2.78	41.29
open **3**-CN	41.10	5.26	−4.08	−2.79	39.49
open **3**-H	35.18	6.38	−4.03	−2.92	34.60
open **3**-OMe	34.86	3.29	−3.63	−2.18	32.33
open **3**-NMe_2	33.55	2.80	−3.48	−1.96	30.92
closed **3**-NO_2	16.76	14.71	−8.37	−7.43	15.68
closed **3**-CN	17.29	14.47	−8.17	−7.40	16.19
closed **3**-H	19.96	12.43	−7.17	−6.82	18.40
closed **3**-OMe	19.25	9.68	−6.17	−5.68	17.07
closed **3**-NMe_2	19.89	8.59	−5.67	−5.23	17.57

Table S34 Energetic contributions of the interactions seen in the closed conformer of molecular balance **3**-NO_2. Calculated using PSI4 SAPT0 with various basis sets using geometry minimised balances from Spartan ’14 DFT/B3LYP with various basis sets.

SAPT0/ basis set	Geometry optimized with B3LYP/ basis set	Electrostatics / kJ mol^{-1}	Exchange / kJ mol^{-1}	Induction / kJ mol^{-1}	Dispersion / kJ mol^{-1}	Total SAPT / kJ mol^{-1}
6-311G*	6-31G*	16.76	14.71	-8.37	-7.43	15.68
jun-cc-pVDZ	jun-cc-pVDZ	21.22	8.63	-7.00	-6.16	16.69
6-311+G**	6-311+G**	20.99	9.40	-7.18	-6.41	16.80

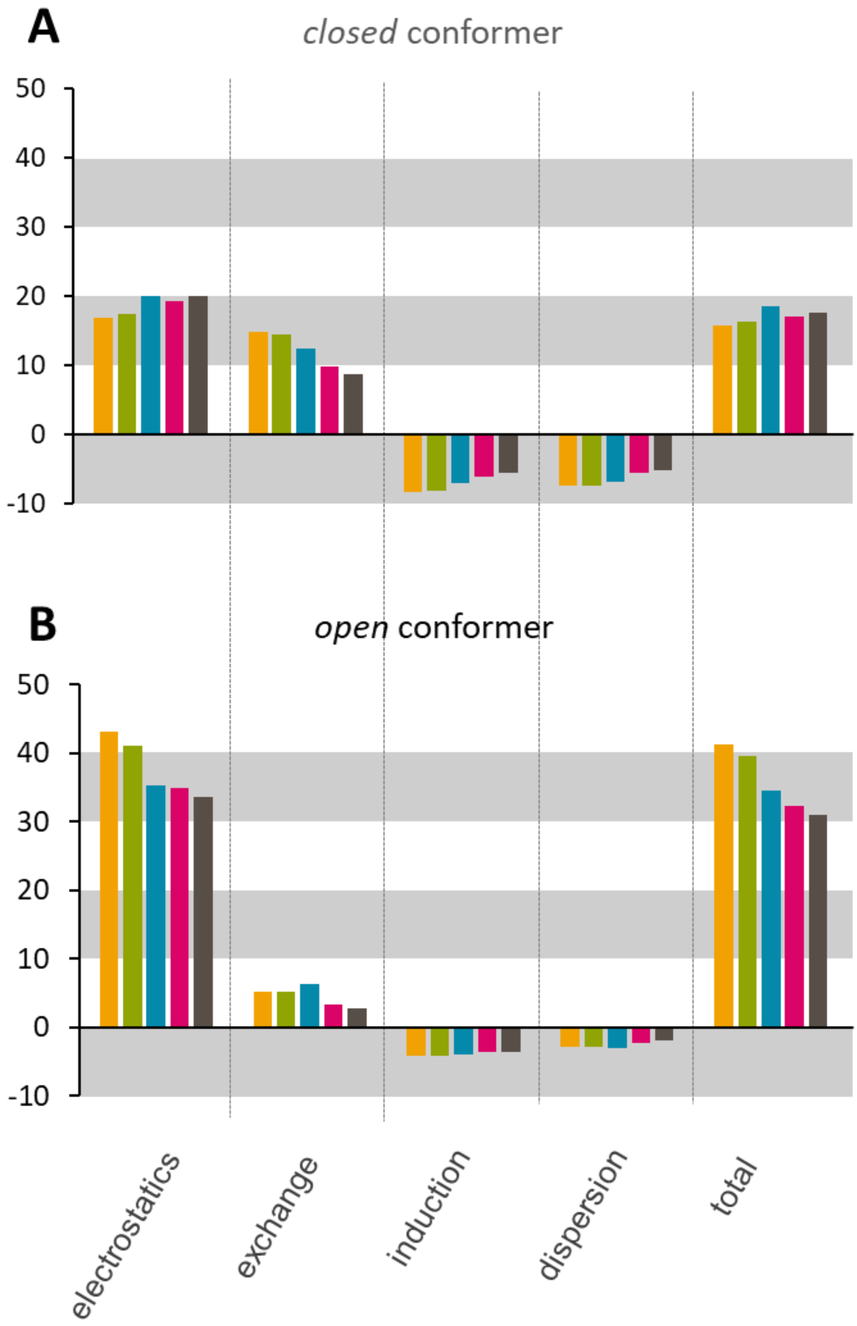

Figure S55 **A** Open and **B** close conformation fiSAPT energy contributions of molecular balances **3**-NO_2 (yellow), **3**-CN (green), **3**-H (blue), **3**-OMe (pink) and **3**-NMe_2 (dark grey) using DFT/B3LYP/6-31G* minimised geometries. Calculated using PSI4 SAPT0/6-311G*.

Distance-dependant SAPT

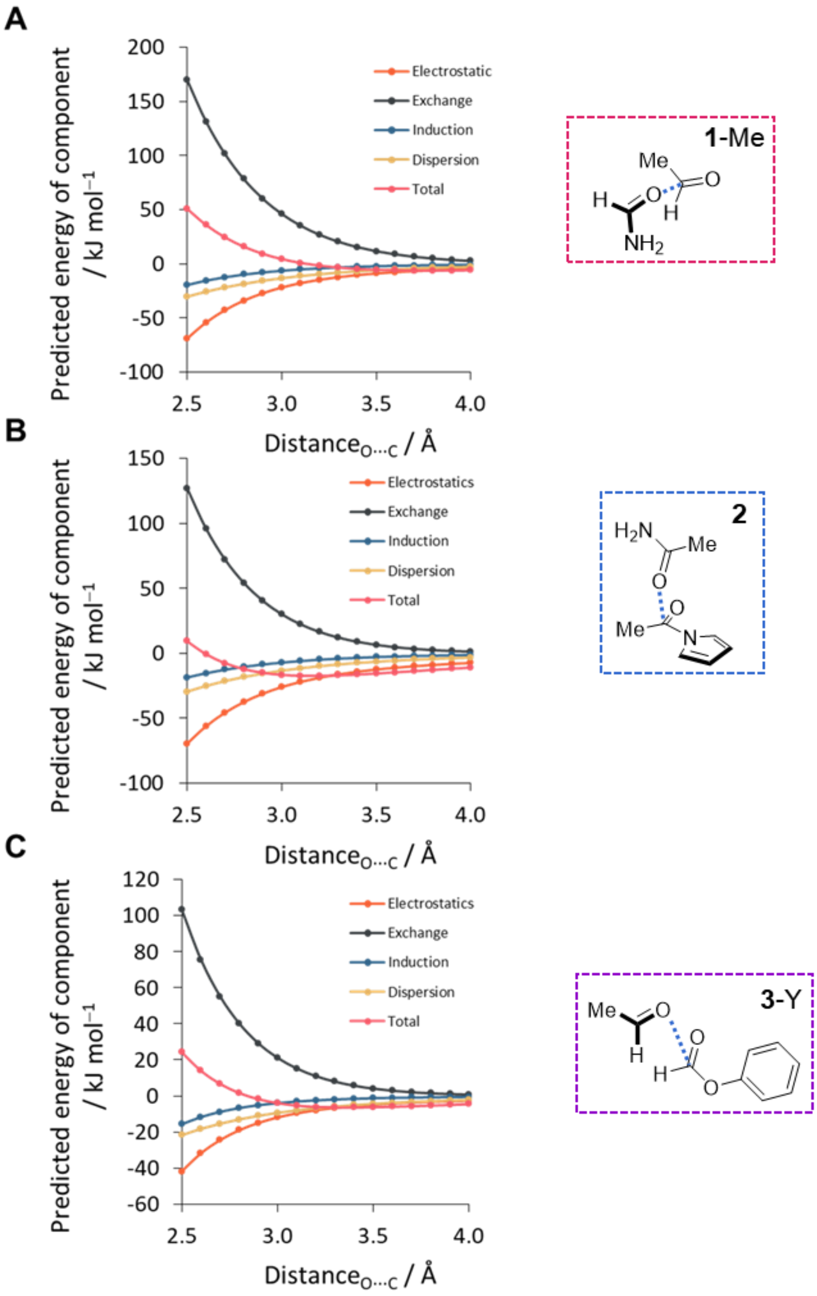

Figure S56 Energetic contributions of the interactions seen in the isolated fragments of balances **A 1**-H, **B 2** and **C 3**-H as the O⋯C distance was varied (insets). The C=O⋯C angle was maintained at as determined from the minimised structure of balances **1**-X and **3**-Y and the X-ray structure of **2**, **A 1**-Me 100.79°, **B 2** 109.33°, **C 3**-H 99.78°. Calculated using PSI4 SAPT0/6-31G*, raw data in **Tables S35–S37**).

Table S35 Energetic contributions of the interactions seen in the isolated components of balance **1**-Me as the O···C distance was varied (**Figure S56**). The O···C=O angle was maintained at 100.79° as determined from the minimised structure of the whole balance. Calculated using PSI4 SAPT0/6-31G*.

Distance $_{O \cdots C}$ / Å	Electrostatics / kJ mol^{-1}	Exchange / kJ mol^{-1}	Induction / kJ mol^{-1}	Dispersion / kJ mol^{-1}	Total SAPT / kJ mol^{-1}
4.00	−4.96	2.62	−0.88	−2.81	−6.03
3.90	−5.46	3.56	−1.05	−3.25	−6.19
3.80	−6.06	4.82	−1.26	−3.77	−6.27
3.70	−6.81	6.50	−1.52	−4.39	−6.23
3.60	−7.75	8.70	−1.85	−5.12	−6.01
3.50	−8.92	11.61	−2.25	−5.98	−5.55
3.40	−10.42	15.41	−2.76	−7.00	−4.77
3.30	−12.32	20.37	−3.39	−8.21	−3.55
3.20	−14.76	26.83	−4.18	−9.64	−1.75
3.10	−17.90	35.22	−5.17	−11.33	0.82
3.00	−21.96	46.09	−6.41	−13.33	4.38
2.90	−27.21	60.13	−7.97	−15.70	9.24
2.80	−34.01	78.24	−9.94	−18.51	15.78
2.70	−42.82	101.54	−12.43	−21.82	24.47
2.60	−54.25	131.49	−15.59	−25.75	35.91
2.50	−69.07	169.95	−19.64	−30.40	50.84

Table S36 Energetic contributions of the interactions seen in the isolated components of balance **2** as the O···C distance was varied (**Figure S56**). The O···C=O angle was maintained at 109.33° as determined from the X-ray structure of the whole balance. The interaction is decomposed into electrostatics, exchange-repulsion, induction and dispersion to give a total SAPT predicted interaction energy. Calculated using PSI4 SAPT0/6-31G*.

Distance $_{O\cdots C}$ / Å	Electrostatics / kJ mol^{-1}	Exchange / kJ mol^{-1}	Induction / kJ mol^{-1}	Dispersion / kJ mol^{-1}	Total SAPT / kJ mol^{-1}
4.00	−7.49	1.21	−1.36	−3.38	−11.02
3.90	−8.20	1.71	−1.58	−3.83	−11.90
3.80	−9.02	2.40	−1.84	−4.36	−12.81
3.70	−9.98	3.35	−2.15	−4.96	−13.75
3.60	−11.14	4.64	−2.53	−5.67	−14.70
3.50	−12.52	6.41	−2.98	−6.50	−15.60
3.40	−14.21	8.80	−3.53	−7.47	−16.41
3.30	−16.28	12.04	−4.19	−8.62	−17.05
3.20	−18.85	16.40	−5.00	−9.96	−17.41
3.10	−22.06	22.24	−5.98	−11.55	−17.35
3.00	−26.09	30.04	−7.18	−13.42	−16.65
2.90	−31.17	40.42	−8.65	−15.65	−15.04
2.80	−37.61	54.17	−10.45	−18.28	−12.18
2.70	−45.81	72.29	−12.66	−21.42	−7.61
2.60	−56.26	96.06	−15.36	−25.16	−0.72
2.50	−69.59	127.10	−18.68	−29.61	9.21

Table S37 Energetic contributions of the interactions seen in the isolated components of balance **3**-H as the O···C distance was varied (**Figure S56**). The O···C=O angle was maintained at 99.78° as determined from the minimised structure of the whole balance. The interaction is decomposed into electrostatics, exchange-repulsion, induction and dispersion to give a total SAPT predicted interaction energy. Calculated using PSI4 SAPT0/6-31G*.

Distance $_{O\cdots C}$ / Å	Electrostatics / kJ mol^{-1}	Exchange / kJ mol^{-1}	Induction / kJ mol^{-1}	Dispersion / kJ mol^{-1}	Total SAPT / kJ mol^{-1}
4.00	−2.63	0.76	−0.50	−2.20	−4.57
3.90	−2.90	1.06	−0.59	−2.50	−4.93
3.80	−3.23	1.49	−0.70	−2.85	−5.30
3.70	−3.64	2.09	−0.85	−3.27	−5.65
3.60	−4.13	2.93	−1.02	−3.75	−5.98
3.50	−4.75	4.09	−1.25	−4.33	−6.25
3.40	−5.54	5.70	−1.54	−5.01	−6.40
3.30	−6.56	7.93	−1.91	−5.82	−6.36
3.20	−7.87	11.02	−2.41	−6.79	−6.04
3.10	−9.59	15.29	−3.06	−7.94	−5.30
3.00	−11.87	21.16	−3.92	−9.32	−3.96
2.90	−14.90	29.21	−5.09	−10.97	−1.75
2.80	−18.96	40.23	−6.66	−12.95	1.66
2.70	−24.41	55.26	−8.79	−15.34	6.71
2.60	−31.78	75.68	−11.69	−18.23	13.99
2.50	−41.74	103.33	−15.65	−21.72	24.22

S4.4 Natural Bond Orbital analysis

Natural bond orbital (NBO) analyses were performed using a single point energy calculation using Gaussian 09 Revision E.01[S15] at DFT/B3LYP/6-31G* using the geometry from the already minimised full molecular balance from Spartan ’14 (**Section S4.1**) to generate an NBO output. The NBO output was then inputted to NBO 6.0[S16] to obtain second-order perturbation theory output energies, images created using JmolNbo Visualization Helper Version 2.0 and Jmol or Chemcraft.

Table S388 NBO second-order perturbation energies observed in minimised structure for balance **1**-Me and balance series **3**-Y. Other balances in series **1**-X and balance **2** did not show NBOs. The sum total lone pair n→π* interaction energy is also given. Calculated using single point energy calculation using Gaussian 09 Revision E.013 at DFT/B3LYP/6-31G*. Second-order perturbation theory output energies calculated using NBO 6.0

		Second Order Perturbation Energy, $E^{(2)}_{n\rightarrow\pi^*}$ /kJ mol⁻¹		
**Compound**	**Geometry**	**LP (1) → π***	**LP (2) → π***	**LP (1+2) → π***
**1**-Me	Crystal structure	2.6	3.7	6.3
**3**-NO_2	Minimised (B3LYP/6-31G*)		9.37	9.37
**3**-CN	Minimised (B3LYP/6-31G*)		9.12	9.12
**3**-H	Minimised (B3LYP/6-31G*)		7.49	7.49
**3**-OMe	Minimised (B3LYP/6-31G*)		5.19	5.19
**3**-NMe_2	Minimised (B3LYP/6-31G*)		4.44	4.44

Table S39 NBO second-order perturbation energies observed in minimised structure for balance **3**-NO_2. Calculated using single point energy calculation using Gaussian 09 Revision E.013 at DFT/B3LYP with different basis sets. Second-order perturbation theory output energies calculated using NBO 6.0

	Second Order Perturbation Energy, $E^{(2)}_{n\rightarrow\pi^*}$ /kJ mol⁻¹		
**Basis set for geometry optimisation and NBO analysis**	**LP (1) → π***	**LP (2) → π***	**LP (1+2) → π***
6-311G*		9.37	9.37
jun-cc-pVDZ		6.11	6.11
6-311+G**		5.98	5.98

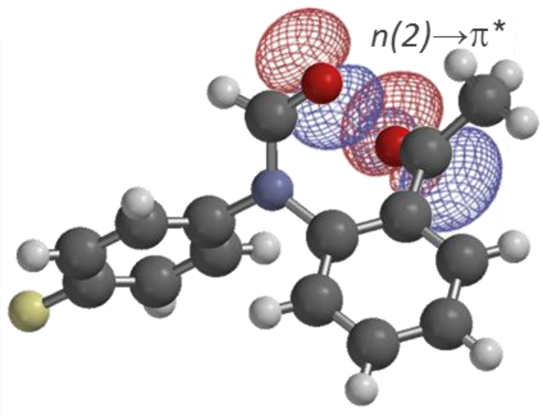

Figure S577 Diagram of predicted electron delocalisation from the lone pair of the oxygen formyl into the antibonding π* bond of the C=O group of compound **1**-Me. **1**-Me has the shortest O···C distance of the balances in series **1**-X (**Table S20**). Calculated from the crystal structure geometry (distance$_{O\cdots C}$ = 2.784 Å) using a using Gaussian ’09 and NBO 6.0. NBO analysis on the ωB97X-D/6-31G* minimised structures also revealed n→π* electron delocalisation to occur **1**-Me worth 6.3 kJ mol^{-1}.

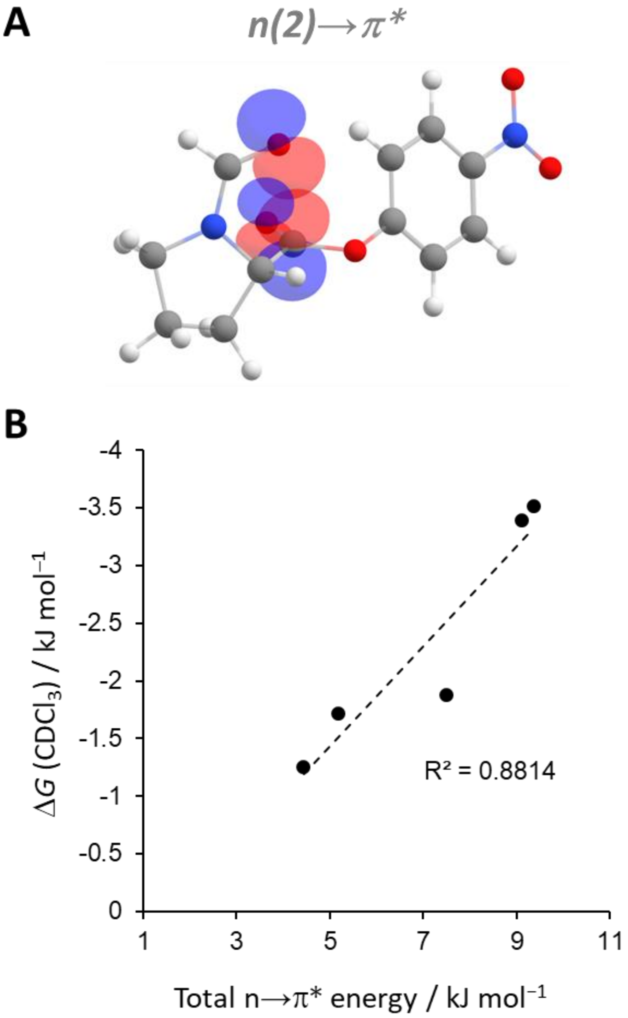

Figure S588 **A**, NBO corresponding to n→π* electron delocalisation from both lone pairs of the amide donor in molecular balances from series **3**-Y. **B** Plot of predicted stabilisation energies from NBO second-order energy perturbations from n→π* electron delocalisation against experimentally measured conformational free energy difference determined by ^{1}H NMR spectroscopy ($CDCl_3$, 400 MHz, 298 K). Experimental values taken from reference S3. NBO calculations performed using Gaussian '09 and NBO 6.0 B3LYP/6-31G*.

Table S40 Distance dependence on NBO second-order perturbation energies in molecular balance **1**-Me. The O···C=O angle was maintained at 100.8°, as seen in the minimised structure (**Table S20**).

**Distance $_{O\cdots C}$ / Å**	**Second Order Perturbation Energy, $E^{(2)}_{n\rightarrow\pi^*}$ /kJ mol^{-1}** LP (1) → π*	LP (2) → π*	LP (1+2) → π*
3.5	0	−0.46	−0.46
3.4	0	−0.62	−0.62
3.3	0	−0.92	−0.92
3.2	−0.20	−1.33	−1.54
3.1	−0.37	−1.88	−2.25
3.0	−0.58	−2.67	−3.26
2.9	−0.92	−3.76	−4.68
2.8	−1.42	−5.23	−6.65
2.7	−2.21	−7.28	−9.49
2.6	−3.38	−10.04	−13.430
2.5	−5.10	−13.80	−18.91

Table S41 Angle dependence on NBO second-order perturbation energies in molecular balance **1**-Me. The O···C distance was maintained at 2.964 Å, as seen in the minimised structure (**Table S20**).

**Angle $_{O\cdots C=O}$ / °**	**Second Order Perturbation Energy, $E^{(2)}_{n\rightarrow\pi^*}$ /kJ mol^{-1}** LP (1) → π*	LP (2) → π*	LP (1+2) → π*
80	−0.96	−3.43	−4.39
85	−0.92	−3.55	−4.47
90	−0.87	−3.51	−4.39
95	−0.79	−3.34	−4.14
100	−0.71	−3.09	−3.80
105	−0.58	−2.76	−3.34
110	−0.54	−2.63	−3.17
115	−0.50	−2.51	−3.01
120	−0.25	−1.58	−1.84

Table S42 Distance dependence on NBO second-order perturbation energies in molecular balance **2**. The O···C=O angle was maintained at 109.3°, as found in the known X-ray structure (**Table S20**).

**Distance $_{O\cdots C}$ / Å**	**Second Order Perturbation Energy, $E^{(2)}_{n\rightarrow\pi^*}$ /kJ mol^{-1}** LP (1) → π*	LP (2) → π*	LP (1+2) → π*
3.5	−0.29	−0.20	−0.50
3.4	−0.46	−0.29	−0.75
3.3	−0.71	−0.41	−1.12
3.2	−1.04	−0.58	−1.63
3.1	−1.50	−0.79	−2.30
3.0	−2.25	−1.04	−3.30
2.9	−3.30	−1.42	−4.72
2.8	−4.85	−1.88	−6.73
2.7	−7.07	−2.46	−9.53
2.6	−10.25	−3.30	−13.55
2.5	−14.85	−4.39	−19.24

Table S43 Angle dependence on NBO second-order perturbation energies in molecular balance **2**. The O···C distance was maintained at 3.333 Å, as found in the known X-ray structure (**Table S20**).

**Angle $_{O\cdots C=O}$ / °**	**Second Order Perturbation Energy, $E^{(2)}_{n\rightarrow\pi^*}$ /kJ mol^{-1}** LP (1) → π*	LP (2) → π*	LP (1+2) → π*
80	−0.58	0	−0.58
85	−0.66	−0.29	−0.96
90	−0.75	−0.33	−1.08
95	−0.75	−0.37	−1.12
100	−0.71	−0.41	−1.12
105	−0.66	−0.37	−1.04
110	−0.58	−0.37	−0.96
115	−0.50	−0.33	−0.83
120	−0.41	−0.29	−0.71

Table S44 Distance dependence on NBO second-order perturbation energies in molecular balance **3**-H. The O···C=O angle was maintained at 99.8°, as seen in the minimised structure (**Table S20**).

**Distance $_{O\cdots C}$ / Å**	**Second Order Perturbation Energy, $E^{(2)}_{n\rightarrow\pi^*}$ /kJ mol^{-1}**		
	**LP (1) → π***	**LP (2) → π***	**LP (1+2) → π***
3.5	0	−0.29	−0.29
3.4	0	−0.46	−0.46
3.3	0	−0.71	−0.71
3.2	0	−1.04	−1.04
3.1	−0.20	−1.63	−1.84
3.0	−0.37	−2.46	−2.84
2.9	−0.62	−3.80	−4.43
2.8	−1.04	−5.98	−7.02
2.7	−1.71	−9.37	−11.08
2.6	−2.76	−14.64	−17.40
2.5	−4.39	−22.34	−26.73

Table S45 Angle dependence on NBO second-order perturbation energies in molecular balance **3**-H. The O···C distance was maintained at 2.741 Å, as seen in the minimised structure (**Table S20**).

**Angle $_{O\cdots C=O}$ / °**	**Second Order Perturbation Energy, $E^{(2)}_{n\rightarrow\pi^*}$ /kJ mol^{-1}**		
	**LP (1) → π***	**LP (2) → π***	**LP (1+2) → π***
80	−1.29	−8.24	−9.53
85	−1.29	−7.90	−9.20
90	−1.29	−7.53	−8.82
95	−1.33	−7.61	−8.95
100	−1.38	−7.78	−9.16
105	−1.42	−7.86	−9.28
110	−1.42	−7.78	−9.20
115	−1.38	−7.44	−8.82
120	−1.29	−6.90	−8.20

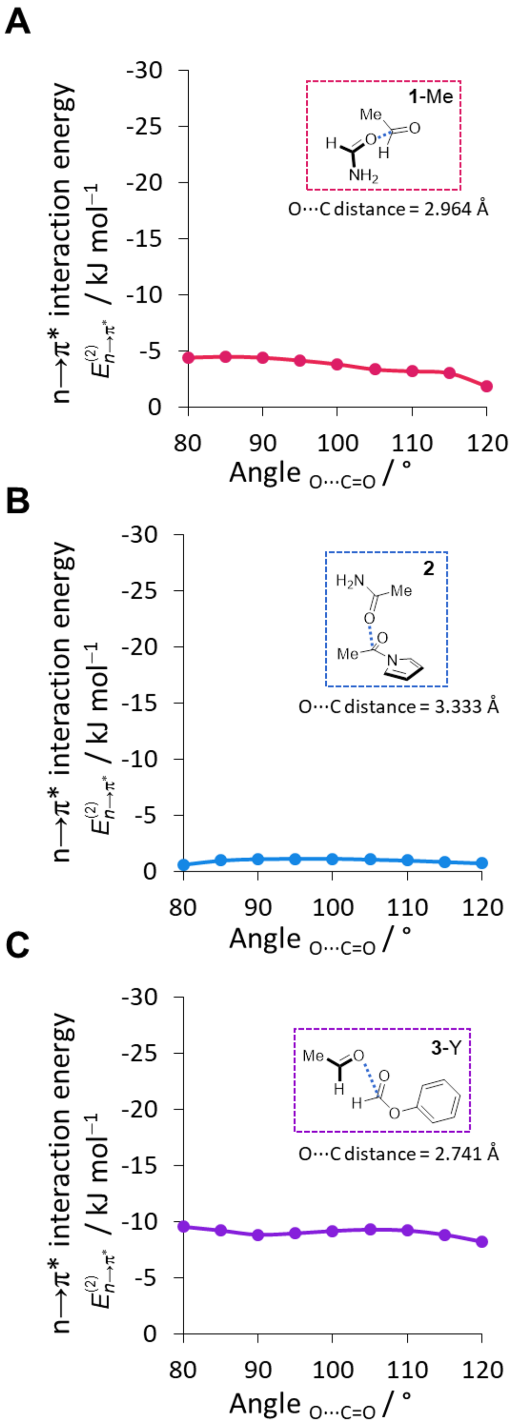

Figure S59 O···C=O angle dependence of the sums of the 2nd order perturbation energies corresponding to n→π* electron delocalisation of both lone pairs in carbonyl-carbonyl dimers modelling the equivalent interactions hosted within molecular balances **A** **1**-Me **B** **2**, and **C** **3**-H. Energies were calculated using NBO6.0 (see **Tables S41**, **S43**, **S45**).

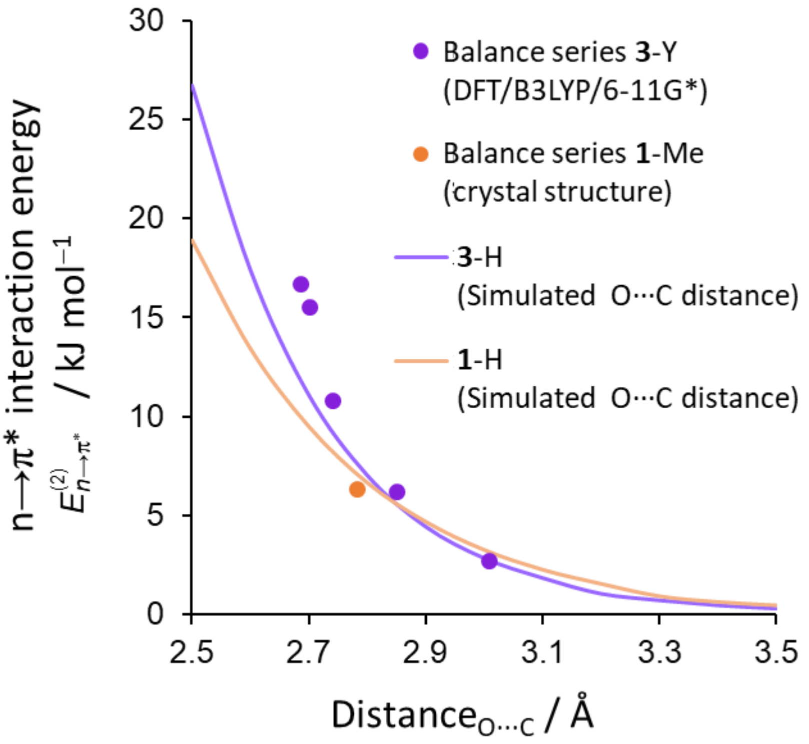

Figure S60 Distance dependence of the sums of the 2nd order perturbation energies corresponding to n→π* electron delocalisation of both lone pairs in carbonyl-carbonyl dimers modelling the equivalent interactions hosted within molecular balance series **3**-Y and balance **1**-Me. Simulated trends relate to data presented in **Table S40** and **S44**, where the O···C distance was artificially varied starting from the B3LYP/6-31G* minimised geometry of balance **1**-Me and **3**-H respectively (all other angles/distances kept constant). Energies were calculated using NBO6.0).

Figure S60 compares the sums of the 2nd order perturbation energies corresponding to n→π* electron delocalisation of both lone pairs in carbonyl-carbonyl dimers across balance series **3**-Y and balance **1**-Me. The simulated trends relate to data presented in **Table S40** and **S44**, where the O···C distance was artificially varied starting from the B3LYP/6-31G* minimised geometry of balance **1**-Me and **3**-H respectively (all other angles/distances kept constant).

The deviation of the data points for balance series **3**-Y (purple) from the simulated trend reflect electronic/steric differences arising from the different substituents. Likewise, the deviation of the data point for balance **1**-Me (orange) from the simulated data reflect differences between the crystal structure geometry and simulated geometry based on B3LYP/6-31G* minimisation. **Figure S59** demonstrates that the O···C=O angle has minimal effect on the 2nd order perturbation energies.

Given this variation as a result of precise structure/substitution, the NBO analysis of balance **1**-Me fits remarkably well with the trend seen for balance series **3**-Y, with the O···C distance being the dominant factor for determining the 2nd order perturbation energies.

S4.5 Molecular orbital analysis

A detailed molecular orbital analysis was performed in this study in which the orbital energies of open and closed conformers of the molecular balances were compared. To enable identification and pairing of molecular orbitals found in the open and closed conformers it was necessary to avoid orbital splitting arising from the canonical resonance forms of the aromatic electrons (that were not involved in the interactions of interest).

The minimised full molecular balance structures of series were subsequently used to generate simplified balance structures from series **1**-X of the type shown in **Figure S60**, in which the 4-fluorophenyl moiety was replaced with a proton with a N-H bond length of 1.012 Å. Fragment series ^{frag}**1**-X was found to reflect the full balance series **1**-X energies well (**Figure S62**).

For molecular balance series **3**-Y, the full molecular balance structures were used for the molecular orbital analyses.

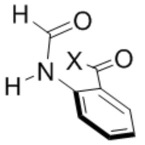

Figure S61 Simplified fragment of molecular balance series **1**-X used for molecular orbital analyses to generate fragment series ^{frag}**1**-X.

Table S46 Calculated conformational energy differences of simplified balance series ^{frag}**1**-X (**Figure S61**) from DFT calculations (Spartan '14, B3LYP/6-31G*).

Compound	Closed conformer *E* / kJ mol^{-1}	Open conformer *E* / kJ mol^{-1}	ΔE_{calc} / kJ mol^{-1}
^{frag}**1**-H	−1350179.59	−1350179.95	−0.36
^{frag}**1**-Me	−1453410.34	−1453415.90	−5.56
^{frag}**1**-OMe	−1650936.57	−1650937.51	−0.94
^{frag}**7**-NMe_2	−1701976.19	−1701988.18	−11.99

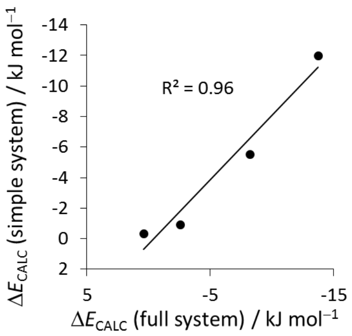

Figure S62 Plot of conformational energy differences between balance series **1**-X (ΔE_{calc} (full system)) and simplified fragment series ^{frag}**1**-X (ΔE_{calc} (simple system)) (Spartan '14, DFT/B3LYP/6-31G*).

Table S47 Molecular orbital energies of balances ^{frag}**1**-H, ^{frag}**1**-Me, ^{frag}**1**-OMe and ^{frag}**1**-NMe_2 in the open and closed conformers (Spartan ’14, DFT/B3LYP/6-31G*).

	Molecular Orbital Energies / eV							
	^{frag}**1**-H		^{frag}**1**-Me		^{frag}**1**-OMe		^{frag}**1**-NMe_2	
Molecular orbital	*open*	*closed*	*open*	*closed*	*open*	*closed*	*open*	*closed*
HOMO	−6.94	−6.78	−6.90	−6.54	−6.73	−6.58	−6.90	−6.21
HOMO {-1}	−7.21	−6.99	−6.98	−6.67	−6.99	−6.92	−6.98	−6.50
HOMO {-2}	−7.35	−7.18	−7.27	−7.03	−7.35	−7.06	−7.27	−6.64
HOMO {-3}	−7.57	−7.47	−7.44	−7.32	−7.75	−7.37	−7.44	−6.99
HOMO {-4}	−8.29	−8.16	−8.09	−7.96	−7.96	−7.93	−8.09	−7.26
HOMO {-5}	−10.19	−9.92	−9.72	−9.35	−8.36	−8.08	−9.72	−7.86
HOMO {-6}	−10.22	−9.99	−10.05	−9.90	−9.72	−9.59	−10.05	−9.50
HOMO {-7}	−10.57	−10.34	−10.21	−10.16	−10.13	−9.87	−10.21	−9.81
HOMO {-8}	−11.17	−10.98	−10.46	−10.30	−10.18	−9.92	−10.46	−9.97
HOMO {-9}	−11.36	−11.25	−11.01	−10.67	−10.39	−10.34	−11.01	−10.22

Table S48 Molecular orbital energies of balances **3**-NO_2, **3**-CN, 3-H, **3**-OMe and **3**-NMe_2 in the open and closed conformers (Spartan ’14, DFT/B3LYP/6-31G*). Orbitals in the closed conformation marked with asterisks do not originate from the equivalently numbered HOMO{-X} orbital in the open conformation (due to orbital interactions changing between conformers.

**Molecular Orbital Energies / eV**										
	**3**-NO_2		**3**-CN		**3**-H		**3**-OMe		**3**-NMe_2	
Molecular orbital	*open*	*closed*	*open*	*Closed*	*open*	*closed*	*open*	*closed*	*open*	*closed*
HOMO	−6.92	−7.07	−6.87	−6.90*	−6.60	−6.46*	−6.02	−5.78	−5.21	−5.02
HOMO {-1}	−7.00	−7.27*	−6.97	−7.06*	−6.70	−6.71*	−6.61	−6.69	−6.51	−6.56
HOMO {-2}	−7.53	−7.33*	−7.25	−7.29*	−6.78	−6.79*	−6.72	−6.83*	−6.65	−6.62*
HOMO {-3}	−7.87	−7.52	−7.79	−7.46	−7.09	−6.99*	−7.09	−6.92*	−6.84	−6.81*
HOMO {-4}	−8.03	−7.77	−8.36	−8.03	−7.92	−7.60	−7.82	−7.50	−7.58	−7.33
HOMO {-5}	−8.34	−8.09	−9.14	−8.79*	−9.04	−8.40	−8.48	−8.19	−7.66	−7.44
HOMO {-6}	−8.52	−8.24	−9.23	−8.87*	−9.59	−9.42*	−9.06	−8.66	−8.80	−8.30
HOMO {-7}	−8.55	−8.29	−9.80	−9.36	−9.72	−9.47*	−9.32	−8.94*	−9.42	−9.20
HOMO {-8}	−9.83	−9.05	−9.88	−9.62	−9.86	−9.70*	−9.54	−9.66*	−9.49	−9.43*
HOMO {-9}	−9.97	−9.98	−9.91	−9.95	−9.98	−9.82	−9.83	−9.69	−9.72	−9.62*

S4.6 Non-Covalent Interaction Plots

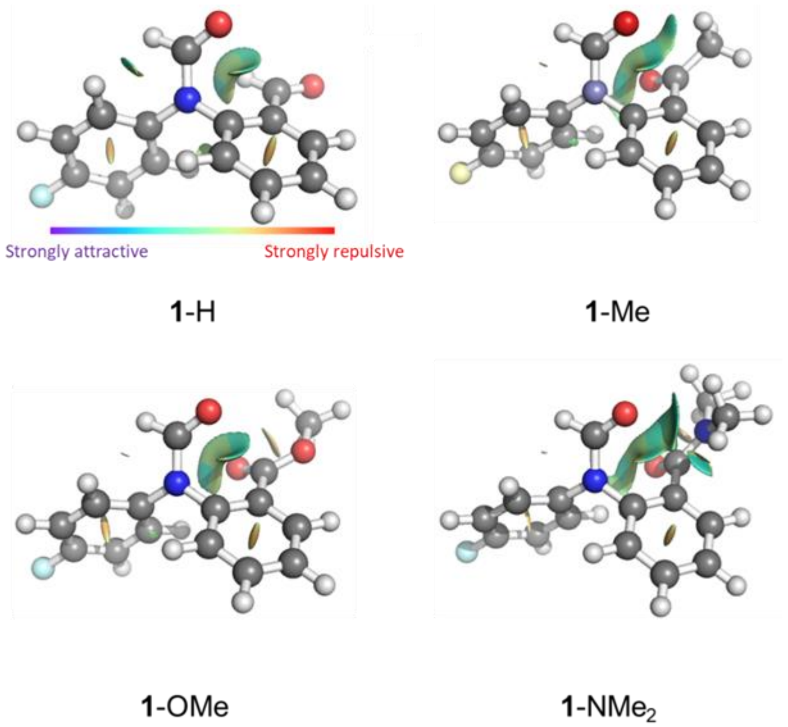

Figure S63 Non-covalent interactions (NCI) plots of balance series **1**-X. Density was calculated at PBE-D3BJ/Def2TZVP level of theory using Gaussian 09 Revision E.01[S15] to processed using the NCIplot program and visualised using Pymol. Blue areas denote attractive interactions; red areas denote repulsive interactions. Colour scale (blue) −0.05 < ρ < 0.05 au (red).

S5. References

[S1] I. K. Mati, C. Adam, S. L. Cockroft, *Chem. Sci.* **2013**, *4*, 3965–3972.

[S2] F. R. Fischer, P. A. Wood, F. H. Allen, F. Diederich, *Proc. Natl. Acad. Sci.* **2008**, *105*, 17290–17294.

[S3] J. A. Hodges, R. T. Raines, *Org. Lett.* **2006**, *8*, 4695–4697.

[S4] K. B. Muchowska, C. Adam, I. K. Mati, S. L. Cockroft, *J. Am. Chem. Soc.* **2013**, *135*, 9976–9979.

[S5] B. W. Gung, B. U. Emenike, M. Lewis, K. Kirschbaum, *Chem. Eur. J.* **2010**, *16*, 12357–12362.

[S6] M. Bauer, A. Bertario, G. Boccardi, X. Fontaine, R. Rao, D. Verrier, *J. Pharm. Biomed. Anal.* **1998**, *17*, 419–425.

[S7] a) C. A. Hunter, *Angew. Chem. Int. Ed.* **2004**, *43*, 5310–5324; *Angew. Chem.* **2004**, *116*, 5424–5439; b) R. Cabot, C. A. Hunter, L. M. Varley, *Org. Biomol. Chem.* **2010**, *8*, 1455–1462; c) L. Yang, C. Adam, G. S. Nichol, S. L. Cockroft, *Nat. Chem.* **2013**, *5*, 1006–1010.

[S8] A. A. Oliferenko, P. V. Oliferenko, J. G. Huddleston, R. D. Rogers, V. A. Palyulin, N. S. Zefirov, A. R. Katritzky, A. R. *J. Chem. Inf. Comp. Sci.* **2004**, *44*, 1042–1055.

[S9] D. R. Lide, in *CRC Handbook of Chemistry and Physics, 84 ed*. CRC Press, **2003**.

[S10] M. R. J. Dack, M. R. J. *Chem. Soc. Rev.* **1975**, *4*, 211–229.

[S11] J.-L. M. Abboud, R. Notari, *Pure Appl. Chem.* **1999**, *71*, 645–718.

[S12] C. M. Hansen, in *The Three Dimensional Solubility Parameter And Solvent Diffusion Coefficient*, Danish Technical Press, Copenhagen, **1967**, pp 18–19.

[S13] R. M. Parrish, L. A. Burns, D. G. A. Smith, A. C. Simmonett, A. E. DePrince III, E. G. Hohenstein. U. Bozkaya, A. Y. Sokolov, R. Di Remigio, R. M. Richard, J. F. Gonthier, A. M. James, H. R. McAlexander, A. Kumar, A. Saitow, X. Wang, B. P. Pritchard, P. Verma, H. F. Schaefer III, K. Patkowski, R. A. King, E. F. Valeev, F. A. Evangelista, J. M. Turney, T. D. Crawford, C. D. Sherrill, *J. Chem. Theory Comput.* **2017**, 13, 3185–3197.

[S14] R. M. Parrish, T. M. Parker, C. D. Sherrill, *J. Chem. Theory Comput.* **2014**, *10*, 4417–4431.

[S15] M. J. Frisch, G. W. Trucks, H. B. Schlegel, G. E. Scuseria, M. A. Robb, J. R. Cheeseman, G. Scalmani, V. Barone, G. A. Petersson, H. Nakatsuji, X. Li, M. Caricato, A. Marenich, J. Bloino, B. G. Janesko, R. Gomperts, B. Mennucci, H. P. Hratchian, J. V. Ortiz, A. F. Izmaylov, J. L. Sonnenberg, D. Williams-Young, F. Ding, F. Lipparini, F. Egidi, J. Goings, B. Peng, A. Petrone, T. Henderson, D. Ranasinghe, V. G. Zakrzewski, J. Gao, N. Rega, G. Zheng, W. Liang, M. Hada, M. Ehara, K. Toyota, R. Fukuda, J. Hasegawa, M. Ishida, T. Nakajima, Y. Honda, O. Kitao, H. Nakai, T. Vreven, K. Throssell, J. A. Montgomery, Jr., J. E. Peralta, F. Ogliaro, M. Bearpark, J. J. Heyd, E. Brothers, K. N. Kudin, V. N. Staroverov, T. Keith, R. Kobayashi, J. Normand, K. Raghavachari, A. Rendell, J. C. Burant, S. S. Iyengar, J. Tomasi, M. Cossi, J. M. Millam, M. Klene, C. Adamo, R. Cammi, J. W. Ochterski, R. L. Martin, K. Morokuma, O. Farkas, J. B. Foresman, and D. J. Fox, Gaussian 09, Revision E.01, Gaussian, Inc., Wallingford CT, **2016**.

[S16] E. D. Glendening, J, K. Badenhoop, A. E. Reed, J. E. Carpenter, J. A. Bohmann, C. M. Morales, P. Karafiloglou, C. R. Landis, and F. Weinhold, NBO 6.0 Theoretical Chemistry Institute, University of Wisconsin, Madison, **2013**.

[S17] E. R. Johnson, S. Keinan, P. Mori-Sánchez, J. Contreras-García, A. J. Cohen, W. Yang, *J. Am. Chem. Soc.* **2010**, 132, 6498-6506; J. Contreras-García, E. R. Johnson, S. Keinan, R. Chaudret, J.-P. Piquemal, D. N. Beratan, W. Yang, *J. Chem. Theory Comput.* **2011**, 7, 625-631.
